# Supplementary material for: Effectiveness of therapeutic ultrasound for the treatment of carpal tunnel syndrome (the USTINCTS trial): study protocol for a three-arm, prospective, multicentre, randomised controlled trial
Source: BMJ Open. 2022 Apr 13;12(4):e057541. doi: 10.1136/bmjopen-2021-057541 (PMC9014071; doi:10.1136/bmjopen-2021-057541)
Supplement: Supplementary data [file bmjopen-2021-057541supp001.pdf]

INFORMED CONSENT FORM

(English Version)

Participant Information Page

**Study Title**	:	Effectiveness of ultrasound therapy for the treatment of carpal tunnel syndrome
**Principal Investigator**	:	Cunyi Fan
**Sponsor**	:	Shanghai Sixth People's Hospital

Dear participant:

You have been diagnosed with carpal tunnel syndrome, and will be invited to participate in the study named "Effectiveness of ultrasound therapy for the treatment of carpal tunnel syndrome". The study is conducted by the researchers themselves. Please read this informed consent carefully and make the decision whether to participate in this study or not. Participation in this study is entirely your choice. As a participant, you must give your written consent prior to joining the clinical study. When your doctor or researcher discusses informed consent with you, you can ask him or her to explain to you what you don't understand. We encourage you to discuss this thoroughly with your family and friends before making any decision to participate in this study. You have the right to refuse to participate in the study or withdraw from the study at any time without being penalized or losing your rights. If you are participating in another study, please inform your study doctor or investigator. The background, purpose, process and other important information of this study are as follows:

1. BACKGROUND

Carpal tunnel syndrome (CTS), the most common compression neuropathy, results from median nerve entrapment in the carpal tunnel, accounting for about 90% of all such disorders. The clinically confirmed CTS prevalence was 9.6% in the general population of China, with a yearly incidence rate of 2.76‰, and women is more susceptible than men. CTS has significant impact on daily life and ability to work, and causes great burden on social economy, with an annual associated cost estimated at $13 billion. Classically, CTS causes discomfort, paraesthesia and numbness in the median nerve distribution; and nocturnal symptoms are often clinically significant causing sleep disturbance. Patients can

be diagnosed by clinical history and physical examination; while electrophysiological methods will be additional for insufficient diagnosis by clinical findings and severe cases that need surgical management.

In general, the severity of CTS can be classified into mild, moderate and severe. Non-surgical interventions are suggested to be the first choice to treat mild and moderate CTS. To date, though the treatment method is vast; however, no successful and universally accepted regimen has been established. A consensus of multidisciplinary treatment guideline from the European HANDGUIDE Study suggests that "education" should be included as the first-line management approach, which has the advantages of low cost, high efficacy and non-invasiveness. In addition, "night splint (NS)" and "corticosteroid injection (CI)" are also recommended in guidelines of American Academy of Orthopaedic Surgeons (AAOS) and American Physical Therapy Association (APTA). One recent RCT published in Lancet compares both two methods, and finds that CI has superior clinical effectiveness at 6 weeks than NS, but no differences at 6 months; while CI may bring adverse events like thinning, lightening or darkening of the skin at the injection site, hot flushes and even more pain. Systematic reviews have also shown that the effects of other conservative treatments like acupuncture, exercise and mobilization interventions, laser, extracorporeal shockwave therapy and platelet-rich plasma injection still remain controversial or provide little to no benefit.

Ultrasound (US) is widely used for imaging purposes and regarded as an adjunct to physiotherapy. In the intensity range of 0.5-2.0 W/cm^2, US may have the potential to induce a variety of biophysical effects in tissues. US experiments on stimulation of nerve conduction and regeneration, and discoveries of its anti-inflammatory effects all support that US may promote recovery of nerve compression. An RCT published in BMJ showed more pronounced subjective symptoms and electroneurographic variables for US treatment than sham control in patients with mild to moderate CTS. However, to our best of knowledge, no study has compared the efficacy between NS and US in CTS treatment yet. Additionally, some studies have also reported the efficacy of US to be used as part of a multi-intervention approach, but with low grade of study design and data. Therefore, the role of US in CTS treatment still needs to be further explored by high-quality study.

2. STUDY PURPOSE

The purpose of the current three-arm, prospective, randomized, multicenter trial is to investigate the effectiveness of US in treatment for CTS, that is, NS+US (combined) versus NS versus US, on clinical and functional outcomes, including Boston Carpal Tunnel

Chen S, et al. BMJ Open 2022; 12:e057541. doi: 10.1136/bmjopen-2021-057541

Questionnaire (BCTQ).

3. STUDY PROCESS

(1) How many people will participate in the study?

About 162 people will participate in the study at 4 municipal tertiary hospitals: Shanghai Sixth People's Hospital (leader unit), Shanghai East Hospital (participating unit), Shanghai Tenth People's Hospital (participating unit) and Pudong New Area People′s Hospital of Shanghai (participating unit).

(2) What are the study procedures?

Before you are enrolled in the study, your medical history will be asked, and you will be screened for carpal tunnel syndrome by using criteria developed from a consensus survey by the UK Primary Care Rheumatology Society.

After determining that you are eligible to participate in the study based on inclusion and exclusion criteria, you will be collected and randomly assigned to treatment:

A. Characteristic features collection

You will be asked for your age, sex, body mass index, affected wrist (whether bilateral), dominant arm, lifestyle (smoking and alcohol use), and medical history. As well as relevant questions about symptoms duration and previous treatments (rehabilitation exercises, injections or others). Others like occupation, employment characteristics (full-time or part-time work, manual or non-manual labor), employment status (whether on sickness absence), professional activity characteristics, and sports activities will be also collected.

B. Clinical features collection

You will complete the following questionnaires, including Boston Carpal Tunnel Questionnaire (BCTQ) for wrist function and symptom, sleep questionnaire for interrupted sleep, EuroQol-5D (EQ-5D) for life quality and health status, Hospital Anxiety and Depression Scale (HADS) for anxiety and depression status, Work Limitations Questionnaire (WLQ)-25 for functional limitations at work, Global Rating of Change (GROC) for treatment success and recurrence rate, as well as physical function examination and various electrophysiology and ultrasound parameters.

C. Treatment by group

At the beginning, all participants will participate in an about 30-minute group educational presentation by a research assistant on the same day as the baseline assessment. This presentation will cover the pathophysiology, treatment options, posture and activity

modification principles of CTS. The above information will also be provided to participants in the form of education booklets, to encourage them to review at home. Habits changes include limited wrist movement and a reduction in strenuous work activities, and the use of ergonomically friendly work tools helps reduce median nerve pressure.

You will be randomly assigned to one of three groups, [US group] vs. [NS group] vs. [US+NS group]:

(a) If you are assigned in the [US group], you will receive pulsed therapeutic ultrasound (model 1:4, Shanghai, China) for 6 weeks at a frequency of 1 MHz and intensity of 1.0 W/cm^2 for 15 minutes per session, in daily 5 times a week for the first 2 weeks and twice a week for another 4 weeks, to the area over the carpal tunnel, referred to a published trial [BMJ. 1998;316:731-735].

(b) If you are allocated to the [NS group], you will receive a splint to wear at night for 6 weeks, referred to a published trial [Lancet. 2018;392:1423-1433]. The splint holds the wrist in a neutral position or slightly extended 20° from the neutral position to avoid wrist movement, which can increase pressure on the carpal tunnel. The choose of each splint will be based on the size of each of your hands and arms. You will be shown how to fit and remove the wrist splint according to a standardized trial protocol. Oral guidance from the clinician on how and when to use splints will encourage and reinforce compliance, which will be also supported by written information, detailed care, and splint fitting and use. You will be instructed to perform gentle range-of-motion exercises when removing the splint to prevent stiffness.

(c) If you are randomized to the [US+NS group], you will receive both US for 6 weeks as in the [US group] and NS for 6 weeks as in the [NS group].

We discourage additional treatments to that assigned (that is, not per protocol) during the intervention period, but we allowed the use of simple analgesics as needed. You will report all not per protocol treatments, such as drugs, in a diary.

D. Follow-up features collection

Follow-up data will be collected during your visits to the hospital at 6 weeks, 3 and 6 months, and one year after random assignment.

(3) How long will the study last?

This study will continue for 1 year from the time you receive treatment, and we will collect follow-up information from you at 6 weeks, 3 months, 6 months, and one year at your regular outpatient review.

You may drop out of the study at any time without losing any benefits to which you are entitled. However, if you decide to withdraw during the study, you are encouraged to

Chen S, *et al. BMJ Open* 2022; 12:e057541. doi: 10.1136/bmjopen-2021-057541

talk to your doctor first. If you experience a serious adverse event, or if your study doctor feels it is not in your best interest to continue in the study, he or she may decide to withdraw you from the study. The sponsor or regulatory agency may also terminate during the study period. However, your withdrawal will not affect your normal medical treatment and rights.

If you withdraw from the study for any reason, you may be asked about your participation in the study. You may also be asked for a medical examination and follow-up questionnaire if your doctor deems it necessary.

(4) Information and biological specimens collected during the study

Biological specimens are not involved in this study, and the information collected is basic characteristics features, preoperative and follow-up clinical features (see the study procedures for details).

All data obtained will be kept strict and stored electronically on a database with secured and restricted access. An encryption will be used for data transfer, with removal for any information able to identify individuals. Data will be only deidentified for analysis at the completion of this study.

4. RISKS AND BENEFITS

(1) What are the risks of participating in this study?

The risks you may incur by participating in this study are as follows. You should discuss these risks with your study doctor or, if you prefer, with your regular care provider.

US treatment may cause mild local swelling, spot-like bleeding, ecchymosis, enhanced local pain response, and local hyperesthesia or decrease. The occurrence of these reactions depends on the dose of treatment, the extent of the lesion, and the individual patient, and usually does not require special treatment. Severe adverse reactions can be treated locally, or prolong the interval of treatment, reduce the intensity of treatment. If the treatment does not improve or abnormal conditions occur, the treatment should be stopped and immediately go to the hospital.

NS treatment may cause skin allergy, wrist stiffness, et al. You will be instructed to do gentle range-of-motion exercises when removing the splint to prevent stiffness and reinforced adherence by verbal instruction.

If you experience any discomfort, new changes, or any unexpected conditions during the study period, whether or not related to the study, you should inform your doctor in a timely manner, and he/she will judge and administer appropriate medical treatment.

During the study period, you need to visit the hospital on time and do some

Chen S, *et al. BMJ Open* 2022; 12:e057541. doi: 10.1136/bmjopen-2021-057541

examinations, which will take up some of your time and may cause trouble or inconvenience to you.

(2) What are the benefits of participating in the study?

If you agree to participate in this study, you may receive direct medical benefits, such as accelerated relief of symptoms of CTS. You can also have a deeper understanding of diseases and so on. In addition, we hope that the information gained from your participation in this study will benefit you or other patients with similar conditions in the future.

5. ALTERNATIVE TREATMENT OPTIONS

In addition to participating in this study, you may receive the other treatments provided by your doctor: corticosteroid injection, acupuncture, exercise and mobilization interventions, laser, extracorporeal shockwave therapy and platelet-rich plasma injection, and surgery, etc.

Please discuss these and other possible options with your doctor.

In general, the severity of CTS can be classified into mild, moderate and severe. Non-surgical interventions are suggested to be the first choice to treat mild and moderate CTS. To date, though the treatment method is vast; however, no successful and universally accepted regimen has been established. A consensus of multidisciplinary treatment guideline from the European HANDGUIDE Study suggests that “education” should be included as the first-line management approach, which has the advantages of low cost, high efficacy and non-invasiveness. In addition, “night splint (NS)” and “corticosteroid injection (CI)” are also recommended in guidelines of American Academy of Orthopaedic Surgeons (AAOS) and American Physical Therapy Association (APTA). One recent RCT published in Lancet compares both two methods, and finds that CI has superior clinical effectiveness at 6 weeks than NS, but no differences at 6 months; while CI may bring adverse events like thinning, lightening or darkening of the skin at the injection site, hot flushes and even more pain. Systematic reviews have also shown that the effects of other conservative treatments like acupuncture, exercise and mobilization interventions, laser, extracorporeal shockwave therapy and platelet-rich plasma injection still remain controversial or provide little to no benefit.

6. USE OF RESEACH RESULTS AND CONFIDENTIALITY OF PERSONAL INFORMATION

Chen S, *et al. BMJ Open* 2022; 12:e057541. doi: 10.1136/bmjopen-2021-057541

Results conducted through this program may be published in medical journals with the understanding and assistance of you and other participants, but we will keep your study records confidential as required by law.

The personal information of study participants will be kept strictly confidential, and your personal information will not be disclosed unless required by relevant laws.

If necessary, government administrative departments, hospital ethics committees and other relevant researchers can access your data according to regulations.

7. RESEARCH EXPENSES AND RELATED COPENSATION

(1) Cost of drugs/instruments used in the study and related examinations

There are no potential additional costs for this study. Routine outpatient fees include registration, examination for CTS, oral non-steroidal anti-inflammatory drugs, etc. The expenses related to US and NS will be borne by our research group and funding. In addition, you will be solely responsible for the expenses incurred by you for any treatment other than this study, as well as for the routine treatment and examination required for any concurrent disease.

(2) Compensation for participation in the study

There are no additional compensation costs for this study.

(3) Compensation/compensation after damage

For participants who suffer damage related to this study, the sponsor Shanghai Sixth People's Hospital will bear the treatment cost and corresponding economic compensation in accordance with Chinese laws and regulations.

8. RIGHTS OF PARTICIPANTS AND RELEVANT MATTERS NEEDING ATTENTION

(1) Your rights

Your participation in the study is voluntary throughout the entire process.

If you decide not to participate in this study, it will not affect other treatments you should receive.

If you decide to participate, you will be asked to sign this written informed consent. You have the right to withdraw from the trial at any stage without discrimination or unfair treatment, and your medical treatment and rights will not be affected.

Chen S, *et al. BMJ Open* 2022; 12:e057541. doi: 10.1136/bmjopen-2021-057541

(2) Matters needing attention

As a subject, you are required to provide true information about your medical history and current medical condition;

Inform the study doctor of any discomfort observed during the study;

Do not take any restricted drugs, food, etc. as advised by your doctor;

Tell the study doctor if you have recently participated in or are currently participating in other studies.

During the intervention, we discouraged additional therapy (i.e., not according to the grouping protocol), but we permitted the use of analgesics when needed (only acetaminophen and NSAIDs).

For medications taken, the name, dose, frequency and duration will be recorded at all follow-up visits.

9. RELEVANT CONTACT INFORMATION

If there is any significant new information during the study that may affect your willingness to continue to participate, your doctor will inform you promptly. If you are interested in your own study data, or you would like to know the findings after this study, you may ask any questions about this study at any time and receive answers accordingly, Please contact doctor Shuai Chen at ***********.

Chen S, *et al. BMJ Open* 2022; 12:e057541. doi: 10.1136/bmjopen-2021-057541

Participant Signature Page

Informed Consent Statement:

I have been informed of the purpose, background, process, risks and benefits of this study. I have plenty of time and opportunity to ask questions, and I am satisfied with the answers.

I am also told who to contact when I have questions, want to report difficulties, concerns, suggestions for research, or want further information, or to help with research.

I have read this informed consent and agree to participate in this study.

I understand that I may choose not to participate in the study or withdraw from the study at any time during the study without any reason.

I already know that if I get worse, or if I have a serious adverse event, or if my study doctor decides it's not in my best interest to continue, he or she will decide to withdraw me from the study. The funder or regulatory agency may terminate during the study without my consent. If this happens, the doctor will inform me and the study doctor will discuss other options with me.

I will be provided with a copy of the informed consent which contains my signature and that of the investigator.

Participant Signature: _______________
Date: _______________
(NOTE: If participant has no capacity/limited capacity, legal representative signature and date will be required)

Legal Representative's Signature: _______________
Date: _______________

Investigator Signature: _______________
Date: _______________

Chen S, *et al. BMJ Open* 2022; 12:e057541. doi: 10.1136/bmjopen-2021-057541
