# Supplementary material for: Effectiveness of therapeutic ultrasound for the treatment of carpal tunnel syndrome (the USTINCTS trial): study protocol for a three-arm, prospective, multicentre, randomised controlled trial
Source: BMJ Open. 2022 Apr 13;12(4):e057541. doi: 10.1136/bmjopen-2021-057541 (PMC9014071; doi:10.1136/bmjopen-2021-057541)
Supplement: Author's manuscript [file bmjopen-2021-057541.draft_revisions.pdf]

BMJ Open is committed to open peer review. As part of this commitment we make the peer review history of every article we publish publicly available.

When an article is published we post the peer reviewers' comments and the authors' responses online. We also post the versions of the paper that were used during peer review. These are the versions that the peer review comments apply to.

The versions of the paper that follow are the versions that were submitted during the peer review process. They are not the versions of record or the final published versions. They should not be cited or distributed as the published version of this manuscript.

BMJ Open is an open access journal and the full, final, typeset and author-corrected version of record of the manuscript is available on our site with no access controls, subscription charges or pay-per-view fees (http://bmjopen.bmj.com).

If you have any questions on BMJ Open's open peer review process please email info.bmjopen@bmj.com

BMJ Open

Effectiveness of ultrasound therapy for the treatment of carpal tunnel syndrome (the USTINCTS trial): study protocol for a three-arm, prospective, multicenter, randomised controlled trial

Journal:	*BMJ Open*
Manuscript ID	bmjopen-2021-057541
Article Type:	Protocol
Date Submitted by the Author:	19-Sep-2021
Complete List of Authors:	Chen, Shuai; Shanghai Jiao Tong University Affiliated Sixth People's Hospital, Department of Orthopedics; Shanghai Engineering Research Center for Orthopaedic Material Innovation and Tissue Regeneration,<br>Sun, Ziyang; Shanghai Jiao Tong University Affiliated Sixth People's Hospital; Shanghai Engineering Research Center for Orthopaedic Material Innovation and Tissue Regeneration<br>Liu, Weixuan; Shanghai Jiao Tong University Affiliated Sixth People's Hospital, Department of Orthopaedics; Shanghai Engineering Research Center for Orthopaedic Material Innovation and Tissue Regeneration<br>Sun, Guixin; Shanghai East Hospital, Tongji University School of Medicine, Department of Orthopaedics<br>Liu, Junjian; Shanghai Tenth People's Hospital,<br>Wang, Jian; Pudong New Area People's Hospital, Department of Orthopedics<br>Wang, Wei; Shanghai Jiao Tong University Affiliated Sixth People's Hospital, Department of Orthopedics; Shanghai Engineering Research Center for Orthopaedic Material Innovation and Tissue Regeneration<br>Zheng, Yuanyi; Shanghai Jiao Tong University Affiliated Sixth People's Hospital, Department of Ultrasound in Medicine<br>Fan, Cunyi; Shanghai Jiao Tong University Affiliated Sixth People's Hospital, Department of Orthopedics; Shanghai Engineering Research Center for Orthopaedic Material Innovation and Tissue Regeneration
Keywords:	REHABILITATION MEDICINE, SPORTS MEDICINE, Hand & wrist < ORTHOPAEDIC & TRAUMA SURGERY, Orthopaedic sports trauma < ORTHOPAEDIC & TRAUMA SURGERY

SCHOLARONE™
Manuscripts

TITLE PAGE

Title

Effectiveness of ultrasound therapy for the treatment of carpal tunnel syndrome (the USTINCTS trial): study protocol for a three-arm, prospective, multicenter, randomised controlled trial

Running Title

study protocol of USTINCTS trial for carpal tunnel syndrome

Keywords

Carpal tunnel syndrome, randomised controlled trial, ultrasound therapy, night splint, Boston Carpal Tunnel Questionnaire

Word count

3958 words

Authors and information

Shuai Chen, MD [a, b, 1], Ziyang Sun, MD [a, b, 1], Weixuan Liu, MD [a, b, 1], Guixin Sun, MD, PhD [c], JunJian Liu, MD, PhD [d], Jian Wang, MD, PhD [e], Wei Wang, MD [a, b], Yuanyi Zheng, MD, PhD [f, *], Cunyi Fan, MD, PhD [a, b, *]

a Department of Orthopedics, Shanghai Jiao Tong University Affiliated Sixth People's Hospital, Shanghai, 200233, P. R. China

b Shanghai Engineering Research Center for Orthopaedic Material Innovation and Tissue Regeneration, Shanghai, 201306, P. R. China

c Department of Orthopaedics, Shanghai East Hospital, Tongji University School

For peer review only - http://bmjopen.bmj.com/site/about/guidelines.xhtml

of Medicine, Shanghai, 200120, P. R. China

d Department of Orthopedics, Shanghai Tenth People's Hospital, School of Medicine, Tongji University, Shanghai, 200092, P. R. China

e Department of Orthopaedics, Pudong New Area People's Hospital, Shanghai, 201299, P. R. China

f Department of Ultrasound in Medicine, Shanghai Jiao Tong University Affiliated Sixth People's Hospital, Shanghai, 200233, P. R. China

1 Z. Sun, S. Chen and W. Liu contribute equally to this work as co-first authors.

* C. Fan is corresponding author and Y. Zheng is co-corresponding author of this article.

Corresponding author (Cunyi Fan): ORCID: 0000-0002-7854-5233; E-mail: cyfan@sjtu.edu.cn; Affiliation: Department of Orthopedics, Shanghai Jiao Tong University Affiliated Sixth People's Hospital, Shanghai, 200233, P. R. China; Address: No. 600 Yishan Road, Shanghai, P. R. China

Co-corresponding author (Yuanyi Zheng): ORCID: 0000-0002-1328-0641; E-mail: zhengyuanyi@sjtu.edu.cn; Affiliation: Department of Ultrasound in Medicine, Shanghai Jiao Tong University Affiliated Sixth People's Hospital, Shanghai, 200233, P. R. China; Address: No. 600 Yishan Road, Shanghai, P. R. China

For peer review only

Author Contributions

CS and SZY are the primary investigators.

CS, SZY, LWX, ZYY, FCY participated in the development of the study design.

CS, SZY, LWX, SGX, LJJ, WJ, WW, ZYY, and FCY participated in the study conduct.

CS, SZY and LWX drafted the manuscript under FCY's supervision.

For peer review only - http://bmjopen.bmj.com/site/about/guidelines.xhtml

FCY contributed to applying for and gaining funding.

All authors contributed to the content and critical revision and approved the final draft of the manuscript.

Conflict of interests

The authors, their immediate families, and any research foundation with which they are affiliated have not received any financial payments or other benefits from any commercial entity related to the subject of this article.

The authors declare no competing financial interests.

Funding

This study will be supported by General Project of National Natural Science Foundation of China (8217090787); Shanghai Engineering Technology Research Center and Professional Technology Service Platform project of 2020 "Science and Technology Innovation Action Plan" of Shanghai (20DZ2254100); Municipal Hospital Clinical Skills and Innovation Capacity of Three-year Action Plan Program of Shanghai Shenkang Hospital Development Center (SHDC2020CR2039B, SHDC2020CR6019-002); Biomedical Technology Support Special Project of Shanghai "Science and Technology Innovation Action Plan" (20S31900300, 21S31902300); Clinical Research Center (CRC) of Shanghai University of Medicine and Health Sciences (20MC2020001).

Acknowledgments

We will appreciate the support from Base for Interdisciplinary Innovative Talent Training, Shanghai Jiao Tong University and Youth Science and Technology

For peer review only

For peer review only - http://bmjopen.bmj.com/site/about/guidelines.xhtml

Innovation Studio of Shanghai Jiao Tong University School of Medicine.

ETHICS

The Ethics Committee of the 4 clinical centers have approved this study. The Ethics Committee approval number of the leading clinical center (Shanghai Sixth People's Hospital) is 2021-152. The research registry number is ChiCTR2100050701 at http://www.chictr.org.cn. Data will be analyzed anonymously; all patients will approve the results of this study by oral consent. The oral consent approval will be documented in the patients' files. All clinical investigations will be conducted in accordance with the guidelines of the Declaration of Helsinki.

For peer review only

ABSTRACT

Introduction

There has no consensus on optimal management of carpal tunnel syndrome (CaTS), the most common compression neuropathy. Conservative therapy is generally accepted as first-line intervention. Ultrasound (US) therapy has been widely reported to be treatment beneficial in nerve regeneration and conduction, and further accelerate compression recovery. The purpose of this study is to investigate the effectiveness of US for CaTS treatment.

Methods and analysis

This study protocol entails a three-arm, prospective, multicenter, randomised controlled trial. 63 eligible participants diagnosed with CaTS will be assigned to either (1) US, (2) night splint or (3) combined group. Primary outcome is Symptom Severity Scale of Boston Carpal Tunnel Questionnaire (BCTQ-SSS). Secondary outcomes include Functional Status Scale of BCTQ, numerical rating scale for hand-wrist symptom intensity, shortened version of the Disabilities of the Arm, Shoulder and Hand for upper limb disability, sleep questionnaire for interrupted sleep, EuroQol-5D for general health, Hospital Anxiety and Depression Scale for mental status, Work Limitations Questionnaire-25 for functional limitations at work, Global Rating of Change for treatment success and recurrence rate, Likert scale for participant's satisfaction, physical examination, electrophysiological and ultrasound parameters. Intention-to-treat analyses will be used.

Ethics and dissemination

Ethics Committees of all clinical centers have approved this study. The leading center is Shanghai Sixth People's Hospital, whose approval number is 2021-152. New versions with appropriate amendments will be submitted to the committee for further

For peer review only - http://bmjopen.bmj.com/site/about/guidelines.xhtml

approval. Study results will be published in peer-reviewed journals and presented at local, national and international conferences.

Trial registration number

ChiCTR2100050701.

For peer review only

For peer review only - http://bmjopen.bmj.com/site/about/guidelines.xhtml

STRENGTHS AND LIMITATIONS OF THIS STUDY

- Ultrasound (US) as independent or adjunct therapy in treating carpal tunnel syndrome (CaTS).
- The first randomised controlled trial (RCT) to compared the efficacy between US and night splint in CaTS treatment.
- Multicenter RCT with blinded outcome assessor and statistician.
- Use of several patient-reported outcome measures as well as objective parameters.
- Participants and treating surgeons not blinded.

For peer review only

For peer review only - http://bmjopen.bmj.com/site/about/guidelines.xhtml

INTRODUCTION

Carpal tunnel syndrome (CaTS), the most common compression neuropathy, results from median nerve entrapment in the carpal tunnel, accounting for about 90% of all such disorders.[1,2] The clinically confirmed CaTS prevalence was 9.6% in the general population of China,[3] with a yearly incidence rate of 2.76‰, and women is more susceptible than men.[2,4] CaTS has significant impact on daily life and ability to work,[5] and causes great burden on social economy, with an annual associated costs estimated at $13 billion.[6] Classically, CaTS causes discomfort, paraesthesia and numbness in the median nerve distribution; and nocturnal symptoms are often clinically significant causing sleep disturbance.[7] Patients can be diagnosed by clinical history and physical examination; while electrophysiological methods will be additional for insufficient diagnosis by clinical findings and severe cases that need surgical management.[8]

In general, the severity of CaTS can be classified into mild, moderate and severe.[9] Non-surgical interventions are suggested to be the first choice to treat mild and moderate CaTS.[10] To date, though the treatment method is vast; however, no successful and universally accepted regimen has been established. A consensus of multidisciplinary treatment guideline from the European HANDGUIDE Study suggests that "education" should be included as the first-line management approach, which has the advantages of low cost, high efficacy and non-invasive.[7,11] In addition, "night splint (NS)" and "corticosteroid injection (CI)" are also recommended in guidelines of American Academy of Orthopaedic Surgeons (AAOS) and American Physical Therapy Association (APTA).[12,13] One recent RCT published in Lancet compares both two methods, and finds that CI has superior clinical effectiveness at 6 weeks than NS, but no differences at 6 months; while CI may bring adverse events like thinning, lightening

For peer review only

For peer review only - http://bmjopen.bmj.com/site/about/guidelines.xhtml

or darkening of the skin at the injection site, hot flushes and even more pain.[9] Systematic reviews have also shown that the effects of other conservative treatments like acupuncture,[14] exercise and mobilization interventions,[15] laser,[16] extracorporeal shockwave therapy[17] and platelet-rich plasma injection[18] still remain controversial or provide little to no benefit.

Ultrasound (US) is widely used for imaging purposes and regarded as an adjunct to physiotherapy. With an intensity range of 0.5-2.0 W/cm^2, US may have the potential to induce various biophysical effects within tissue.[19] Experiments of US on nerve conduction and stimulation of nerve regeneration,[20,21] and findings of its anti-inflammatory effects[22] all support that US might facilitate recovery for nerve compression. An RCT published in BMJ showed more pronounced subjective symptoms and electroneurographic variables for US treatment than sham control in patients with mild to moderate CaTS.[23] However, to our best of knowledge, no study has compared the efficacy between NS and US in CaTS treatment yet. Additionally, some studies have also reported the efficacy of US to be used as part of a multi-intervention approach, but with low grade of study design and data[24-29]. Therefore, the role of US in CaTS treatment still needs to be further explored by high-quality study.

Therefore, the purpose of the current three-arm, prospective, randomized, multicenter trial is to examine the effectiveness of US in treatment for CaTS, that is, NS+US versus NS versus US, on clinical and functional outcomes, including Boston Carpal Tunnel Questionnaire (BCTQ) in patients diagnosed with CaTS.

For peer review only - http://bmjopen.bmj.com/site/about/guidelines.xhtml

METHODS

Study design

The design of this study is a three-arm, prospective, multicenter, randomised controlled trial, that will enroll participants with a diagnosis of mild to moderate CaTS from 4 municipal tertiary hospitals (Shanghai Sixth People's Hospital, Shanghai Tenth People's Hospital, Shanghai East Hospital, and Pudong New Area People′s Hospital of Shanghai). This manuscript is written according to the SPIRIT guidelines.[30]

Participant and public involvement

This study was done without participant involvement. Participants were not invited to comment on the design and not consulted to develop patient-relevant outcomes. Participants will not be invited to contribute to the writing or editing of this manuscript for readability or accuracy. The resulting publications will be disseminated to public via mass media. Participants as a whole will be acknowledged in the end of our publications and presentations.

Participant recruitment

Figure 1 shows the participant flow chart throughout the study. Participants will be recruited over a period of 5 months, from the intake clinics of 4 principals of each sub-centers. Additionally, we will recruit participants through other physicians and healthcare professionals, via the hospital intranet, community and medical association newsletters, etc. Those interested will contact the research assist who will provide further information about the study objectives and procedures and will perform an initial eligibility screening interview by telephone.

Medical evaluation and enrolment procedure

Participants found to be eligible will be invited to attend a medical examination, to confirm the CaTS diagnosis and assess eligibility to participate in the research project.

For peer review only

For peer review only - http://bmjopen.bmj.com/site/about/guidelines.xhtml

Inclusion criteria

1. Age ≥18 years old;
2. Clinical diagnosis based on presenting symptoms, clinical history and physical tests, by using criteria developed from a consensus survey by the UK Primary Care Rheumatology Society;[31]
3. Mild to moderate CaTS,[9] with symptoms longer than 6 weeks duration; participants with bilateral CaTS will be designated their study hand based on the most severe symptoms;
    - Mild: intermittent paraesthesia in the distribution of the median nerve
    - Moderate: constant paraesthesia, and reversible numbness or pain of idiopathic nature
    - Severe: constant pain, numbness or sensory loss in the wrist and hand (specifically palm, index, or middle finger, or thumb), or thenar muscle atrophy
4. Able to read and write in simplified Chinese (Mainland), understand and complete the questionnaire, and should provide informed consent.

Exclusion criteria

- CaTS secondary to wrist deformity, trauma, mass, pregnancy, hypothyroidism, or inflammatory arthropathy;
- Treatment by NS, US or injection within the past 6 months, or previous carpal tunnel surgery;
- Previous surgery on the affected wrist (or study wrist if bilateral CaTS);
- Unable to tolerate the study interventions;
- Trauma to the affected upper limb requiring immobilization or surgery within the past 12 months;

For peer review only

For peer review only - http://bmjopen.bmj.com/site/about/guidelines.xhtml

- Inter-current illness including, poorly controlled thyroid disease or diabetes mellitus, vibration-induced neuropathy, inflammatory joint disease, osteoarthritis, suspected complex neurological and musculoskeletal conditions, et al.;
- Known abuse of drugs or alcohol;
- Allergy to any of the splint materials;
- Contraindications to US, including dermatological conditions, abnormal sensation in the affected arm, indwelling electrical pumps/pacemakers, epilepsy, pregnancy or breastfeeding, et al.

Following the medical evaluation, a research assistant will meet with the eligible participants and obtain written informed consent. Demographic variables will be reported before treatment (baseline) of all participants regarding age, sex, body mass index, affected elbow (whether bilateral), dominant arm, lifestyle (smoking and drinking), and previous medical history. Participants will also be asked relevant questions about duration of symptoms and previous treatments (rehabilitation exercises, injections or others). Others like occupation, employment characteristics (full-time or part-time work, manual or non-manual labor), employment status (whether on sickness absence), and professional activity characteristics (repetitive movements for >4 hours/day; wrist flexion for >2 hours/day and use of computer keyboard/ mouse [how many hours/day]) will be also collected.

Randomization and blinding

Participants will be randomized in three intervention groups (either US or NS or US+NS arm) in a ratio of 1:1:1, using a computer-generated randomized sequence with varying unknown block sizes (either 3 or 6) for all study centers, without stratification. A research assistant with no involvement in the clinical care and evaluations of participants will prepare sequentially numbered, opaque, sealed envelopes according to

For peer review only

For peer review only - http://bmjopen.bmj.com/site/about/guidelines.xhtml

the randomization lists, with security in place to ensure allocation data cannot be accessed or influenced by any person. At the appropriate time, this assistant will open the envelope and assure coordination of the therapeutic interventions.

The outcome assessor and statistician will be blinded to group allocation and not involved in treatment procedures.

Intervention

At the beginning, all participants will attend a 20-30 minutes group education presentation by the research assistant on the same day as their baseline assessment. This presentation will cover the pathophysiology, treatment options, posture and activity modification principles of CaTS. This information will also be provided in the form of education booklets, encouraging participants to review at home. Changes in habits include limitation of wrist movement and reduction of heavy work activities, and the use of ergonomically friendly work tools can be useful in reducing median nerve stress.[32,33]

Participants in the [US group] will receive continuous mode US (Shanghai, China) at a frequency of 1 MHz and intensity of 1.0 W/cm^2 for 15 minutes in 5 days per week for 6 weeks to the area over the carpal tunnel.

Participants allocated to the [NS group] will receive a splint to wear at night for 6 weeks. The splint immobilizes the wrist in a neutral or slightly extended position (20° from neutral) in order to avoid movement of the wrist, which increases carpal tunnel pressure.[34] Each splint will be fitted according to the size of the participant's hand and arm using standard splints of differing sizes. Participants will be shown how to fit and remove the wrist splint according to a standardized trial protocol. Adherence will be encouraged and reinforced by verbal instruction from the clinician on how and when to use the splint and this will be supported by written information, detailing care, fitting

For peer review only - http://bmjopen.bmj.com/site/about/guidelines.xhtml

and use of the splint. Participants will be instructed to perform gentle range-of-motion exercises when removing the splint to prevent stiffness.

Participants randomized to the [US+NS group] will receive both US for 6 weeks as in the [US group] and NS for 6 weeks as in the [NS group].

For participants with bilateral CTS, the non-study hand will be treated according to normal clinical protocols in use at the research site.

We discouraged additional treatments to that assigned (that is, not per protocol) during the intervention period, but we allowed the use of simple analgesics as needed. Participants reported all not per protocol treatments, such as drugs, in a diary.

Data management

Data will be collected during the participants' visits to the hospital at baseline, 3 weeks, 6 weeks and 3 months after random assignment (**Table 1**). In order to maximize participant compliance in follow-up completion, reminder emails and a telephone call by the research assistant will be programmed. Registered participants will be withdrawn from the study if: (1) participant withdraws his/her consent, and (2) exclusion criteria is discovered after registration. The reason and date of discontinuation will be recorded. Consent to use the data already collected prior to a participant's withdrawal will be included in the consent form.

Primary outcome measure

The primary outcome measure will be the difference in Symptom Severity Scale of the Boston Carpal Tunnel Questionnaire (BCTQ-SSS). The BCTQ is a disease-specific questionnaire referring to a typical 24-hour period in the past two weeks,[35] and has been shown to be highly reproducible, internally consistent, valid and responsive to clinical change in CaTS.[36] It consists of two different subscales: Symptom Severity Scale (11 items, about symptom severity) and Functional Status Scale (8 items, about

For peer review only - http://bmjopen.bmj.com/site/about/guidelines.xhtml

the degrees of difficulty on daily activities), both rated on a five-point scale, with final scores for each subscale result in mean scores between 1 and 5. The overall score is calculated as the mean of all 19 items. Higher scores represent more severe symptoms and functional impairment. We use a validated Chinese version[37] of the BCTQ in this study.

Secondary outcome

Secondary outcome measures will be the differences in Functional Status Scale of the Boston Carpal Tunnel Questionnaire (BCTQ-FSS), numerical rating scale (NRS)[38,39] for hand-wrist symptom intensity, shortened version of the Disabilities of the Arm, Shoulder and Hand (quick-DASH)[40] for upper limb disability, sleep questionnaire for interrupted sleep[41], EuroQol-5D (EQ-5D)[42] for life quality and health status, Hospital Anxiety and Depression Scale (HADS)[43] for anxiety and depression status, Work Limitations Questionnaire (WLQ)-25[44] for functional limitations at work, Global Rating of Change (GROC) for treatment success and recurrence rate, Likert scale for participant's satisfaction, physical function examination as well as various electrophysiology and ultrasound parameters.

■ Hand-wrist symptom intensity (pain severity and paresthesia/dysthesia)

The NRS will be used for symptom intensity evaluation, which is a 0-10 numerical rating scale. 0 point represents "no pain", and 10 points represents "extremely severe pain".[38,39] A minimum decrease of 1.3 points or 25% reduction in NRS is considered the minimal clinically important difference.[45,46]

■ Upper limb disability

The well-validated simplified Chinese (Mainland) version of Quick-DASH[47] will be used for elbow function evaluation, which consists of eleven questions scored on a 5-point scale similar to the DASH.[40] Total and individual module scores will be

For peer review only - http://bmjopen.bmj.com/site/about/guidelines.xhtml

calculated out of 100, with a higher score indicating a worse status. A minimal clinically important difference of 15.91 points has been reported.[48]

- Interrupted sleep

Sleep questionnaire will be used for sleep quality evaluation, which consists of 4 questions, asking about how many times during the last month participants have experienced.[41] Six response categories are available for each question, and are coded on a 0-5 scale in sequence: not at all, l-3, 4-7, 8-14, 15-21 and 22-31 days. All questions are equally weighted and summed. Higher scores represent more interrupted sleep.

- Life quality and health status

The EQ-5D is one of the widely validated generic health-related quality of life (HRQol) measures known as its simplicity.[42] It contains a five-dimension descriptive system (mobility, self-care, usual activities, pain/discomfort and anxiety/depression) and a VAS, ranging from 0 to 1, in which 1 represents perfect health. All the dimensions are grouped into three levels (no problem, some problem and extreme problem). We used a validated Chinese version[49,50] of the EQ-5D, which has been recommended by China Guidelines for Pharmacoeconomic Evaluations 2011 for a measure for HRQol and health utility.[51]

- Anxiety and depression status

HADS will be used to identify and quantify two of the most common psychological disorders - anxiety and depression.[43] There is evidence of increased levels of anxiety and depression in people with LET.[52] HADS is a 14-item scale independent of somatic symptoms, which consists of two 7-item subscales measuring depression and anxiety respectively. A 4-point scale (from 0 representing absence of symptoms, to 3 representing maximum symptomatology) is used. The total scores for each subscale range from 0 to 21, with higher scores indicating higher levels of disorder.

For peer review only - http://bmjopen.bmj.com/site/about/guidelines.xhtml

HADS has two cut offs for categorization: 0-7, "non-case"; 8-10, "possible or doubtful case"; 11-21, "probable or definite case".[53]

■ Functional limitations at work

In order to gather information that is complementary to the pain and disability scales, functional limitations at work will be measured with the WLQ-25. It contains 25 items arranged under four subscales addressing four dimensions of job demands, those are, time demands, physical demands, mental/interpersonal demands, and output demands.[44] A five-level ordinal response scale ranging from 0 (all of the time) to 4 (none of the time) with an additional sixth option (does not apply to my job) is used. The total scores range from 0-100 points, and a 13-point (out of 100) improvement for the summed score is established for clinically important differences.[54]

■ Treatment success and recurrence rate

Participants' treatment impression of change regarding their condition will be recorded on a 6-point Likert scale (from "completely recovered", "much improved", "somewhat improved", "same", "worse" to "much worse"). Success rates will be calculated by dichotomizing responses. Participants who report their overall condition as "completely recovered" or "much improved" since the beginning of the study will be counted as successes, while other responses will be counted as failures.[55,56] Recurrence will primarily be defined as occurring when a participant rates a success at 3 weeks and a failure at 6 weeks or 3 months on GROC.[55,56]

■ Participants' satisfaction

Similarly, participants' level of satisfaction on the evolution of their condition will be determined on a validated 5-point Likert scale ranging from "very satisfied", "quite satisfied", "no opinion", "not very satisfied" to "not at all satisfied".[57,58]

■ Physical function examination

For peer review only - http://bmjopen.bmj.com/site/about/guidelines.xhtml

The physical examinations will include measurement of 2-point discrimination (performed on the radial and ulnar aspects of each digit), grip strength with a dynamometer (CAMRY, City of Industry, CA, USA), and pinch strength with the pinch gauge (three trials for each hand). The affected side will be measured first and then the unaffected side. The measurement readings will be not revealed to the subjects until the completion of the test. The mean of three consecutive trials, separated by a 20s pause, will be calculated. Results will be presented as a ratio of values of the symptomatic side/ asymptomatic side×100.[59]

2 point discrimination testing was started with a distance of 4 mm and successively increased if necessary by 2 mm. Grip strength and 3 point pinch strength, measured with the Baseline dynamometer and pinch gauge (Chattanooga Group, Hixson, Tennessee, USA), respectively, were recorded (three trials for each hand).

■ Electrophysiological Study

Median nerve distal motor latency (DML), compound muscle action potential (CMAP), sensory nerve conduction velocity (SNCV) and sensory nerve action potential (SNAP) amplitudes will be recorded.[55,56] DML and CMAP were measured by placing surface electrodes on the abductor pollicis brevis muscle, and stimulation was applied 8 cm proximal to the active recording electrode. SNAP and SNCV were obtained by using a ring electrode, which was placed on the proximal and distal interphalangeal joints of the index finger. The sensory conduction study was stimulated antidromically 14 cm proximal to the active electrode. Motor study was performed by supramaximal stimulation while the amplitude was measured an average of 10 times for the sensory study. All measurements will be made 3 times, and the values obtained will be averaged for analysis.

■ US parameters

For peer review only - http://bmjopen.bmj.com/site/about/guidelines.xhtml

The cross-sectional area (CSA) was measured using an electronic caliper at the scaphoid-pisiform level.[38,39] The measurements will be made 3 times, and the values obtained will be averaged for analysis.

Adverse events

All adverse events, defined as any negative or unwanted reactions to intervention, will be recorded through the symptoms reported by the patients, and observations by a researcher at every visit. US treatment may cause mild local swelling, spot-like bleeding, ecchymosis, enhanced local pain response, and local hyperesthesia or decrease. NS treatment may cause skin allergy, wrist stiffness, et al. The participants will be instructed to do gentle range of motion exercises when removing the splint to prevent stiffness and reinforced adherence by verbal instruction.

Sample size calculation

Sample size and power calculation are based on the primary outcome of BCTQ score. All sample size calculations assume two-sided analysis with a power of 90% ($1-\beta=0.90$) at a significant level of $\alpha=0.05$. Based on previous trial, a standard deviation (SD) of 0.38-point on BCTQ-SSS score will be used.[60] To detect a minimum clinically significant difference of 0.8-point[58] (superiority margin) between US+NS and NS groups (assuming a true difference of 1.18-point[27,60]), a total of 18 participants in each group is required. Allowing for an up to 15% drop out rate, we aim to enroll at least 21 participants in each group to complete the study.

Analysis plan

Baseline characteristics will be summarized for the three treatment groups using appropriate descriptive statistics. Both primary and secondary analysis will be conducted blind to treatment allocation and analyzed on intention-to-treat (ITT)[61] approach with all randomized participants retaining their original randomized group.

For peer review only

For peer review only - http://bmjopen.bmj.com/site/about/guidelines.xhtml

Multiple imputation by chained equations will be used to address missing data caused by loss to follow-up and non-responses if these missing data are judged to be random.

The primary comparisons for BCTQ-SSS scores will be made using linear regression. In secondary analyses, repeated measures mixed model[62] will also be used to examine the associations between treatments and repeated outcome measures, with terms of treatment, time, trial center and corresponding baseline values as covariates (age, gender, body mass index, et al.). Linear regression will be used for numerical outcomes, and logistic/ordinal regression for any categorical outcomes.

Quality assurance/monitoring/management

A Manual of Operations and Procedures (MOP) and case report form will be developed as per protocol to standardize all procedures and staff training in areas such as patient recruitment, outcome measurement, data entry, management, analysis, and security, which also include the monitoring plans to assure patient protection and data integrity, thus facilitating consistency in protocol implementation and data collection. The investigators, physicians, research assistants, outcome assessors and statisticians are different people, and should receive Good Clinical Practice training. A trained project manager will visit each center for monitoring to ensure data quality and compliance with trial protocol.

All data obtained will be kept strict and stored electronically on a database with secured and restricted access. An encryption will be used for data transfer, with removal for any information able to identify individuals. Data will be only deidentified for analysis at the completion of this study.

Study duration

For peer review only - http://bmjopen.bmj.com/site/about/guidelines.xhtml

Recruitment of the trial will begin in the November of 2021 and 3-month follow-up for all participants is anticipated to be completed by June 2022. See **Table 1** for time points and recruitment progress.

Ethics and dissemination

The study has been approved by all 4 Medical Ethics Committees (the approval number of the leading clinical center [Shanghai Sixth People's Hospital] is 2021-152) and will be conducted according to the principle of the Declaration of Helsinki (64th, 2013). All requirements regarding the welfare, rights and privacy of participants are fulfilled. The potential risks of this clinical trial are considered to be minimal and are addressed in the protocol and consent forms. A written consent will be obtained by clinical practitioners from each participant. The trial was registered on www.chictr.org website (registration number ChiCTR2100050701). Data will be published in peer-reviewed journals and presented at conferences, both nationally and internationally.

For peer review only - http://bmjopen.bmj.com/site/about/guidelines.xhtml

DISCUSSION

CaTS is a highly prevalent compression neuropathy, which results in significant paraesthesia and numbness in the median nerve distribution, especially nocturnal symptoms causing sleep disturbance, causing great socioeconomic burden. Up till now, there is still no consensus on the optimal management, and nonoperative treatment is generally accepted as the first-line intervention for mild and moderate CaTS. Multiple methods have been studied and reviewed in the recent decades, however, the exact efficacy still remains controversial.

In an RCT published in BMJ for mild to moderate CaTS, active US treatment (1 MHz, 1.0 W/cm^2) was applied to the area over the carpal tunnel in the experimental group, and indistinguishable sham US treatment was applied in the control group.[23] At 6 months' follow up, satisfactory improvement or complete remission of symptoms was observed in 74% receiving active treatment, which is significantly higher than those receiving sham treatment (20%). As for electroneurography, DML and SNCV improved significantly with active treatment while remained unchanged with sham treatment. Hand grip and finger pinch strength in physical examination also improved significantly with active treatment. All results suggested satisfying effects from US treatment for CaTS.

US can also be used as part of a multi-intervention approach. Some studies have compared NS alone to NS combined with US in treatment of CaTS, while the effects were different, and the grades of study design and data were low. Dincer U et al.[26] found that the improvements in the combined group were statistically significantly better ($p=0.043$) than those in NS alone group in BCTQ-SSS, as well as BCTQ-FSS ($p<0.001$), and VAS for pain ($p<0.001$). Similar results were also reported by Baysal O et al.[63];

For peer review only - http://bmjopen.bmj.com/site/about/guidelines.xhtml

while Jothi KP et al.[27], Sim SE et al.[28] and Armagan O et al.[29] found US therapy may add no benefit to splinting in CaTS.

In this study, to the best of our knowledge, it is the first to compared the efficacy between US [US group] and NS [NS group] in CaTS treatment. What's more, the additional effects of US [US+NS group] in a multi-intervention approach will be compared with NS alone [NS group]. In clinic, US is less invasive, less expensive, safer and more portable than other nonoperative therapy like drug injections for compression neuropathy and, if proved to be effective, could be offered to selected patients as part of non-operative therapy.

There are some on-going clinical trials on CaTS treatment recent years,[64-67] and our prospective randomized study proposes to complement and add to this relevant and much needed scientific effort.

For peer review only - http://bmjopen.bmj.com/site/about/guidelines.xhtml

For peer review only

REFERENCES

1. Seiler JG, 3rd, Daruwalla JH, Payne SH, et al. Normal Palmar Anatomy and Variations That Impact Median Nerve Decompression. J Am Acad Orthop Surg. 2017;25(9):e194-e203.
2. Kleopa KA. In the Clinic. Carpal Tunnel Syndrome. Ann Intern Med. 2015;163-5:Itc1.
3. Feng B, Chen K, Zhu X, et al. Prevalence and risk factors of self-reported wrist and hand symptoms and clinically confirmed carpal tunnel syndrome among office workers in China: a cross-sectional study. BMC Public Health. 2021;21(1):57.
4. Latinovic R, Gulliford MC, Hughes RA. Incidence of common compressive neuropathies in primary care. J Neurol Neurosurg Psychiatry. 2006;77(2):263-5.
5. Jerosch-Herold C, Houghton J, Blake J, et al. Association of psychological distress, quality of life and costs with carpal tunnel syndrome severity: a cross-sectional analysis of the PALMS cohort. BMJ Open. 2017;7(11):e017732.
6. Werner RA, Franzblau A, Gell N. Randomized controlled trial of nocturnal splinting for active workers with symptoms of carpal tunnel syndrome. Arch Phys Med Rehabil. 2005;86(1):1-7.
7. Padua L, Coraci D, Erra C, et al. Carpal tunnel syndrome: clinical features, diagnosis, and management. Lancet Neurol. 2016;15(12):1273-84.
8. Graham B. The value added by electrodiagnostic testing in the diagnosis of carpal tunnel syndrome. J Bone Joint Surg Am. 2008;90(12):2587-93.
9. Chesterton LS, Blagojevic-Bucknall M, Burton C, et al. The clinical and cost-effectiveness of corticosteroid injection versus night splints for carpal tunnel syndrome (INSTINCTS trial): an open-label, parallel group, randomised controlled trial. Lancet. 2018;392(10156):1423-33.

For peer review only - http://bmjopen.bmj.com/site/about/guidelines.xhtml

523 10. Jiménez Del Barrio S, Estébanez de Miguel E, Bueno Gracia E, et al. Effects of diacutaneous fibrolysis in patients with mild to moderate symptomatic carpal tunnel syndrome: a randomized controlled trial. Clin Rehabil. 2018;32(12):1645-55.

526 11. Huisstede BM, Fridén J, Coert JH, et al. Carpal tunnel syndrome: hand surgeons, hand therapists, and physical medicine and rehabilitation physicians agree on a multidisciplinary treatment guideline—results from the European HANDGUIDE Study. Arch Phys Med Rehabil. 2014;95(12):2253-63.

530 12. Carpal Tunnel Syndrome: A Summary of Clinical Practice Guideline Recommendations-Using the Evidence to Guide Physical Therapist Practice. J Orthop Sports Phys Ther. 2019;49(5):359-60.

533 13. Graham B, Peljovich AE, Afra R, et al. The American Academy of Orthopaedic Surgeons Evidence-Based Clinical Practice Guideline on: Management of Carpal Tunnel Syndrome. J Bone Joint Surg Am. 2016;98(20):1750-4.

536 14. Choi GH, Wieland LS, Lee H, et al. Acupuncture and related interventions for the treatment of symptoms associated with carpal tunnel syndrome. Cochrane Database Syst Rev. 2018;12(12):Cd011215.

539 15. Page MJ, O'Connor D, Pitt V, et al. Exercise and mobilisation interventions for carpal tunnel syndrome. Cochrane Database Syst Rev. 2012(6):Cd009899.

541 16. Franke TP, Koes BW, Geelen SJ, et al. Do Patients With Carpal Tunnel Syndrome Benefit From Low-Level Laser Therapy? A Systematic Review of Randomized Controlled Trials. Arch Phys Med Rehabil. 2018;99(8):1650-9.e15.

544 17. Kim JC, Jung SH, Lee SU, et al. Effect of extracorporeal shockwave therapy on carpal tunnel syndrome: A systematic review and meta-analysis of randomized controlled trials. Medicine (Baltimore). 2019;98(33):e16870.

For peer review only - http://bmjopen.bmj.com/site/about/guidelines.xhtml

547 18. Catapano M, Catapano J, Borschel G, et al. Effectiveness of Platelet-Rich Plasma
548 Injections for Nonsurgical Management of Carpal Tunnel Syndrome: A Systematic
549 Review and Meta-analysis of Randomized Controlled Trials. Arch Phys Med
550 Rehabil. 2020;101(5):897-906.
551 19. O'Brien WD, Jr. Ultrasound-biophysics mechanisms. Prog Biophys Mol Biol.
552 2007;93-1(3):212-55.
553 20. Schuhfried O, Vukanovic D, Kollmann C, et al. Effects of Pulsed Ultrasound
554 Therapy on Sensory Nerve Conduction Parameters and the Pain Threshold
555 Perceptions in Humans. Pm r. 2017;9(8):781-6.
556 21. Ilham SJ, Chen L, Guo T, et al. In vitro single-unit recordings reveal increased
557 peripheral nerve conduction velocity by focused pulsed ultrasound. Biomed Phys
558 Eng Express. 2018;4(4).
559 22. de Oliveira Perrucini PD, Poli-Frederico RC, de Almeida Pires-Oliveira DA, et al.
560 Anti-Inflammatory and Healing Effects of Pulsed Ultrasound Therapy on
561 Fibroblasts. Am J Phys Med Rehabil 2020;99(1):19-25.
562 23. Ebenbichler GR, Resch KL, Nicolakis P, et al. Ultrasound treatment for treating the
563 carpal tunnel syndrome: randomised "sham" controlled trial. BMJ.
564 1998;316(7133):731-5.
565 24. Page MJ, O'Connor D, Pitt V, et al. Therapeutic ultrasound for carpal tunnel
566 syndrome. Cochrane Database Syst Rev. 2013;2013(3):Cd009601.
567 25. Huisstede BM, Hoogvliet P, Franke TP, et al. Carpal Tunnel Syndrome:
568 Effectiveness of Physical Therapy and Electrophysical Modalities. An Updated
569 Systematic Review of Randomized Controlled Trials. Arch Phys Med Rehabil.
570 2018;99(8):1623-34.e23.

For peer review only

For peer review only - http://bmjopen.bmj.com/site/about/guidelines.xhtml

26. Dincer U, Cakar E, Kiralp MZ, et al. The effectiveness of conservative treatments of carpal tunnel syndrome: splinting, ultrasound, and low-level laser therapies. Photomed Laser Surg. 2009;27(1):119-25.

27. Jothi KP, Bland JDP. Ultrasound therapy adds no benefit to splinting in carpal tunnel syndrome. Muscle Nerve. 2019;60(5):538-43.

28. Sim SE, Gunasagaran J, Goh KJ, et al. Short-term clinical outcome of orthosis alone vs combination of orthosis, nerve, and tendon gliding exercises and ultrasound therapy for treatment of carpal tunnel syndrome. J Hand Ther. 2019;32(4):411-6.

29. Armagan O, Bakilan F, Ozgen M, et al. Effects of placebo-controlled continuous and pulsed ultrasound treatments on carpal tunnel syndrome: a randomized trial. Clinics (Sao Paulo). 2014;69(8):524-8.

30. Chan AW, Tetzlaff JM, Gøtzsche PC, et al. SPIRIT 2013 explanation and elaboration: guidance for protocols of clinical trials. BMJ. 2013;346:e7586.

31. Burton C, Chesterton LS, Davenport G. Diagnosing and managing carpal tunnel syndrome in primary care. Br J Gen Pract. 2014;64(622):262-3.

32. O'Connor D, Page MJ, Marshall SC, et al. Ergonomic positioning or equipment for treating carpal tunnel syndrome. Cochrane Database Syst Rev. 2012;1(1):Cd009600.

33. Buchan S, Amirfeyz R. Cochrane corner: ergonomic positioning or equipment for treating carpal tunnel syndrome. J Hand Surg Eur Vol. 2013;38(5):580-1.

34. Weiss ND, Gordon L, Bloom T, et al. Position of the wrist associated with the lowest carpal-tunnel pressure: implications for splint design. J Bone Joint Surg Am. 1995;77(11):1695-9.

For peer review only

For peer review only - http://bmjopen.bmj.com/site/about/guidelines.xhtml

35. Levine DW, Simmons BP, Koris MJ, et al. A self-administered questionnaire for the assessment of severity of symptoms and functional status in carpal tunnel syndrome. J Bone Joint Surg Am. 1993;75(11):1585-92.

36. Leite JC, Jerosch-Herold C, Song F. A systematic review of the psychometric properties of the Boston Carpal Tunnel Questionnaire. BMC Musculoskelet Disord. 2006;7:78.

37. Lue YJ, Lu YM, Lin GT, et al. Validation of the Chinese version of the Boston Carpal Tunnel Questionnaire. J Occup Rehabil. 2014;24(1):139-45.

38. Wu YT, Ke MJ, Ho TY, et al. Randomized double-blinded clinical trial of 5% dextrose versus triamcinolone injection for carpal tunnel syndrome patients. Ann Neurol. 2018;84(4):601-10.

39. Wu YT, Ho TY, Chou YC, et al. Six-month Efficacy of Perineural Dextrose for Carpal Tunnel Syndrome: A Prospective, Randomized, Double-Blind, Controlled Trial. Mayo Clin Proc. 2017;92(8):1179-89.

40. Beaton DE, Wright JG, Katz JN. Development of the QuickDASH: comparison of three item-reduction approaches. J Bone Joint Surg Am. 2005;87(5):1038-46.

41. Jenkins CD, Stanton BA, Niemcryk SJ, et al. A scale for the estimation of sleep problems in clinical research. J Clin Epidemiol. 1988;41(4):313-21.

42. EuroQol--a new facility for the measurement of health-related quality of life. Health Policy. 1990;16(3):199-208.

43. Zigmond AS, Snaith RP. The hospital anxiety and depression scale. Acta Psychiatr Scand. 1983;67(6):361-70.

44. Lerner D, Amick BC 3rd, Rogers WH, et al. The Work Limitations Questionnaire. Med Care. 2001;39(1):72-85.

For peer review only - http://bmjopen.bmj.com/site/about/guidelines.xhtml

45. Spadoni GF, Stratford PW, Solomon PE, et al. The evaluation of change in pain intensity: a comparison of the P4 and single-item numeric pain rating scales. J Orthop Sports Phys Ther. 2004;34(4):187-93.

46. Bijur PE, Chang AK, Esses D, et al. Identifying the minimum clinically significant difference in acute pain in the elderly. Ann Emerg Med. 2010;56(5):517-21.e1.

47. Cao S, Zhou R, Zhou H, et al. Reliability and validity of Simplified Chinese version of Quick Disabilities of the Arm, Shoulder, and Hand (QuickDASH) questionnaire: cross-cultural adaptation and validation. Clin Rheumatol. 2019;38(11):3281-7.

48. Franchignoni F, Vercelli S, Giordano A, et al. Minimal clinically important difference of the disabilities of the arm, shoulder and hand outcome measure (DASH) and its shortened version (QuickDASH). J Orthop Sports Phys Ther. 2014;44(1):30-9.

49. Wu C, Gong Y, Wu J, et al. Chinese Version of the EQ-5D Preference Weights: Applicability in a Chinese General Population. PLoS One. 2016;11(10):e0164334.

50. Luo N, Liu G, Li M, et al. Estimating an EQ-5D-5L Value Set for China. Value Health. 2017;20(4):662-9.

51. Sun S, Chen J, Johannesson M, et al. Population health status in China: EQ-5D results, by age, sex and socio-economic status, from the National Health Services Survey 2008. Qual Life Res. 2011;20(3):309-20.

52. Alizadehkhaiyat O, Fisher AC, Kemp GJ, et al. Pain, functional disability, and psychologic status in tennis elbow. Clin J Pain. 2007;23(6):482-9.

53. Pallant JF, Bailey CM. Assessment of the structure of the Hospital Anxiety and Depression Scale in musculoskeletal patients. Health Qual Life Outcomes. 2005;3:82.

For peer review only - http://bmjopen.bmj.com/site/about/guidelines.xhtml

54. Roy JS, MacDermid JC, Amick BC 3rd, et al. Validity and responsiveness of presenteeism scales in chronic work-related upper-extremity disorders. Phys Ther. 2011;91(2):254-66.

55. Wang JC, Liao KK, Lin KP, et al. Efficacy of Combined Ultrasound-Guided Steroid Injection and Splinting in Patients With Carpal Tunnel Syndrome: A Randomized Controlled Trial. Arch Phys Med Rehabil 2017;98(5):947-56.

56. Hsu PC, Liao KK, Lin KP, et al. Comparison of Corticosteroid Injection Dosages in Mild to Moderate Idiopathic Carpal Tunnel Syndrome: A Randomized Controlled Trial. Arch Phys Med Rehabil. 2020;101(11):1857-64.

57. Hay E, Barlas P, Foster N, et al. Is acupuncture a useful adjunct to physiotherapy for older adults with knee pain?: the "acupuncture, physiotherapy and exercise" (APEX) study [ISRCTN88597683]. BMC Musculoskelet Disord. 2004;5:31.

58. Chesterton LS, Dziedzic KS, van der Windt DA, et al. The clinical and cost effectiveness of steroid injection compared with night splints for carpal tunnel syndrome: the INSTINCTS randomised clinical trial study protocol. BMC Musculoskelet Disord. 2016;17(1):415.

59. Smidt N, van der Windt DA, Assendelft WJ, et al. Interobserver reproducibility of the assessment of severity of complaints, grip strength, and pressure pain threshold in patients with lateral epicondylitis. Arch Phys Med Rehabil. 2002;83(8):1145-50.

60. Aktürk S, Büyükavcı R, Aslan Ö, et al. Comparison of splinting and Kinesio taping in the treatment of carpal tunnel syndrome: a prospective randomized study. Clin Rheumatol. 2018;37(9):2465-9.

61. Sedgwick P. Intention to treat analysis versus per protocol analysis of trial data. BMJ. 2015;350:h681.

For peer review only - http://bmjopen.bmj.com/site/about/guidelines.xhtml

62. Detry MA, Ma Y. Analyzing Repeated Measurements Using Mixed Models. JAMA. 2016;315(4):407-8.

63. Baysal O, Altay Z, Ozcan C, et al. Comparison of three conservative treatment protocols in carpal tunnel syndrome. Int J Clin Pract. 2006;60(7):820-8.

64. Atroshi I, Tadjerbashi K, McCabe SJ, et al. Treatment of carpal tunnel syndrome with wrist splinting: study protocol for a randomized placebo-controlled trial. Trials. 2019;20(1):531.

65. Chen CC, Wu YT, Su YC, et al. Efficacy of laser acupuncture for carpal tunnel syndrome: A study protocol for a prospective double-blind randomized controlled trial. Medicine (Baltimore). 2019;98(30):e16516.

66. Dimitrova A, Murchison C, Oken B. Local effects of acupuncture on the median and ulnar nerves in patients with carpal tunnel syndrome: a pilot mechanistic study protocol. Trials. 2019;20(1):8.

67. Mason W, Ryan D, Khan A, et al. Injection versus Decompression for Carpal Tunnel Syndrome-Pilot trial (INDICATE-P)-protocol for a randomised feasibility study. Pilot Feasibility Stud. 2017;3:20.

For peer review only

For peer review only - http://bmjopen.bmj.com/site/about/guidelines.xhtml

683 **Figure Legends**

684 **Figure 1** Participant flow chart

685

For peer review only - http://bmjopen.bmj.com/site/about/guidelines.xhtml

Table 1 Study evaluation procedures and timeline

**Study procedure**	**Medical evaluation**	**Enrolment visit**	**3 weeks**	**6 weeks**	**3 months**
Determine eligibility	√	√			
Obtain signed consent		√			
Obtain medical and demographic data		√			
Give instructions for Pain medication diary		√			
Outcome measures					
Boston Carpal Tunnel Questionnaire		√	√	√	√
Numerical rating scale for hand-wrist symptom intensity		√	√	√	√
Shortened version of the Disabilities of the Arm, Shoulder and Hand questionnaire		√	√	√	√
Interrupted sleep questionnaire		√	√	√	√
EuroQol-5D		√	√	√	√
Hospital Anxiety and Depression Scale		√	√	√	√
Work Limitations Questionnaire-25		√	√	√	√
Treatment success rate			√	√	√
Treatment recurrence rate				√	√

For peer review only

Participants' satisfaction		√	√	√
Physical examination	√	√	√	√
Electrophysiological and ultrasound parameters	√	√	√	√

For peer review only

For peer review only - http://bmjopen.bmj.com/site/about/guidelines.xhtml

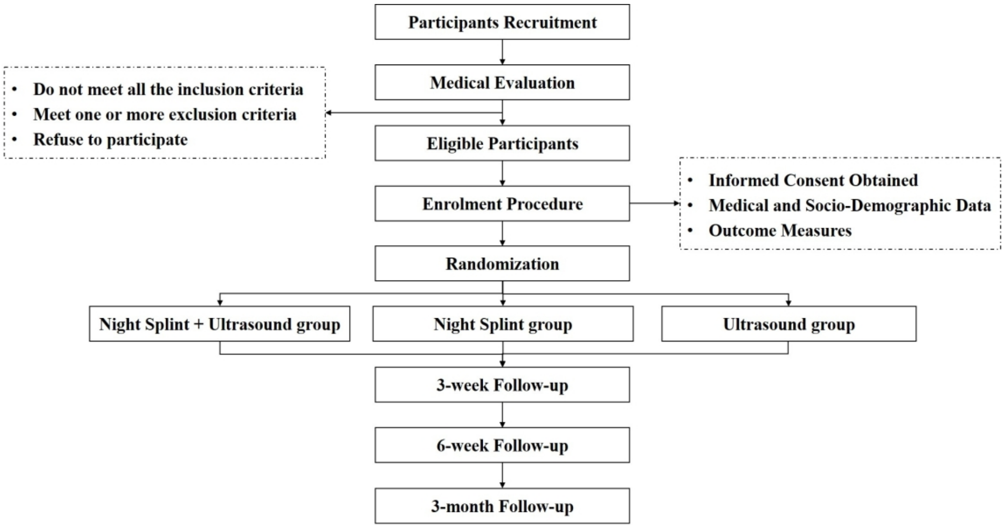

Participant flow chart

365x190mm (150 x 150 DPI)

Reporting checklist for protocol of a clinical trial.

Based on the SPIRIT guidelines.

Instructions to authors

Complete this checklist by entering the page numbers from your manuscript where readers will find each of the items listed below.

Your article may not currently address all the items on the checklist. Please modify your text to include the missing information. If you are certain that an item does not apply, please write "n/a" and provide a short explanation.

Upload your completed checklist as an extra file when you submit to a journal.

In your methods section, say that you used the SPIRITreporting guidelines, and cite them as:

Chan A-W, Tetzlaff JM, Gøtzsche PC, Altman DG, Mann H, Berlin J, Dickersin K, Hróbjartsson A, Schulz KF, Parulekar WR, Krleža-Jerić K, Laupacis A, Moher D. SPIRIT 2013 Explanation and Elaboration: Guidance for protocols of clinical trials. BMJ. 2013;346:e7586

		Reporting Item	Page Number
**Administrative information**			
Title	#1	Descriptive title identifying the study design, population, interventions, and, if applicable, trial acronym	1
Trial registration	#2a	Trial identifier and registry name. If not yet registered,	4/6

For peer review only

		name of intended registry	
Trial registration: data set	#2b	All items from the World Health Organization Trial Registration Data Set	4/6
Protocol version	#3	Date and version identifier	5
Funding	#4	Sources and types of financial, material, and other support	3
Roles and responsibilities: contributorship	#5a	Names, affiliations, and roles of protocol contributors	2
Roles and responsibilities: sponsor contact information	#5b	Name and contact information for the trial sponsor	2
Roles and responsibilities: sponsor and funder	#5c	Role of study sponsor and funders, if any, in study design; collection, management, analysis, and interpretation of data; writing of the report; and the decision to submit the report for publication, including whether they will have ultimate authority over any of these activities	2
Roles and responsibilities: committees	#5d	Composition, roles, and responsibilities of the coordinating centre, steering committee, endpoint adjudication committee, data management team, and other individuals or groups overseeing the trial, if applicable (see Item 21a for data monitoring committee)	2

Introduction

For peer review only

Background and rationale	#6a	Description of research question and justification for undertaking the trial, including summary of relevant studies (published and unpublished) examining benefits and harms for each intervention	8-9
Background and rationale: choice of comparators	#6b	Explanation for choice of comparators	8-9
Objectives	#7	Specific objectives or hypotheses	10
Trial design	#8	Description of trial design including type of trial (eg, parallel group, crossover, factorial, single group), allocation ratio, and framework (eg, superiority, equivalence, non-inferiority, exploratory)	10
**Methods: Participants, interventions, and outcomes**			
Study setting	#9	Description of study settings (eg, community clinic, academic hospital) and list of countries where data will be collected. Reference to where list of study sites can be obtained	10
Eligibility criteria	#10	Inclusion and exclusion criteria for participants. If applicable, eligibility criteria for study centres and individuals who will perform the interventions (eg,	11-12

For peer review only

		surgeons, psychotherapists)	
Interventions: description	#11a	Interventions for each group with sufficient detail to allow replication, including how and when they will be administered	13-14
Interventions: modifications	#11b	Criteria for discontinuing or modifying allocated interventions for a given trial participant (eg, drug dose change in response to harms, participant request, or improving / worsening disease)	13-14
Interventions: adherance	#11c	Strategies to improve adherence to intervention protocols, and any procedures for monitoring adherence (eg, drug tablet return; laboratory tests)	13-14
Interventions: concomitant care	#11d	Relevant concomitant care and interventions that are permitted or prohibited during the trial	13-14
Outcomes	#12	Primary, secondary, and other outcomes, including the specific measurement variable (eg, systolic blood pressure), analysis metric (eg, change from baseline, final value, time to event), method of aggregation (eg, median, proportion), and time point for each outcome. Explanation of the clinical relevance of chosen efficacy and harm outcomes is strongly recommended	14-19
Participant timeline	#13	Time schedule of enrolment, interventions (including any run-ins and washouts), assessments, and visits for participants. A schematic diagram is highly recommended (see Figure)	20-21

For peer review only

Sample size	#14	Estimated number of participants needed to achieve study objectives and how it was determined, including clinical and statistical assumptions supporting any sample size calculations	19
Recruitment	#15	Strategies for achieving adequate participant enrolment to reach target sample size	10-11
**Methods: Assignment of interventions (for controlled trials)**			
Allocation: sequence generation	#16a	Method of generating the allocation sequence (eg, computer-generated random numbers), and list of any factors for stratification. To reduce predictability of a random sequence, details of any planned restriction (eg, blocking) should be provided in a separate document that is unavailable to those who enrol participants or assign interventions	12-13
Allocation concealment mechanism	#16b	Mechanism of implementing the allocation sequence (eg, central telephone; sequentially numbered, opaque, sealed envelopes), describing any steps to conceal the sequence until interventions are assigned	12-13
Allocation: implementation	#16c	Who will generate the allocation sequence, who will enrol participants, and who will assign participants to interventions	12-13

For peer review only

Blinding (masking)	#17a	Who will be blinded after assignment to interventions (eg, trial participants, care providers, outcome assessors, data analysts), and how	12-13
Blinding (masking): emergency unblinding	#17b	If blinded, circumstances under which unblinding is permissible, and procedure for revealing a participant's allocated intervention during the trial	12-13
**Methods: Data collection, management, and analysis**			
Data collection plan	#18a	Plans for assessment and collection of outcome, baseline, and other trial data, including any related processes to promote data quality (eg, duplicate measurements, training of assessors) and a description of study instruments (eg, questionnaires, laboratory tests) along with their reliability and validity, if known. Reference to where data collection forms can be found, if not in the protocol	14, 19-21
Data collection plan: retention	#18b	Plans to promote participant retention and complete follow-up, including list of any outcome data to be collected for participants who discontinue or deviate from intervention protocols	14, 19-21
Data management	#19	Plans for data entry, coding, security, and storage, including any related processes to promote data quality (eg, double data entry; range checks for data values).	14, 19-21

For peer review only

		Reference to where details of data management procedures can be found, if not in the protocol	
Statistics: outcomes	#20a	Statistical methods for analysing primary and secondary outcomes. Reference to where other details of the statistical analysis plan can be found, if not in the protocol	19-20
Statistics: additional analyses	#20b	Methods for any additional analyses (eg, subgroup and adjusted analyses)	19-20
Statistics: analysis population and missing data	#20c	Definition of analysis population relating to protocol non-adherence (eg, as randomised analysis), and any statistical methods to handle missing data (eg, multiple imputation)	19-20
**Methods: Monitoring**			
Data monitoring: formal committee	#21a	Composition of data monitoring committee (DMC); summary of its role and reporting structure; statement of whether it is independent from the sponsor and competing interests; and reference to where further details about its charter can be found, if not in the protocol. Alternatively, an explanation of why a DMC is not needed	14, 19-21
Data monitoring: interim analysis	#21b	Description of any interim analyses and stopping guidelines, including who will have access to these interim results and make the final decision to terminate the trial	14, 19-21
Harms	#22	Plans for collecting, assessing, reporting, and managing solicited and spontaneously reported adverse events and other unintended effects of trial interventions or trial	19

For peer review only

		conduct	
Auditing	#23	Frequency and procedures for auditing trial conduct, if any, and whether the process will be independent from investigators and the sponsor	19
**Ethics and dissemination**			
Research ethics approval	#24	Plans for seeking research ethics committee / institutional review board (REC / IRB) approval	21
Protocol amendments	#25	Plans for communicating important protocol modifications (eg, changes to eligibility criteria, outcomes, analyses) to relevant parties (eg, investigators, REC / IRBs, trial participants, trial registries, journals, regulators)	21
Consent or assent	#26a	Who will obtain informed consent or assent from potential trial participants or authorised surrogates, and how (see Item 32)	21
Consent or assent: ancillary studies	#26b	Additional consent provisions for collection and use of participant data and biological specimens in ancillary studies, if applicable	21
Confidentiality	#27	How personal information about potential and enrolled participants will be collected, shared, and maintained in order to protect confidentiality before, during, and after the trial	21
Declaration of	#28	Financial and other competing interests for principal	21

For peer review only

interests		investigators for the overall trial and each study site	
Data access	#29	Statement of who will have access to the final trial dataset, and disclosure of contractual agreements that limit such access for investigators	14, 19-21
Ancillary and post trial care	#30	Provisions, if any, for ancillary and post-trial care, and for compensation to those who suffer harm from trial participation	19-21
Dissemination policy: trial results	#31a	Plans for investigators and sponsor to communicate trial results to participants, healthcare professionals, the public, and other relevant groups (eg, via publication, reporting in results databases, or other data sharing arrangements), including any publication restrictions	19-21
Dissemination policy: authorship	#31b	Authorship eligibility guidelines and any intended use of professional writers	19-21
Dissemination policy: reproducible research	#31c	Plans, if any, for granting public access to the full protocol, participant-level dataset, and statistical code	19-21
**Appendices**			
Informed consent materials	#32	Model consent form and other related documentation given to participants and authorised surrogates	/
Biological specimens	#33	Plans for collection, laboratory evaluation, and storage of biological specimens for genetic or molecular analysis in the current trial and for future use in ancillary studies, if applicable	/

For peer review only

None The SPIRIT Explanation and Elaboration paper is distributed under the terms of the Creative Commons Attribution License CC-BY-NC. This checklist can be completed online using https://www.goodreports.org/, a tool made by the EQUATOR Network in collaboration with Penelope.ai

For peer review only

BMJ Open

Effectiveness of ultrasound therapy for the treatment of carpal tunnel syndrome (the USTINCTS trial): study protocol for a three-arm, prospective, multicenter, randomised controlled trial

Journal:	*BMJ Open*
Manuscript ID	bmjopen-2021-057541.R1
Article Type:	Protocol
Date Submitted by the Author:	19-Jan-2022
Complete List of Authors:	Chen, Shuai; Shanghai Jiao Tong University Affiliated Sixth People's Hospital, Department of Orthopedics; Shanghai Engineering Research Center for Orthopaedic Material Innovation and Tissue Regeneration,<br>Sun, Ziyang; Shanghai Jiao Tong University Affiliated Sixth People's Hospital, Department of Orthopedics; Shanghai Engineering Research Center for Orthopaedic Material Innovation and Tissue Regeneration<br>Liu, Weixuan; Shanghai Jiao Tong University Affiliated Sixth People's Hospital, Department of Orthopedics; Shanghai Engineering Research Center for Orthopaedic Material Innovation and Tissue Regeneration<br>Sun, Guixin; Shanghai East Hospital, Tongji University School of Medicine, Department of Orthopaedics<br>Liu, Junjian; Shanghai Tenth People's Hospital,<br>Wang, Jian; Pudong New Area People's Hospital, Department of Orthopedics<br>Wang, Wei; Shanghai Jiao Tong University Affiliated Sixth People's Hospital, Department of Orthopedics; Shanghai Engineering Research Center for Orthopaedic Material Innovation and Tissue Regeneration<br>Zheng, Yuanyi; Shanghai Jiao Tong University Affiliated Sixth People's Hospital, Department of Ultrasound in Medicine<br>Fan, Cunyi; Shanghai Jiao Tong University Affiliated Sixth People's Hospital, Department of Orthopedics; Shanghai Engineering Research Center for Orthopaedic Material Innovation and Tissue Regeneration
**Primary Subject Heading**:	Sports and exercise medicine
Secondary Subject Heading:	Rehabilitation medicine
Keywords:	REHABILITATION MEDICINE, SPORTS MEDICINE, Hand & wrist < ORTHOPAEDIC & TRAUMA SURGERY, Orthopaedic sports trauma < ORTHOPAEDIC & TRAUMA SURGERY

SCHOLARONE™
Manuscripts

TITLE PAGE

Title

Effectiveness of ultrasound therapy for the treatment of carpal tunnel syndrome (the USTINCTS trial): study protocol for a three-arm, prospective, multicenter, randomised controlled trial

Running Title

study protocol of USTINCTS trial for carpal tunnel syndrome

Keywords

Carpal tunnel syndrome, randomised controlled trial, ultrasound therapy, night splint, Boston Carpal Tunnel Questionnaire

Word count

3958 words

Authors and information

Shuai Chen, MD [a, b, 1], Ziyang Sun, MD [a, b, 1], Weixuan Liu, MD [a, b, 1], Guixin Sun, MD, PhD [c], JunJian Liu, MD, PhD [d], Jian Wang, MD, PhD [e], Wei Wang, MD [a, b], Yuanyi Zheng, MD, PhD [f, *], Cunyi Fan, MD, PhD [a, b, *]

a Department of Orthopedics, Shanghai Jiao Tong University Affiliated Sixth People's Hospital, Shanghai, 200233, P. R. China

b Shanghai Engineering Research Center for Orthopaedic Material Innovation and Tissue Regeneration, Shanghai, 201306, P. R. China

c Department of Orthopaedics, Shanghai East Hospital, Tongji University School

For peer review only - http://bmjopen.bmj.com/site/about/guidelines.xhtml

of Medicine, Shanghai, 200120, P. R. China

d Department of Orthopedics, Shanghai Tenth People's Hospital, School of Medicine, Tongji University, Shanghai, 200092, P. R. China

e Department of Orthopaedics, Pudong New Area People's Hospital, Shanghai, 201299, P. R. China

f Department of Ultrasound in Medicine, Shanghai Jiao Tong University Affiliated Sixth People's Hospital, Shanghai, 200233, P. R. China

1 S. Chen, Z. Sun and W. Liu contribute equally to this work as co-first authors.

[*] C. Fan is corresponding author and Y. Zheng is co-corresponding author of this article.

Corresponding author (Cunyi Fan): ORCID: 0000-0002-7854-5233; E-mail: cyfan@sjtu.edu.cn; Affiliation: Department of Orthopedics, Shanghai Jiao Tong University Affiliated Sixth People's Hospital, Shanghai, 200233, P. R. China; Address: No. 600 Yishan Road, Shanghai, P. R. China

Co-corresponding author (Yuanyi Zheng): ORCID: 0000-0002-1328-0641; E-mail: zhengyuanyi@sjtu.edu.cn; Affiliation: Department of Ultrasound in Medicine, Shanghai Jiao Tong University Affiliated Sixth People's Hospital, Shanghai, 200233, P. R. China; Address: No. 600 Yishan Road, Shanghai, P. R. China

Author Contributions

CS and SZY are the primary investigators.

CS, SZY, LWX, ZYY, FCY participated in the development of the study design.

CS, SZY, LWX, SGX, LJJ, WJ, WW, ZYY, and FCY participated in the study conduct.

CS, SZY and LWX drafted the manuscript under FCY's supervision.

For peer review only - http://bmjopen.bmj.com/site/about/guidelines.xhtml

FCY contributed to applying for and gaining funding.

All authors contributed to the content and critical revision and approved the final draft of the manuscript.

Conflict of interests

The authors, their immediate families, and any research foundation with which they are affiliated have not received any financial payments or other benefits from any commercial entity related to the subject of this article.

The authors declare no competing financial interests.

Funding

This study will be supported by General Project of National Natural Science Foundation of China (8217090787); Shanghai Engineering Technology Research Center and Professional Technology Service Platform project of 2020 "Science and Technology Innovation Action Plan" of Shanghai (20DZ2254100); Municipal Hospital Clinical Skills and Innovation Capacity of Three-year Action Plan Program of Shanghai Shenkang Hospital Development Center (SHDC2020CR2039B, SHDC2020CR6019-002); Biomedical Technology Support Special Project of Shanghai "Science and Technology Innovation Action Plan" (20S31900300, 21S31902300); Clinical Research Center (CRC) of Shanghai University of Medicine and Health Sciences (20MC2020001).

Acknowledgments

We will appreciate the support from Base for Interdisciplinary Innovative Talent Training, Shanghai Jiao Tong University and Youth Science and Technology

For peer review only - http://bmjopen.bmj.com/site/about/guidelines.xhtml

Innovation Studio of Shanghai Jiao Tong University School of Medicine.

ETHICS

The study has been approved by all 4 Medical Ethics Committees, those are, Ethics Committee of Shanghai Sixth People’s Hospital (the leading clinical center, approval No. 2021-152), Ethics Committee of Shanghai East Hospital (EC.D(BG).016.03.1-2021-095), Ethics Committee of Shanghai Tenth People’s Hospital (SHSY-IEC-4.1/21-194/01), and Ethics Committee of Pudong New Area People's Hospital (IRBY2021-006). The research registry number is ChiCTR2100050701 at http://www.chictr.org.cn. Data will be analyzed anonymously; all patients will approve the results of this study by written consent. The written consent approval will be documented in the patients’ files. All clinical investigations will be conducted in accordance with the guidelines of the Declaration of Helsinki.

For peer review only

For peer review only - http://bmjopen.bmj.com/site/about/guidelines.xhtml

ABSTRACT

Introduction

There has no consensus on optimal management of carpal tunnel syndrome (CaTS), the most common compression neuropathy. Conservative therapy is generally accepted as first-line intervention. Ultrasound (US) therapy has been widely reported to be treatment beneficial in nerve regeneration and conduction, and further accelerate compression recovery. The purpose of this study is to investigate the effectiveness of US for CaTS treatment.

Methods and analysis

This study protocol entails a three-arm, prospective, multicenter, randomised controlled trial. 162 eligible adult participants diagnosed with mild to moderate CaTS by using criteria developed from a consensus survey by the UK Primary Care Rheumatology Society will be assigned to either (1) US, (2) night splint (NS) or (3) US+NS (combined) group. Primary outcome will be difference in Symptom Severity Scale of Boston Carpal Tunnel Questionnaire (BCTQ-SSS) at 6-week between NS and US+NS groups. Secondary outcomes include Functional Status Scale of BCTQ, numerical rating scale for hand-wrist symptom intensity, shortened version of the Disabilities of the Arm, Shoulder and Hand for upper limb disability, sleep questionnaire for interrupted sleep, EuroQol-5D for general health, Hospital Anxiety and Depression Scale for mental status, Work Limitations Questionnaire-25 for functional limitations at work, Global Rating of Change for treatment success and recurrence rate, Likert scale for participant's satisfaction, physical examination, electrophysiological and ultrasound parameters. Intention-to-treat analyses will be used.

Ethics and dissemination

For peer review only

For peer review only - http://bmjopen.bmj.com/site/about/guidelines.xhtml

Ethics Committees of all clinical centers have approved this study. The leading center is Shanghai Sixth People's Hospital, whose approval number is 2021-152. New versions with appropriate amendments will be submitted to the committee for further approval. Final results will be published in peer-reviewed journals and presented at local, national and international conferences.

Trial registration number

ChiCTR2100050701.

For peer review only

For peer review only - http://bmjopen.bmj.com/site/about/guidelines.xhtml

STRENGTHS AND LIMITATIONS OF THIS STUDY

- Ultrasound (US) as independent or adjunct therapy in treating carpal tunnel syndrome (CaTS).
- The first randomised controlled trial (RCT) to compared the efficacy between US and night splint in CaTS treatment.
- Multicenter RCT with blinded outcome assessor and statistician.
- Use of several patient-reported outcome measures as well as objective parameters.
- Participants and treating surgeons not blinded.

For peer review only - http://bmjopen.bmj.com/site/about/guidelines.xhtml

INTRODUCTION

Carpal tunnel syndrome (CaTS), the most common compression neuropathy, results from median nerve entrapment in the carpal tunnel, accounting for about 90% of all such disorders.[1,2] The clinically confirmed CaTS prevalence was 9.6% in the general population of China,[3] with a yearly incidence rate of 2.76‰, and women is more susceptible than men.[2,4] CaTS has significant impact on daily life and ability to work,[5] and causes great burden on social economy, with an annual associated costs estimated at $13 billion.[6] Classically, CaTS causes discomfort, paraesthesia and numbness in the median nerve distribution; and nocturnal symptoms are often clinically significant causing sleep disturbance.[7] Patients can be diagnosed by clinical history and physical examination; while electrophysiological methods will be additional for insufficient diagnosis by clinical findings and severe cases that need surgical management.[8]

In general, the severity of CaTS can be classified into mild, moderate and severe.[9] Non-surgical interventions are suggested to be the first choice to treat mild and moderate CaTS.[10] To date, though the treatment method is vast; however, no successful and universally accepted regimen has been established. A consensus of multidisciplinary treatment guideline from the European HANDGUIDE Study suggests that "education" should be included as the first-line management approach, which has the advantages of low cost, high efficacy and non-invasive.[7,11] In addition, "night splint (NS)" and "corticosteroid injection (CI)" are also recommended in guidelines of American Academy of Orthopaedic Surgeons (AAOS) and American Physical Therapy Association (APTA).[12,13] One recent RCT published in Lancet compares both two methods, and finds that CI has superior clinical effectiveness at 6 weeks than NS, but no differences at 6 months; while CI may bring adverse events

For peer review only

For peer review only - http://bmjopen.bmj.com/site/about/guidelines.xhtml

like thinning, lightening or darkening of the skin at the injection site, hot flushes and even more pain.[9] Systematic reviews have also shown that the effects of other conservative treatments like acupuncture,[14] exercise and mobilization interventions,[15] laser,[16] extracorporeal shockwave therapy[17] and platelet-rich plasma injection[18] still remain controversial or provide little to no benefit.

Ultrasound (US) is widely used for imaging purposes and regarded as an adjunct to physiotherapy. In the intensity range of 0.5-2.0 W/cm^2, US may have the potential to induce a variety of biophysical effects in tissues.[19] US experiments on stimulation of nerve conduction and regeneration,[20,21] and discoveries of its anti-inflammatory effects[22] all support that US may promote recovery of nerve compression. An RCT published in BMJ showed more pronounced subjective symptoms and electroneurographic variables for US treatment than sham control in patients with mild to moderate CaTS.[23] However, to our best of knowledge, no study has compared the efficacy between NS and US in CaTS treatment yet. Additionally, some studies have also reported the efficacy of US to be used as part of a multi-intervention approach, but with low grade of study design and data[24-29]. Therefore, the role of US in CaTS treatment still needs to be further explored by high-quality study.

Therefore, the purpose of the current three-arm, prospective, randomized, multicenter trial is to examine the effectiveness of US in treatment for CaTS, that is, NS+US (combined) versus NS versus US, on clinical and functional outcomes, including Boston Carpal Tunnel Questionnaire (BCTQ) in patients diagnosed with CaTS.

For peer review only - http://bmjopen.bmj.com/site/about/guidelines.xhtml

METHODS

Study design

This study is a three-arm, prospective, multicenter, randomized controlled trial that will recruit participants from 4 municipal tertiary hospitals with a diagnosis of mild to moderate CaTS. The multi-centres are Shanghai Sixth People's Hospital, Shanghai Tenth People's Hospital, Shanghai East Hospital, and Pudong New Area People′s Hospital of Shanghai, respectively. This manuscript is written according to the SPIRIT guidelines.[30]

Participant and public involvement

There was no participant involvement in this study. Participants were not invited to make comments or suggestions on the protocol, were not consulted about the selection of patient-relevant evaluation outcomes, or were invited to participate in writing or editing the manuscript to improve readability or accuracy. The final findings will be disseminated to the public through mass media. Our published papers and conference presentations also acknowledge all participants as a whole.

Participant recruitment

Figure 1 shows the participant flow chart throughout the study. Participants will be recruited over a 5-month period from outpatient clinics of 4 principals in each sub-centre. In addition, recruitment can also be through other doctors and healthcare professionals. Interested participants can contact the research assistant, who will provide more information about the study purpose and protocol, and conduct an initial eligibility screening by phone.

Medical evaluation and enrolment procedure

Eligible participants will be invited to participate in a physical examination to confirm CaTS diagnosis and assess eligibility to participate in the program.

For peer review only - http://bmjopen.bmj.com/site/about/guidelines.xhtml

Inclusion criteria

- Age ≥18 years old;
- Clinical diagnosis on the basis of clinical symptoms, history and physical examination, using criteria developed from a consensus survey by the UK Primary Care Rheumatology Society;[31]
- Mild to moderate CaTS,[9] with symptoms longer than 6 weeks duration; participants with bilateral CaTS will be designated their study hand based on the most severe symptoms;
    - Mild: intermittent paraesthesia in the distribution of the median nerve
    - Moderate: constant paraesthesia, and reversible numbness or pain of idiopathic nature
    - Severe: constant pain, numbness or sensory loss in the wrist and hand (specifically palm, index, or middle finger, or thumb), or thenar muscle atrophy
- Able to read and write in simplified Chinese (Mainland), understand and complete the questionnaire, and should provide informed consent.

Exclusion criteria

- CaTS secondary to wrist deformity, trauma, mass, pregnancy, hypothyroidism, or inflammatory arthropathy;
- Treatment by NS, US or injection within the past 6 months, or previous carpal tunnel surgery;
- Previous surgery on the affected wrist (or study wrist if bilateral CaTS);
- Unable to tolerate the study interventions;
- Trauma to the affected upper limb requiring operation or immobilization within the past 12 months;

For peer review only

For peer review only - http://bmjopen.bmj.com/site/about/guidelines.xhtml

- Inter-current illness including, poorly controlled diabetes mellitus or thyroid disease, vibration-induced neuropathy, osteoarthritis or inflammatory joint disease, suspected complex neurological and musculoskeletal conditions, et al.;
- Known drugs or alcohol abuse;
- Allergy to any of the splint materials;
- Contraindications to US, including dermatological conditions, abnormal sensation in the affected arm, indwelling electrical pumps/pacemakers, epilepsy, pregnancy or breastfeeding, et al.

Following the medical evaluation, a research assistant will meet with the eligible participants and obtain their written informed consent. Demographic variables such as age, sex, body mass index, affected elbow (whether bilateral), dominant arm, lifestyle (smoking and alcohol use), and medical history of all participants will be collected prior to treatment (baseline). Participants will also be asked relevant questions about symptoms duration and previous treatments (rehabilitation exercises, injections or others). Others like occupation, employment characteristics (full-time or part-time work, manual or non-manual labor), employment status (whether on sickness absence), and professional activity characteristics (repetitive movements for >4 hours/day; wrist flexion and extension for >2 hours/day and use of computer keyboard/ mouse [how many hours/day]) will be also collected.

Randomization and blinding

Participants will be randomized in three intervention groups (either US or NS or US+NS arm) in a ratio of 1:1:1, using a computer-generated randomized sequence with varying unknown block sizes (either 3 or 6) for all study centres, without stratification. A research assistant who are not involved in clinical care and participant evaluations will prepare sequentially numbered, opaque, sealed envelopes based on a

For peer review only - http://bmjopen.bmj.com/site/about/guidelines.xhtml

random list, and ensure that the allocation data will not be accessed or influenced by anyone. When appropriate, the assistant will open envelopes and ensure coordination of therapeutic interventions.

The outcome assessor and statistician will be blinded to group allocation and not involved in treatment procedures.

Intervention

At the beginning, all participants will participate in an about 30-minute group educational presentation by a research assistant on the same day as the baseline assessment. This presentation will cover the pathophysiology, treatment options, posture and activity modification principles of CaTS. The above information will also be provided to participants in the form of education booklets, to encourage them to review at home. Habits changes include limited wrist movement and a reduction in strenuous work activities, and the use of ergonomically friendly work tools helps reduce median nerve pressure.[32,33]

Participants in the [US group] will receive continuous mode US (Shanghai, China) at a frequency of 1 MHz and intensity of 1.0 W/cm^2 for 15 minutes in 5 days per week for 6 weeks to the area over the carpal tunnel.

Participants allocated to the [NS group] will receive a splint to wear at night for 6 weeks. The splint holds the wrist in a neutral position or slightly extended 20° from the neutral position to avoid wrist movement, which can increase pressure on the carpal tunnel.[34] The choose of each splint will be based on the size of individual participant's hands and arms. Participants will be shown how to fit and remove the wrist splint according to a standardized trial protocol. Oral guidance from the clinician on how and when to use splints will encourage and reinforce compliance, which will be also supported by written information, detailed care, and splint fitting

For peer review only - http://bmjopen.bmj.com/site/about/guidelines.xhtml

and use. Participants will be instructed to perform gentle range-of-motion exercises when removing the splint to prevent stiffness.

Participants randomized to the [US+NS group] will receive both US for 6 weeks as in the [US group] and NS for 6 weeks as in the [NS group].

For participants with bilateral CTS, the non-study hand will be treated according to normal clinical protocols in use at the research site.

We discouraged additional treatments to that assigned (that is, not per protocol) during the intervention period, but we allowed the use of simple analgesics as needed. Participants reported all not per protocol treatments, such as drugs, in a diary.

Data management

Data will be collected during the participants' visits to the hospital at baseline, 6 weeks, 3 months, 6 months, and one-year after random assignment (**Table 1**). Reminder emails and phone calls from the research assistants will be programmed to maximize participant compliance in subsequent completion. A registered participant will withdraw from the study if (1) the participant withdraws his/her consent, and (2) exclusion criteria is found after registration. The cause and date of suspension will be recorded. Consent to use data that has been collected before the participant's withdrawal will be included in the consent form.

Primary outcome measure

The primary outcome measure will be the difference in Symptom Severity Scale of the Boston Carpal Tunnel Questionnaire (BCTQ-SSS) at 6-week. The BCTQ is a disease-specific questionnaire referring to a typical 24-hour period in the past two weeks,[35] and has been shown to be highly reproducible, internally consistent, valid and responsive to clinical change in CaTS.[36] It consists of two different subscales: Symptom Severity Scale (11 items, about symptom severity) and Functional Status

For peer review only - http://bmjopen.bmj.com/site/about/guidelines.xhtml

Scale (8 items, about the degrees of difficulty on daily activities), both rated on a five-point scale, with final scores for each subscale result in mean scores between 1 and 5. The overall score is calculated as the mean of all 19 items. Higher scores represent more severe symptoms and functional impairment. We use a validated Chinese version[37] of the BCTQ in this study.

Secondary outcome

Secondary outcome measures will be the differences in Functional Status Scale of the Boston Carpal Tunnel Questionnaire (BCTQ-FSS), numerical rating scale (NRS)[38,39] for hand-wrist symptom intensity, shortened version of the Disabilities of the Arm, Shoulder and Hand (quick-DASH)[40] for upper limb disability, sleep questionnaire for interrupted sleep[41], EuroQol-5D (EQ-5D)[42] for life quality and health status, Hospital Anxiety and Depression Scale (HADS)[43] for anxiety and depression status, Work Limitations Questionnaire (WLQ)-25[44] for functional limitations at work, Global Rating of Change (GROC) for treatment success and recurrence rate, Likert scale for participant's satisfaction, physical function examination as well as various electrophysiology and ultrasound parameters.

■ Hand-wrist symptom intensity (pain severity and paresthesia/dysthesia)

The NRS will be used for symptom intensity evaluation, which is a 0-10 numerical rating scale. 0 point represents "no symptom", and 10 points represents "extremely severe symptom".[38,39] A minimum decrease of 1.3 points or 25% reduction in NRS is considered the minimal clinically important difference.[45,46]

■ Upper limb disability

Elbow function assessment will be conducted using a well-validated Simplified Chinese (Mainland) Version of Quick-DASH,[47] consisting of 11 questions on a 5-point scale similar to the DASH.[40] The overall score and individual modules will be

For peer review only - http://bmjopen.bmj.com/site/about/guidelines.xhtml

calculated out of 100 points, with higher scores indicating worse status. The minimal clinically important difference was reported to be 15.91 points.[48]

■ Interrupted sleep

The sleep questionnaire, which will be used to assess sleep quality, consists of four questions asking participants how many times they have experienced it in the last month.[41] Each question has six response categories and are coded in 0-5 order: not at all, 1-3, 4-7, 8-14, 15-21 and 22-31 days. All questions have equal weights and add up. Higher scores are associated with more disrupted sleep.

■ Life quality and health status

EQ-5D has been widely validated and used to measure generic health-related quality of life (HRQol) due to its simplicity.[42] It consists of a five-dimensional description system (mobility, self-care, usual activities, pain/discomfort and anxiety/depression) and a VAS that scales from 0 to 1, where 1 represents perfect health. All dimensions are divided into three levels (no problem, some problem and extreme problem). We used a validated Chinese version[49,50] of EQ-5D, which has been recommended by Guidelines for Pharmacoeconomic Evaluations 2011 for a measure for HRQol and health utility.[51]

■ Anxiety and depression status

HADS will be used to identify and quantify two of the most common psychological disorders - anxiety and depression.[43] There is evidence of increased anxiety and depression in LET patients.[52] The HADS is a 14-item scale independent of physical symptoms and consists of two 7-item subscales measuring depression and anxiety, respectively. A 4-point scale (0 for absence of symptoms, and 3 for maximum symptomatology) is used. Each subscale has an overall score ranging from 0 to 21, with a higher score indicating a higher degree of impairment. HADS has two

For peer review only - http://bmjopen.bmj.com/site/about/guidelines.xhtml

cut offs for categorization: 0-7, “non-case”; 8-10, “possible or doubtful case”; 11-21, “probable or definite case”.[53]

■ Functional limitations at work

To gather information that is complementary to the pain and disability scales, functional limitations at work will be measured using WLQ-25. It contains 25 items, arranged under four sub-scales, covering four dimensions of job demands, namely time demands, physical demands, mental/interpersonal demands, and output demands.[44] A five-level ordinal response scale ranging from 0 (all of the time) to 4 (none of the time) with an additional sixth option (does not apply to my job) is used. The overall score ranges from 0 to 100, with an increase of 13 points (out of 100) for clinically important differences.[54]

■ Treatment success and recurrence rate

Participants' treatment impressions of changes in their condition (ranging from “completely recovered”, “much improved”, “somewhat improved”, “same”, “worse” to “much worse”) will be recorded on a six-level Likert scale. The success rate will be calculated by dichotomizing the response. Participants who report " completely recovered " or " much improved " in their overall condition since the study beginning will be considered successful, while other responses will be considered failures.[55,56] Recurrence will be defined primarily as when a participant rates a success at 6 weeks and a failure at 3 months, 6 months or one-year on GROC.[55,56]

■ Participants’ satisfaction

Similarly, participants’ level of satisfaction on the evolution of their condition will be determined on a validated 5-point Likert scale ranging from “very satisfied”, “quite satisfied”, “no opinion”, “not very satisfied” to “not at all satisfied”.[57,58]

■ Physical function examination

For peer review only - http://bmjopen.bmj.com/site/about/guidelines.xhtml

The physical examinations will include measurement of 2-point discrimination (performed on the radial and ulnar aspects of each digit), grip strength with a dynamometer (CAMRY, City of Industry, CA, USA), and pinch strength with the pinch gauge (three trials for each hand). The affected side will be measured first and then the unaffected side. The measurement readings will be not revealed to the subjects until the completion of the test. The mean of three consecutive trials, separated by a 20s pause, will be calculated. Results will be presented as a ratio of values of the symptomatic side/ asymptomatic side×100.[59]

The 2-point discrimination test starts at a distance of 4 mm and increases continuously by 2 mm as necessary. Grip strength and 3-point pinching force (3 tests per hand) as measured with baseline dynamometer and pinch gauge (Chattanooga Group, Hixson, Tennessee, USA) will be recorded.

■ Electrophysiological Study

Median nerve distal motor latency (DML), compound muscle action potential (CMAP), sensory nerve conduction velocity (SNCV) and sensory nerve action potential (SNAP) amplitudes will be recorded.[55,56] DML and CMAP will be measured by placing surface electrodes on the abductor pollicis brevis muscle, and stimulation applied 8 cm proximal to the active recording electrode. SNAP and SNCV will be obtained using ring electrodes placed on the proximal and distal interphalangeal joints of the index finger. Sensory conduction will be studied by antidromically stimulation at 14 cm proximal to the active electrode. Motor study will be performed by supramaximal stimulation while the amplitude will be measured an average of 10 times for the sensory study. All measurements will be made 3 times, and the values obtained will be averaged for analysis.

■ US parameters

For peer review only - http://bmjopen.bmj.com/site/about/guidelines.xhtml

The cross-sectional area (CSA) will be measured using an electronic caliper at the scaphoid-pisiform level.[38,39] The measurements will be made 3 times, and the values obtained will be averaged for analysis.

Adverse events

All adverse events, defined as any negative or unwanted reactions to intervention, will be recorded through the symptoms reported by the patients, and observations by a researcher at every visit. US treatment may cause mild local swelling, spot-like bleeding, ecchymosis, enhanced local pain response, and local hyperesthesia or decrease. NS treatment may cause skin allergy, wrist stiffness, et al. The participants will be instructed to do gentle range-of-motion exercises when removing the splint to prevent stiffness and reinforced adherence by verbal instruction.

Sample size calculation

Sample size and power calculation are based on the primary outcome of BCTQ-SSS score at 6-week. All sample size calculations assume two-sided analysis with a power of 90% ($1-\beta=0.90$) at a significant level of $\alpha=0.05$. Based on a published RCT trial ([Lancet. 2018;392:1423-1433], the authors compared corticosteroid injection and NS for CaTS, and participants in the NS group had a standard deviation [SD] of 0.76 points for BCTQ-SSS at their 6-week followup, ["6-week" was the primary endpoint in this study]),[9] a SD of 0.76-point on BCTQ-SSS score will be used. To detect a minimum clinically significant difference of 0.8-point[58] (superiority margin) between US+NS and NS groups (assuming a true difference of 1.19-point[9,60]), a total of 48 participants in each group is required. Allowing for an up to 10% drop out rate, we aim to enroll at least 54 participants in each group to complete the study.

Analysis plan

For peer review only - http://bmjopen.bmj.com/site/about/guidelines.xhtml

Baseline characteristics of the three treatment groups will be summarized using appropriate descriptive statistics. Both the primary and secondary analyses will be blind analyses of treatment assignments and will be performed using the intention-to-treat (ITT)[61] method, with all randomized participants retaining their original assigned group. Multiple imputation by chained equations will be used to address missing data caused by lost access and non-response if these missing data are judged to be random.

The primary comparisons for BCTQ-SSS scores will be made using linear regression. In secondary analyses, repeated measures mixed model[62] will also be used to examine the associations between treatments and repeated outcome measures, with terms of treatment, time, trial center and corresponding baseline values as covariates (age, gender, body mass index, et al.). Bonferroni method will be used to adjust for multiplicity.[63,64] Linear regression will be used for numerical outcomes, and logistic/ordinal regression for any categorical outcomes.

Quality assurance/monitoring/management

In order to standardize the procedures of staff training and learning, such as participants recruitment, outcome measures, data import, security, management and analysis, a Manual of Operations and Procedures and a case report form will be developed as per protocol, which also include the monitoring plans to assure participant protection and data integrity, thus facilitating consistency in protocol implementation and data collection. The investigators, physicians, research assistants, outcome assessors and statisticians are different people and should be trained in good clinical practice. Trained project managers will visit each centre for monitoring to ensure data quality and compliance with the trial protocol.

All data obtained will be stored electronically and strictly in a database with secure and restricted access. Encryption will be used for data transmission, with

For peer review only

For peer review only - http://bmjopen.bmj.com/site/about/guidelines.xhtml

removal for any information that can identify individuals. Data will only be de-identified for analysis at the completion of this study.

Study duration

Recruitment of the trial will begin in the November of 2021 and one-year follow-up for all participants is anticipated to be completed by June 2023. See **Table 1** for time points and recruitment progress.

Ethics and dissemination

This study has been approved by the Ethics Committee of Shanghai Sixth People's Hospital (lead Clinical Center, approval No. 2021-152), Ethics Committee of Shanghai East Hospital (EC.D(BG).016.03.1-2021-095), Ethics Committee of Shanghai Tenth People's Hospital (SHSY-IEC-4.1/21-194/01), and Ethics Committee of Pudong New Area People's Hospital (IRBY2021-006). The potential risks of this clinical trial are considered minimal and are addressed in the protocol and consent form. A written consent (**Supplemental file 1**) will be obtained by clinical practitioners from each participant. The trial was registered on www.chictr.org website (registration number ChiCTR2100050701). Data will be published in peer-reviewed journals and presented at conferences, both nationally and internationally.

For peer review only - http://bmjopen.bmj.com/site/about/guidelines.xhtml

474 **DISCUSSION**

475 CaTS is a highly prevalent compression neuropathy, which results in significant paraesthesia and numbness in the median nerve distribution, especially nocturnal symptoms causing sleep disturbance, causing great socioeconomic burden. Up till now, there is still no consensus on the optimal management, and nonoperative treatment is generally accepted as the first-line intervention for mild and moderate CaTS. Multiple methods have been studied and reviewed in the recent decades, however, the exact efficacy still remains controversial.

482 In an RCT published in BMJ for mild to moderate CaTS, active US treatment (1 MHz, 1.0 W/cm^2) was applied to the area over the carpal tunnel in the experimental group, and indistinguishable sham US treatment was applied in the control group.[23] At 6 months' follow up, satisfactory improvement or complete remission of symptoms was observed in 74% receiving active treatment, which is significantly higher than those receiving sham treatment (20%). As for electroneurography, DML and SNCV improved significantly with active treatment while remained unchanged with sham treatment. Hand grip and finger pinch strength in physical examination also improved significantly with active treatment. All results suggested satisfying effects from US treatment for CaTS.

492 US can also be used as part of a multi-intervention approach. Some studies have compared NS alone to NS combined with US in treatment of CaTS, while the effects were different, and the grades of study design and data were low. Dincer U et al.[26] found that the improvements in the combined group were statistically significantly better (p=0.043) than those in NS alone group in BCTQ-SSS, as well as BCTQ-FSS (p<0.001), and VAS for pain (p<0.001). Similar results were also reported by Baysal

For peer review only - http://bmjopen.bmj.com/site/about/guidelines.xhtml

O et al.[65]; while Jothi KP et al.[27], Sim SE et al.[28] and Armagan O et al.[29] found US therapy may add no benefit to splinting in CaTS.

In this study, to the best of our knowledge, it is the first to compared the efficacy between US [US group] and NS [NS group] in CaTS treatment. What's more, the additional effects of US [US+NS group] in a multi-intervention approach will be compared with NS alone [NS group]. In clinic, US is less invasive, less expensive, safer and more portable than other nonoperative therapy like drug injections for compression neuropathy and, if proved to be effective, could be offered to selected patients as part of non-operative therapy.

There are some on-going clinical trials on CaTS treatment recent years,[66-69] and our prospective randomized study proposes to complement and add to this relevant and much needed scientific effort.

For peer review only - http://bmjopen.bmj.com/site/about/guidelines.xhtml

REFERENCES

1. Seiler JG, 3rd, Daruwalla JH, Payne SH, et al. Normal Palmar Anatomy and Variations That Impact Median Nerve Decompression. J Am Acad Orthop Surg. 2017;25(9):e194-e203.
2. Kleopa KA. In the Clinic. Carpal Tunnel Syndrome. Ann Intern Med. 2015;163-5:Itc1.
3. Feng B, Chen K, Zhu X, et al. Prevalence and risk factors of self-reported wrist and hand symptoms and clinically confirmed carpal tunnel syndrome among office workers in China: a cross-sectional study. BMC Public Health. 2021;21(1):57.
4. Latinovic R, Gulliford MC, Hughes RA. Incidence of common compressive neuropathies in primary care. J Neurol Neurosurg Psychiatry. 2006;77(2):263-5.
5. Jerosch-Herold C, Houghton J, Blake J, et al. Association of psychological distress, quality of life and costs with carpal tunnel syndrome severity: a cross-sectional analysis of the PALMS cohort. BMJ Open. 2017;7(11):e017732.
6. Werner RA, Franzblau A, Gell N. Randomized controlled trial of nocturnal splinting for active workers with symptoms of carpal tunnel syndrome. Arch Phys Med Rehabil. 2005;86(1):1-7.
7. Padua L, Coraci D, Erra C, et al. Carpal tunnel syndrome: clinical features, diagnosis, and management. Lancet Neurol. 2016;15(12):1273-84.
8. Graham B. The value added by electrodiagnostic testing in the diagnosis of carpal tunnel syndrome. J Bone Joint Surg Am. 2008;90(12):2587-93.
9. Chesterton LS, Blagojevic-Bucknall M, Burton C, et al. The clinical and cost-effectiveness of corticosteroid injection versus night splints for carpal tunnel

For peer review only

syndrome (INSTINCTS trial): an open-label, parallel group, randomised controlled trial. Lancet. 2018;392(10156):1423-33.

10. Jiménez Del Barrio S, Estébanez de Miguel E, Bueno Gracia E, et al. Effects of diacutaneous fibrolysis in patients with mild to moderate symptomatic carpal tunnel syndrome: a randomized controlled trial. Clin Rehabil. 2018;32(12):1645-55.

11. Huisstede BM, Fridén J, Coert JH, et al. Carpal tunnel syndrome: hand surgeons, hand therapists, and physical medicine and rehabilitation physicians agree on a multidisciplinary treatment guideline—results from the European HANDGUIDE Study. Arch Phys Med Rehabil. 2014;95(12):2253-63.

12. Carpal Tunnel Syndrome: A Summary of Clinical Practice Guideline Recommendations-Using the Evidence to Guide Physical Therapist Practice. J Orthop Sports Phys Ther. 2019;49(5):359-60.

13. Graham B, Peljovich AE, Afra R, et al. The American Academy of Orthopaedic Surgeons Evidence-Based Clinical Practice Guideline on: Management of Carpal Tunnel Syndrome. J Bone Joint Surg Am. 2016;98(20):1750-4.

14. Choi GH, Wieland LS, Lee H, et al. Acupuncture and related interventions for the treatment of symptoms associated with carpal tunnel syndrome. Cochrane Database Syst Rev. 2018;12(12):Cd011215.

15. Page MJ, O'Connor D, Pitt V, et al. Exercise and mobilisation interventions for carpal tunnel syndrome. Cochrane Database Syst Rev. 2012(6):Cd009899.

16. Franke TP, Koes BW, Geelen SJ, et al. Do Patients With Carpal Tunnel Syndrome Benefit From Low-Level Laser Therapy? A Systematic Review of Randomized Controlled Trials. Arch Phys Med Rehabil. 2018;99(8):1650-9.e15.

For peer review only

For peer review only - http://bmjopen.bmj.com/site/about/guidelines.xhtml

17. Kim JC, Jung SH, Lee SU, et al. Effect of extracorporeal shockwave therapy on carpal tunnel syndrome: A systematic review and meta-analysis of randomized controlled trials. Medicine (Baltimore). 2019;98(33):e16870.

18. Catapano M, Catapano J, Borschel G, et al. Effectiveness of Platelet-Rich Plasma Injections for Nonsurgical Management of Carpal Tunnel Syndrome: A Systematic Review and Meta-analysis of Randomized Controlled Trials. Arch Phys Med Rehabil. 2020;101(5):897-906.

19. O'Brien WD, Jr. Ultrasound-biophysics mechanisms. Prog Biophys Mol Biol. 2007;93-1(3):212-55.

20. Schuhfried O, Vukanovic D, Kollmann C, et al. Effects of Pulsed Ultrasound Therapy on Sensory Nerve Conduction Parameters and the Pain Threshold Perceptions in Humans. Pm r. 2017;9(8):781-6.

21. Ilham SJ, Chen L, Guo T, et al. In vitro single-unit recordings reveal increased peripheral nerve conduction velocity by focused pulsed ultrasound. Biomed Phys Eng Express. 2018;4(4).

22. de Oliveira Perrucini PD, Poli-Frederico RC, de Almeida Pires-Oliveira DA, et al. Anti-Inflammatory and Healing Effects of Pulsed Ultrasound Therapy on Fibroblasts. Am J Phys Med Rehabil 2020;99(1):19-25.

23. Ebenbichler GR, Resch KL, Nicolakis P, et al. Ultrasound treatment for treating the carpal tunnel syndrome: randomised "sham" controlled trial. BMJ. 1998;316(7133):731-5.

24. Page MJ, O'Connor D, Pitt V, et al. Therapeutic ultrasound for carpal tunnel syndrome. Cochrane Database Syst Rev. 2013;2013(3):Cd009601.

25. Huisstede BM, Hoogvliet P, Franke TP, et al. Carpal Tunnel Syndrome: Effectiveness of Physical Therapy and Electrophysical Modalities. An Updated

For peer review only

Systematic Review of Randomized Controlled Trials. Arch Phys Med Rehabil. 2018;99(8):1623-34.e23.

26. Dincer U, Cakar E, Kiralp MZ, et al. The effectiveness of conservative treatments of carpal tunnel syndrome: splinting, ultrasound, and low-level laser therapies. Photomed Laser Surg. 2009;27(1):119-25.

27. Jothi KP, Bland JDP. Ultrasound therapy adds no benefit to splinting in carpal tunnel syndrome. Muscle Nerve. 2019;60(5):538-43.

28. Sim SE, Gunasagaran J, Goh KJ, et al. Short-term clinical outcome of orthosis alone vs combination of orthosis, nerve, and tendon gliding exercises and ultrasound therapy for treatment of carpal tunnel syndrome. J Hand Ther. 2019;32(4):411-6.

29. Armagan O, Bakilan F, Ozgen M, et al. Effects of placebo-controlled continuous and pulsed ultrasound treatments on carpal tunnel syndrome: a randomized trial. Clinics (Sao Paulo). 2014;69(8):524-8.

30. Chan AW, Tetzlaff JM, Gøtzsche PC, et al. SPIRIT 2013 explanation and elaboration: guidance for protocols of clinical trials. BMJ. 2013;346:e7586.

31. Burton C, Chesterton LS, Davenport G. Diagnosing and managing carpal tunnel syndrome in primary care. Br J Gen Pract. 2014;64(622):262-3.

32. O'Connor D, Page MJ, Marshall SC, et al. Ergonomic positioning or equipment for treating carpal tunnel syndrome. Cochrane Database Syst Rev. 2012;1(1):Cd009600.

33. Buchan S, Amirfeyz R. Cochrane corner: ergonomic positioning or equipment for treating carpal tunnel syndrome. J Hand Surg Eur Vol. 2013;38(5):580-1.

For peer review only

For peer review only - http://bmjopen.bmj.com/site/about/guidelines.xhtml

34. Weiss ND, Gordon L, Bloom T, et al. Position of the wrist associated with the lowest carpal-tunnel pressure: implications for splint design. J Bone Joint Surg Am. 1995;77(11):1695-9.

35. Levine DW, Simmons BP, Koris MJ, et al. A self-administered questionnaire for the assessment of severity of symptoms and functional status in carpal tunnel syndrome. J Bone Joint Surg Am. 1993;75(11):1585-92.

36. Leite JC, Jerosch-Herold C, Song F. A systematic review of the psychometric properties of the Boston Carpal Tunnel Questionnaire. BMC Musculoskelet Disord. 2006;7:78.

37. Lue YJ, Lu YM, Lin GT, et al. Validation of the Chinese version of the Boston Carpal Tunnel Questionnaire. J Occup Rehabil. 2014;24(1):139-45.

38. Wu YT, Ke MJ, Ho TY, et al. Randomized double-blinded clinical trial of 5% dextrose versus triamcinolone injection for carpal tunnel syndrome patients. Ann Neurol. 2018;84(4):601-10.

39. Wu YT, Ho TY, Chou YC, et al. Six-month Efficacy of Perineural Dextrose for Carpal Tunnel Syndrome: A Prospective, Randomized, Double-Blind, Controlled Trial. Mayo Clin Proc. 2017;92(8):1179-89.

40. Beaton DE, Wright JG, Katz JN. Development of the QuickDASH: comparison of three item-reduction approaches. J Bone Joint Surg Am. 2005;87(5):1038-46.

41. Jenkins CD, Stanton BA, Niemcryk SJ, et al. A scale for the estimation of sleep problems in clinical research. J Clin Epidemiol. 1988;41(4):313-21.

42. EuroQol--a new facility for the measurement of health-related quality of life. Health Policy. 1990;16(3):199-208.

43. Zigmond AS, Snaith RP. The hospital anxiety and depression scale. Acta Psychiatr Scand. 1983;67(6):361-70.

For peer review only - http://bmjopen.bmj.com/site/about/guidelines.xhtml

632 44. Lerner D, Amick BC 3rd, Rogers WH, et al. The Work Limitations Questionnaire. Med Care. 2001;39(1):72-85.

634 45. Spadoni GF, Stratford PW, Solomon PE, et al. The evaluation of change in pain intensity: a comparison of the P4 and single-item numeric pain rating scales. J Orthop Sports Phys Ther. 2004;34(4):187-93.

637 46. Bijur PE, Chang AK, Esses D, et al. Identifying the minimum clinically significant difference in acute pain in the elderly. Ann Emerg Med. 2010;56(5):517-21.e1.

640 47. Cao S, Zhou R, Zhou H, et al. Reliability and validity of Simplified Chinese version of Quick Disabilities of the Arm, Shoulder, and Hand (QuickDASH) questionnaire: cross-cultural adaptation and validation. Clin Rheumatol. 2019;38(11):3281-7.

644 48. Franchignoni F, Vercelli S, Giordano A, et al. Minimal clinically important difference of the disabilities of the arm, shoulder and hand outcome measure (DASH) and its shortened version (QuickDASH). J Orthop Sports Phys Ther. 2014;44(1):30-9.

648 49. Wu C, Gong Y, Wu J, et al. Chinese Version of the EQ-5D Preference Weights: Applicability in a Chinese General Population. PLoS One. 2016;11(10):e0164334.

650 50. Luo N, Liu G, Li M, et al. Estimating an EQ-5D-5L Value Set for China. Value Health. 2017;20(4):662-9.

652 51. Sun S, Chen J, Johannesson M, et al. Population health status in China: EQ-5D results, by age, sex and socio-economic status, from the National Health Services Survey 2008. Qual Life Res. 2011;20(3):309-20.

655 52. Alizadehkhaiyat O, Fisher AC, Kemp GJ, et al. Pain, functional disability, and psychologic status in tennis elbow. Clin J Pain. 2007;23(6):482-9.

For peer review only - http://bmjopen.bmj.com/site/about/guidelines.xhtml

53. Pallant JF, Bailey CM. Assessment of the structure of the Hospital Anxiety and Depression Scale in musculoskeletal patients. Health Qual Life Outcomes. 2005;3:82.

54. Roy JS, MacDermid JC, Amick BC 3rd, et al. Validity and responsiveness of presenteeism scales in chronic work-related upper-extremity disorders. Phys Ther. 2011;91(2):254-66.

55. Wang JC, Liao KK, Lin KP, et al. Efficacy of Combined Ultrasound-Guided Steroid Injection and Splinting in Patients With Carpal Tunnel Syndrome: A Randomized Controlled Trial. Arch Phys Med Rehabil 2017;98(5):947-56.

56. Hsu PC, Liao KK, Lin KP, et al. Comparison of Corticosteroid Injection Dosages in Mild to Moderate Idiopathic Carpal Tunnel Syndrome: A Randomized Controlled Trial. Arch Phys Med Rehabil. 2020;101(11):1857-64.

57. Hay E, Barlas P, Foster N, et al. Is acupuncture a useful adjunct to physiotherapy for older adults with knee pain?: the "acupuncture, physiotherapy and exercise" (APEX) study [ISRCTN88597683]. BMC Musculoskelet Disord. 2004;5:31.

58. Chesterton LS, Dziedzic KS, van der Windt DA, et al. The clinical and cost effectiveness of steroid injection compared with night splints for carpal tunnel syndrome: the INSTINCTS randomised clinical trial study protocol. BMC Musculoskelet Disord. 2016;17(1):415.

59. Smidt N, van der Windt DA, Assendelft WJ, et al. Interobserver reproducibility of the assessment of severity of complaints, grip strength, and pressure pain threshold in patients with lateral epicondylitis. Arch Phys Med Rehabil. 2002;83(8):1145-50.

For peer review only - http://bmjopen.bmj.com/site/about/guidelines.xhtml

60. Boonhong J, Thienkul W. Effectiveness of Phonophoresis Treatment in Carpal Tunnel Syndrome: A Randomized Double-blind, Controlled Trial. PM R. 2020;12(1):8-15.

61. Sedgwick P. Intention to treat analysis versus per protocol analysis of trial data. BMJ. 2015;350:h681.

62. Detry MA, Ma Y. Analyzing Repeated Measurements Using Mixed Models. JAMA. 2016;315(4):407-8.

63. Thybo KH, Jakobsen JC, Hägi-Pedersen D, et al. PANSAID-PAracetamol and NSAID in combination: detailed statistical analysis plan for a randomised, blinded, parallel, four-group multicentre clinical trial. Trials. 2017;18(1):465.

64. Thybo KH, Hägi-Pedersen D, Dahl JB, et al. Effect of Combination of Paracetamol (Acetaminophen) and Ibuprofen vs Either Alone on Patient-Controlled Morphine Consumption in the First 24 Hours After Total Hip Arthroplasty: The PANSAID Randomized Clinical Trial. JAMA. 2019;321(6):562-571.

65. Baysal O, Altay Z, Ozcan C, et al. Comparison of three conservative treatment protocols in carpal tunnel syndrome. Int J Clin Pract. 2006;60(7):820-8.

66. Atroshi I, Tadjerbashi K, McCabe SJ, et al. Treatment of carpal tunnel syndrome with wrist splinting: study protocol for a randomized placebo-controlled trial. Trials. 2019;20(1):531.

67. Chen CC, Wu YT, Su YC, et al. Efficacy of laser acupuncture for carpal tunnel syndrome: A study protocol for a prospective double-blind randomized controlled trial. Medicine (Baltimore). 2019;98(30):e16516.

For peer review only

703 68. Dimitrova A, Murchison C, Oken B. Local effects of acupuncture on the median and ulnar nerves in patients with carpal tunnel syndrome: a pilot mechanistic study protocol. Trials. 2019;20(1):8.

706 69. Mason W, Ryan D, Khan A, et al. Injection versus Decompression for Carpal Tunnel Syndrome-Pilot trial (INDICATE-P)-protocol for a randomised feasibility study. Pilot Feasibility Stud. 2017;3:20.

709

For peer review only

For peer review only - http://bmjopen.bmj.com/site/about/guidelines.xhtml

Figure Legends

Figure 1 Participant flow chart

For peer review only

For peer review only - http://bmjopen.bmj.com/site/about/guidelines.xhtml

Table 1 Study evaluation procedures and timeline

**Study procedure**	**Medical evaluation**	**Enrolment visit**	**6 weeks**	**3 months**	**6 months**	**One year**
Determine eligibility	√	√				
Obtain signed consent		√				
Obtain medical and demographic data		√				
Give instructions for Pain medication diary		√				
Outcome measures						
Boston Carpal Tunnel Questionnaire		√	√	√	√	√
Numerical rating scale for hand-wrist symptom intensity		√	√	√	√	√
Shortened version of the Disabilities of the Arm, Shoulder and Hand questionnaire		√	√	√	√	√
Interrupted sleep questionnaire		√	√	√	√	√
EuroQol-5D		√	√	√	√	√
Hospital Anxiety and Depression Scale		√	√	√	√	√
Work Limitations Questionnaire-25		√	√	√	√	√
Treatment success rate			√	√	√	√
Treatment recurrence rate				√	√	√
Participants' satisfaction			√	√	√	√

For peer review only

For peer review only - http://bmjopen.bmj.com/site/about/guidelines.xhtml

Physical examination	√	√	√	√	√
Electrophysiological and ultrasound parameters	√	√	√	√	√

For peer review only

For peer review only - http://bmjopen.bmj.com/site/about/guidelines.xhtml

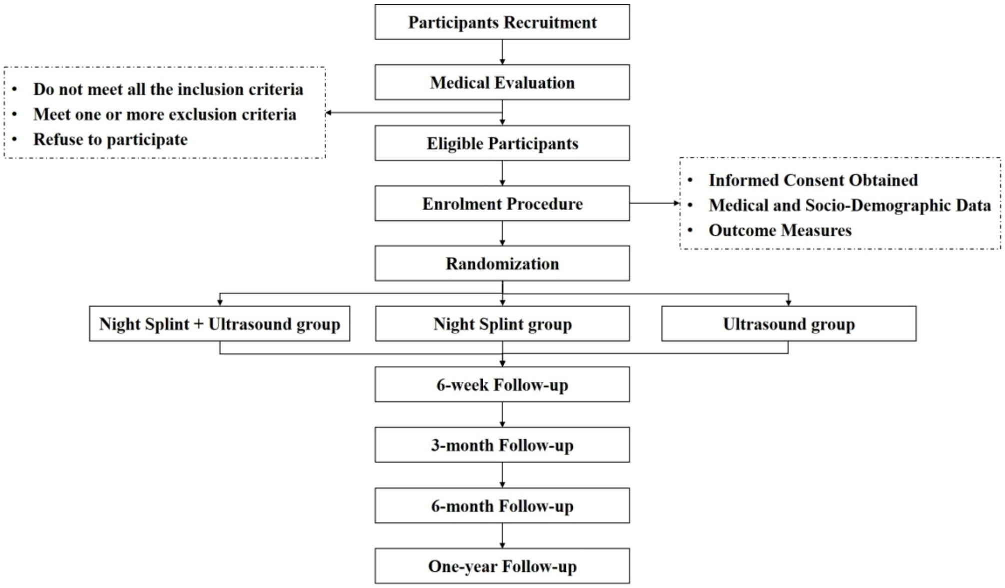

Figure 1 Participant flow chart

365x212mm (150 x 150 DPI)

INFORMED CONSENT FORM

(English Version)

Participant Information Page

**Study Title**	:	Effectiveness of ultrasound therapy for the treatment of carpal tunnel syndrome
**Principal Investigator**	:	Cunyi Fan
**Sponsor**	:	Shanghai Sixth People's Hospital

For peer review only

Dear participant:

You have been diagnosed with lateral elbow tendinopathy, and will be invited to participate in the study named "Effectiveness of ultrasound therapy for the treatment of carpal tunnel syndrome". The study is conducted by the researchers themselves. Please read this informed consent carefully and make the decision whether to participate in this study or not. Participation in this study is entirely your choice. As a participant, you must give your written consent prior to joining the clinical study. When your doctor or researcher discusses informed consent with you, you can ask him or her to explain to you what you don't understand. We encourage you to discuss this thoroughly with your family and friends before making any decision to participate in this study. You have the right to refuse to participate in the study or withdraw from the study at any time without being penalized or losing your rights. If you are participating in another study, please inform your study doctor or investigator. The background, purpose, process and other important information of this study are as follows:

1. BACKGROUND

Carpal tunnel syndrome (CaTS), the most common compression neuropathy, results from median nerve entrapment in the carpal tunnel, accounting for about 90% of all such disorders. The clinically confirmed CaTS prevalence was 9.6% in the general population of China, with a yearly incidence rate of 2.76‰, and women is more susceptible than men. CaTS has significant impact on daily life and ability to work, and causes great burden on social economy, with an annual associated cost estimated at $13 billion. Classically, CaTS causes discomfort, paraesthesia and numbness in the median nerve distribution; and nocturnal symptoms are often clinically significant causing sleep disturbance. Patients can

For peer review only - http://bmjopen.bmj.com/site/about/guidelines.xhtml

be diagnosed by clinical history and physical examination; while electrophysiological methods will be additional for insufficient diagnosis by clinical findings and severe cases that need surgical management.

In general, the severity of CaTS can be classified into mild, moderate and severe. Non-surgical interventions are suggested to be the first choice to treat mild and moderate CaTS. To date, though the treatment method is vast; however, no successful and universally accepted regimen has been established. A consensus of multidisciplinary treatment guideline from the European HANDGUIDE Study suggests that "education" should be included as the first-line management approach, which has the advantages of low cost, high efficacy and non-invasive. In addition, "night splint (NS)" and "corticosteroid injection (CI)" are also recommended in guidelines of American Academy of Orthopaedic Surgeons (AAOS) and American Physical Therapy Association (APTA). One recent RCT published in Lancet compares both two methods, and finds that CI has superior clinical effectiveness at 6 weeks than NS, but no differences at 6 months; while CI may bring adverse events like thinning, lightening or darkening of the skin at the injection site, hot flushes and even more pain. Systematic reviews have also shown that the effects of other conservative treatments like acupuncture, exercise and mobilization interventions, laser, extracorporeal shockwave therapy and platelet-rich plasma injection still remain controversial or provide little to no benefit.

Ultrasound (US) is widely used for imaging purposes and regarded as an adjunct to physiotherapy. In the intensity range of 0.5-2.0 W/cm^2, US may have the potential to induce a variety of biophysical effects in tissues. US experiments on stimulation of nerve conduction and regeneration, and discoveries of its anti-inflammatory effects all support that US may promote recovery of nerve compression. An RCT published in BMJ showed more pronounced subjective symptoms and electroneurographic variables for US treatment than sham control in patients with mild to moderate CaTS. However, to our best of knowledge, no study has compared the efficacy between NS and US in CaTS treatment yet. Additionally, some studies have also reported the efficacy of US to be used as part of a multi-intervention approach, but with low grade of study design and data. Therefore, the role of US in CaTS treatment still needs to be further explored by high-quality study.

2. STUDY PURPOSE

The purpose of the current three-arm, prospective, randomized, multicenter trial is to investigate the effectiveness of US in treatment for CaTS, that is, NS+US (combined) versus NS versus US, on clinical and functional outcomes, including Boston Carpal Tunnel

For peer review only - http://bmjopen.bmj.com/site/about/guidelines.xhtml

Questionnaire (BCTQ).

3. STUDY PROCESS

(1) How many people will participate in the study?

About 162 people will participate in the study at 4 municipal tertiary hospitals: Shanghai Sixth People's Hospital (leader unit), Shanghai East Hospital (participating unit), Shanghai Tenth People’s Hospital (participating unit) and Pudong New Area People′s Hospital of Shanghai (participating unit).

(2) What are the study procedures?

Before you are enrolled in the study, your medical history will be asked, and you will be screened for carpal tunnel syndrome by using criteria developed from a consensus survey by the UK Primary Care Rheumatology Society.

After determining that you are eligible to participate in the study based on inclusion and exclusion criteria, you will be collected and randomly assigned to treatment:

A. Characteristic features collection

You will be asked for your age, sex, body mass index, affected elbow (whether bilateral), dominant arm, lifestyle (smoking and alcohol use), and medical history. As well as relevant questions about symptoms duration and previous treatments (rehabilitation exercises, injections or others). Others like occupation, employment characteristics (full-time or part-time work, manual or non-manual labor), employment status (whether on sickness absence), professional activity characteristics, and sports activities will be also collected.

B. Clinical features collection

You will complete the following questionnaires, including Boston Carpal Tunnel Questionnaire (BCTQ) for wrist function and symptom, numerical rating scale (NRS) for hand-wrist symptom intensity, shortened version of the Disabilities of the Arm, Shoulder and Hand (Quick-DASH) for upper limb disability, EuroQol-5D (EQ-5D) for life quality and health status, Hospital Anxiety and Depression Scale (HADS) for anxiety and depression status, Work Limitations Questionnaire (WLQ)-25 for functional limitations at work, Global Rating of Change (GROC) for treatment success and recurrence rate, Likert scale for participant’s satisfaction, as well as physical function examination and various electrophysiology and ultrasound parameters.

C. Treatment by group

At the beginning, all participants will participate in an about 30-minute group

For peer review only - http://bmjopen.bmj.com/site/about/guidelines.xhtml

educational presentation by a research assistant on the same day as the baseline assessment. This presentation will cover the pathophysiology, treatment options, posture and activity modification principles of CaTS. The above information will also be provided to participants in the form of education booklets, to encourage them to review at home. Habits changes include limited wrist movement and a reduction in strenuous work activities, and the use of ergonomically friendly work tools helps reduce median nerve pressure.

You will be randomly assigned to one of three groups, [US group] vs. [NS group] vs. [US+NS group]:

(a) If you are assigned in the [US group], you will receive continuous mode US (Shanghai, China) at a frequency of 1 MHz and intensity of 1.0 W/cm^2 for 15 minutes in 5 days per week for 6 weeks to the area over the carpal tunnel.

(b) If you are allocated to the [NS group], you will receive a splint to wear at night for 6 weeks. The splint holds the wrist in a neutral position or slightly extended 20° from the neutral position to avoid wrist movement, which can increase pressure on the carpal tunnel. The choose of each splint will be based on the size of each of your hands and arms. You will be shown how to fit and remove the wrist splint according to a standardized trial protocol. Oral guidance from the clinician on how and when to use splints will encourage and reinforce compliance, which will be also supported by written information, detailed care, and splint fitting and use. You will be instructed to perform gentle range-of-motion exercises when removing the splint to prevent stiffness.

(c) If you are randomized to the [US+NS group], you will receive both US for 6 weeks as in the [US group] and NS for 6 weeks as in the [NS group].

We discourage additional treatments to that assigned (that is, not per protocol) during the intervention period, but we allowed the use of simple analgesics as needed. You will report all not per protocol treatments, such as drugs, in a diary.

D. Follow-up features collection

Follow-up data will be collected during your visits to the hospital at 6 weeks, 3 and 6 months, and one year after random assignment.

(3) How long will the study last?

This study will continue for 1 year from the time you receive treatment, and we will collect follow-up information from you at 6 weeks, 3 months, 6 months, and one year at your regular outpatient review.

You may drop out of the study at any time without losing any benefits to which you are entitled. However, if you decide to withdraw during the study, you are encouraged to talk to your doctor first. If you experience a serious adverse event, or if your study doctor

For peer review only - http://bmjopen.bmj.com/site/about/guidelines.xhtml

feels it is not in your best interest to continue in the study, he or she may decide to withdraw you from the study. The sponsor or regulatory agency may also terminate during the study period. However, your withdrawal will not affect your normal medical treatment and rights.

If you withdraw from the study for any reason, you may be asked about your participation in the study. You may also be asked for a medical examination and follow-up questionnaire if your doctor deems it necessary.

(4) Information and biological specimens collected during the study

Biological specimens are not involved in this study, and the information collected is basic characteristics features, preoperative and follow-up clinical features (see the study procedures for details).

All data obtained will be kept strict and stored electronically on a database with secured and restricted access. An encryption will be used for data transfer, with removal for any information able to identify individuals. Data will be only deidentified for analysis at the completion of this study.

4. RISKS AND BENEFITS

(1) What are the risks of participating in this study?

The risks you may incur by participating in this study are as follows. You should discuss these risks with your study doctor or, if you prefer, with your regular care provider.

US treatment may cause mild local swelling, spot-like bleeding, ecchymosis, enhanced local pain response, and local hyperesthesia or decrease. The occurrence of these reactions depends on the dose of treatment, the extent of the lesion, and the individual patient, and usually does not require special treatment. Severe adverse reactions can be treated locally, or prolong the interval of treatment, reduce the intensity of treatment. If the treatment does not improve or abnormal conditions occur, the treatment should be stopped and immediately go to the hospital.

NS treatment may cause skin allergy, wrist stiffness, et al. You will be instructed to do gentle range-of-motion exercises when removing the splint to prevent stiffness and reinforced adherence by verbal instruction.

If you experience any discomfort, new changes, or any unexpected conditions during the study period, whether or not related to the study, you should inform your doctor in a timely manner, and he/she will judge and administer appropriate medical treatment.

During the study period, you need to visit the hospital on time and do some examinations, which will take up some of your time and may cause trouble or

For peer review only - http://bmjopen.bmj.com/site/about/guidelines.xhtml

inconvenience to you.

(2) What are the benefits of participating in the study?

If you agree to participate in this study, you may receive direct medical benefits, such as accelerated relief of symptoms of CaTS. You can also have a deeper understanding of diseases and so on. In addition, we hope that the information gained from your participation in this study will benefit you or other patients with similar conditions in the future.

5. ALTERNATIVE TREATMENT OPTIONS

In addition to participating in this study, you may receive the other treatments provided by your doctor: corticosteroid injection, acupuncture, exercise and mobilization interventions, laser, extracorporeal shockwave therapy and platelet-rich plasma injection, and surgery, etc.

Please discuss these and other possible options with your doctor.

In general, the severity of CaTS can be classified into mild, moderate and severe. Non-surgical interventions are suggested to be the first choice to treat mild and moderate CaTS. To date, though the treatment method is vast; however, no successful and universally accepted regimen has been established. A consensus of multidisciplinary treatment guideline from the European HANDGUIDE Study suggests that "education" should be included as the first-line management approach, which has the advantages of low cost, high efficacy and non-invasive. In addition, "night splint (NS)" and "corticosteroid injection (CI)" are also recommended in guidelines of American Academy of Orthopaedic Surgeons (AAOS) and American Physical Therapy Association (APTA). One recent RCT published in Lancet compares both two methods, and finds that CI has superior clinical effectiveness at 6 weeks than NS, but no differences at 6 months; while CI may bring adverse events like thinning, lightening or darkening of the skin at the injection site, hot flushes and even more pain. Systematic reviews have also shown that the effects of other conservative treatments like acupuncture, exercise and mobilization interventions, laser, extracorporeal shockwave therapy and platelet-rich plasma injection still remain controversial or provide little to no benefit.

6. USE OF RESEACH RESULTS AND CONFIDENTIALITY OF PERSONAL INFORMATION

Results conducted through this program may be published in medical journals with

For peer review only - http://bmjopen.bmj.com/site/about/guidelines.xhtml

the understanding and assistance of you and other participants, but we will keep your study records confidential as required by law.

The personal information of study participants will be kept strictly confidential, and your personal information will not be disclosed unless required by relevant laws.

If necessary, government administrative departments, hospital ethics committees and other relevant researchers can access your data according to regulations.

7. RESEARCH EXPENSES AND RELATED COPENSATION

(1) Cost of drugs/instruments used in the study and related examinations

There are no potential additional costs for this study. Routine outpatient fees include registration, examination for CaTS, oral non-steroidal anti-inflammatory drugs, etc. The expenses related to US and NS will be borne by our research group and funding. In addition, you will be solely responsible for the expenses incurred by you for any treatment other than this study, as well as for the routine treatment and examination required for any concurrent disease.

(2) Compensation for participation in the study

There are no additional compensation costs for this study.

(3) Compensation/compensation after damage

For participants who suffer damage related to this study, the sponsor Shanghai Sixth People's Hospital will bear the treatment cost and corresponding economic compensation in accordance with Chinese laws and regulations.

8. RIGHTS OF PARTICIPANTS AND RELEVANT MATTERS NEEDING ATTENTION

(1) Your rights

Your participation in the study is voluntary throughout the entire process.

If you decide not to participate in this study, it will not affect other treatments you should receive.

If you decide to participate, you will be asked to sign this written informed consent. You have the right to withdraw from the trial at any stage without discrimination or unfair treatment, and your medical treatment and rights will not be affected.

(2) Matters needing attention

For peer review only - http://bmjopen.bmj.com/site/about/guidelines.xhtml

As a subject, you are required to provide true information about your medical history and current medical condition;

Inform the study doctor of any discomfort observed during the study;

Do not take any restricted drugs, food, etc. as advised by your doctor;

Tell the study doctor if you have recently participated in or are currently participating in other studies.

During the intervention, we discouraged additional therapy (i.e., not according to the grouping protocol), but we permitted the use of analgesics when needed (only acetaminophen and NSAIDs).

For medications taken, the name, dose, frequency and duration will be recorded at all follow-up visits.

9. RELEVANT CONTACT INFORMATION

If there is any significant new information during the study that may affect your willingness to continue to participate, your doctor will inform you promptly. If you are interested in your own study data, or you would like to know the findings after this study, you may ask any questions about this study at any time and receive answers accordingly, Please contact doctor Shuai Chen at ***********.

For peer review only

For peer review only - http://bmjopen.bmj.com/site/about/guidelines.xhtml

Participant Signature Page

Informed Consent Statement:

I have been informed of the purpose, background, process, risks and benefits of this study. I have plenty of time and opportunity to ask questions, and I am satisfied with the answers.

I am also told who to contact when I have questions, want to report difficulties, concerns, suggestions for research, or want further information, or to help with research.

I have read this informed consent and agree to participate in this study.

I understand that I may choose not to participate in the study or withdraw from the study at any time during the study without any reason.

I already know that if I get worse, or if I have a serious adverse event, or if my study doctor decides it's not in my best interest to continue, he or she will decide to withdraw me from the study. The funder or regulatory agency may terminate during the study without my consent. If this happens, the doctor will inform me and the study doctor will discuss other options with me.

I will be provided with a copy of the informed consent which contains my signature and that of the investigator.

Participant Signature: ________________
Date: ________________
(NOTE: If participant has no capacity/limited capacity, legal representative signature and date will be required)

Legal Representative's Signature: ________________
Date: ________________

Investigator Signature: ________________
Date: ________________

For peer review only - http://bmjopen.bmj.com/site/about/guidelines.xhtml

Reporting checklist for protocol of a clinical trial.

Based on the SPIRIT guidelines.

Instructions to authors

Complete this checklist by entering the page numbers from your manuscript where readers will find each of the items listed below.

Your article may not currently address all the items on the checklist. Please modify your text to include the missing information. If you are certain that an item does not apply, please write "n/a" and provide a short explanation.

Upload your completed checklist as an extra file when you submit to a journal.

In your methods section, say that you used the SPIRITreporting guidelines, and cite them as:

Chan A-W, Tetzlaff JM, Gøtzsche PC, Altman DG, Mann H, Berlin J, Dickersin K, Hróbjartsson A, Schulz KF, Parulekar WR, Krleža-Jerić K, Laupacis A, Moher D. SPIRIT 2013 Explanation and Elaboration: Guidance for protocols of clinical trials. BMJ. 2013;346:e7586

		Reporting Item	Page Number
**Administrative information**			
Title	#1	Descriptive title identifying the study design, population, interventions, and, if applicable, trial acronym	1
Trial registration	#2a	Trial identifier and registry name. If not yet registered,	4/6

For peer review only

		name of intended registry	
Trial registration: data set	#2b	All items from the World Health Organization Trial Registration Data Set	4/6
Protocol version	#3	Date and version identifier	5
Funding	#4	Sources and types of financial, material, and other support	3
Roles and responsibilities: contributorship	#5a	Names, affiliations, and roles of protocol contributors	2
Roles and responsibilities: sponsor contact information	#5b	Name and contact information for the trial sponsor	2
Roles and responsibilities: sponsor and funder	#5c	Role of study sponsor and funders, if any, in study design; collection, management, analysis, and interpretation of data; writing of the report; and the decision to submit the report for publication, including whether they will have ultimate authority over any of these activities	2
Roles and responsibilities: committees	#5d	Composition, roles, and responsibilities of the coordinating centre, steering committee, endpoint adjudication committee, data management team, and other individuals or groups overseeing the trial, if applicable (see Item 21a for data monitoring committee)	2

Introduction

For peer review only

Background and rationale	#6a	Description of research question and justification for undertaking the trial, including summary of relevant studies (published and unpublished) examining benefits and harms for each intervention	8-9
Background and rationale: choice of comparators	#6b	Explanation for choice of comparators	8-9
Objectives	#7	Specific objectives or hypotheses	10
Trial design	#8	Description of trial design including type of trial (eg, parallel group, crossover, factorial, single group), allocation ratio, and framework (eg, superiority, equivalence, non-inferiority, exploratory)	10
**Methods: Participants, interventions, and outcomes**			
Study setting	#9	Description of study settings (eg, community clinic, academic hospital) and list of countries where data will be collected. Reference to where list of study sites can be obtained	10
Eligibility criteria	#10	Inclusion and exclusion criteria for participants. If applicable, eligibility criteria for study centres and individuals who will perform the interventions (eg,	11-12

For peer review only

		surgeons, psychotherapists)	
Interventions: description	#11a	Interventions for each group with sufficient detail to allow replication, including how and when they will be administered	13-14
Interventions: modifications	#11b	Criteria for discontinuing or modifying allocated interventions for a given trial participant (eg, drug dose change in response to harms, participant request, or improving / worsening disease)	13-14
Interventions: adherance	#11c	Strategies to improve adherence to intervention protocols, and any procedures for monitoring adherence (eg, drug tablet return; laboratory tests)	13-14
Interventions: concomitant care	#11d	Relevant concomitant care and interventions that are permitted or prohibited during the trial	13-14
Outcomes	#12	Primary, secondary, and other outcomes, including the specific measurement variable (eg, systolic blood pressure), analysis metric (eg, change from baseline, final value, time to event), method of aggregation (eg, median, proportion), and time point for each outcome. Explanation of the clinical relevance of chosen efficacy and harm outcomes is strongly recommended	14-19
Participant timeline	#13	Time schedule of enrolment, interventions (including any run-ins and washouts), assessments, and visits for participants. A schematic diagram is highly recommended (see Figure)	20-21

For peer review only

Sample size	#14	Estimated number of participants needed to achieve study objectives and how it was determined, including clinical and statistical assumptions supporting any sample size calculations	19
Recruitment	#15	Strategies for achieving adequate participant enrolment to reach target sample size	10-11
**Methods: Assignment of interventions (for controlled trials)**			
Allocation: sequence generation	#16a	Method of generating the allocation sequence (eg, computer-generated random numbers), and list of any factors for stratification. To reduce predictability of a random sequence, details of any planned restriction (eg, blocking) should be provided in a separate document that is unavailable to those who enrol participants or assign interventions	12-13
Allocation concealment mechanism	#16b	Mechanism of implementing the allocation sequence (eg, central telephone; sequentially numbered, opaque, sealed envelopes), describing any steps to conceal the sequence until interventions are assigned	12-13
Allocation: implementation	#16c	Who will generate the allocation sequence, who will enrol participants, and who will assign participants to interventions	12-13

For peer review only

Blinding (masking)	#17a	Who will be blinded after assignment to interventions (eg, trial participants, care providers, outcome assessors, data analysts), and how	12-13
Blinding (masking): emergency unblinding	#17b	If blinded, circumstances under which unblinding is permissible, and procedure for revealing a participant's allocated intervention during the trial	12-13
**Methods: Data collection, management, and analysis**			
Data collection plan	#18a	Plans for assessment and collection of outcome, baseline, and other trial data, including any related processes to promote data quality (eg, duplicate measurements, training of assessors) and a description of study instruments (eg, questionnaires, laboratory tests) along with their reliability and validity, if known. Reference to where data collection forms can be found, if not in the protocol	14, 19-21
Data collection plan: retention	#18b	Plans to promote participant retention and complete follow-up, including list of any outcome data to be collected for participants who discontinue or deviate from intervention protocols	14, 19-21
Data management	#19	Plans for data entry, coding, security, and storage, including any related processes to promote data quality (eg, double data entry; range checks for data values).	14, 19-21

For peer review only

		Reference to where details of data management procedures can be found, if not in the protocol	
Statistics: outcomes	#20a	Statistical methods for analysing primary and secondary outcomes. Reference to where other details of the statistical analysis plan can be found, if not in the protocol	19-20
Statistics: additional analyses	#20b	Methods for any additional analyses (eg, subgroup and adjusted analyses)	19-20
Statistics: analysis population and missing data	#20c	Definition of analysis population relating to protocol non-adherence (eg, as randomised analysis), and any statistical methods to handle missing data (eg, multiple imputation)	19-20
**Methods: Monitoring**			
Data monitoring: formal committee	#21a	Composition of data monitoring committee (DMC); summary of its role and reporting structure; statement of whether it is independent from the sponsor and competing interests; and reference to where further details about its charter can be found, if not in the protocol. Alternatively, an explanation of why a DMC is not needed	14, 19-21
Data monitoring: interim analysis	#21b	Description of any interim analyses and stopping guidelines, including who will have access to these interim results and make the final decision to terminate the trial	14, 19-21
Harms	#22	Plans for collecting, assessing, reporting, and managing solicited and spontaneously reported adverse events and other unintended effects of trial interventions or trial	19

For peer review only

		conduct	
Auditing	#23	Frequency and procedures for auditing trial conduct, if any, and whether the process will be independent from investigators and the sponsor	19
**Ethics and dissemination**			
Research ethics approval	#24	Plans for seeking research ethics committee / institutional review board (REC / IRB) approval	21
Protocol amendments	#25	Plans for communicating important protocol modifications (eg, changes to eligibility criteria, outcomes, analyses) to relevant parties (eg, investigators, REC / IRBs, trial participants, trial registries, journals, regulators)	21
Consent or assent	#26a	Who will obtain informed consent or assent from potential trial participants or authorised surrogates, and how (see Item 32)	21
Consent or assent: ancillary studies	#26b	Additional consent provisions for collection and use of participant data and biological specimens in ancillary studies, if applicable	21
Confidentiality	#27	How personal information about potential and enrolled participants will be collected, shared, and maintained in order to protect confidentiality before, during, and after the trial	21
Declaration of	#28	Financial and other competing interests for principal	21

For peer review only

interests		investigators for the overall trial and each study site	
Data access	#29	Statement of who will have access to the final trial dataset, and disclosure of contractual agreements that limit such access for investigators	14, 19-21
Ancillary and post trial care	#30	Provisions, if any, for ancillary and post-trial care, and for compensation to those who suffer harm from trial participation	19-21
Dissemination policy: trial results	#31a	Plans for investigators and sponsor to communicate trial results to participants, healthcare professionals, the public, and other relevant groups (eg, via publication, reporting in results databases, or other data sharing arrangements), including any publication restrictions	19-21
Dissemination policy: authorship	#31b	Authorship eligibility guidelines and any intended use of professional writers	19-21
Dissemination policy: reproducible research	#31c	Plans, if any, for granting public access to the full protocol, participant-level dataset, and statistical code	19-21
**Appendices**			
Informed consent materials	#32	Model consent form and other related documentation given to participants and authorised surrogates	21
Biological specimens	#33	Plans for collection, laboratory evaluation, and storage of biological specimens for genetic or molecular analysis in the current trial and for future use in ancillary studies, if applicable	/

For peer review only

None The SPIRIT Explanation and Elaboration paper is distributed under the terms of the Creative Commons Attribution License CC-BY-NC. This checklist can be completed online using https://www.goodreports.org/, a tool made by the EQUATOR Network in collaboration with Penelope.ai

For peer review only

Effectiveness of therapeutic ultrasound for the treatment of carpal tunnel syndrome (the USTINCTS trial): study protocol for a three-arm, prospective, multicenter, randomised controlled trial

Journal:	*BMJ Open*
Manuscript ID	bmjopen-2021-057541.R2
Article Type:	Protocol
Date Submitted by the Author:	15-Mar-2022
Complete List of Authors:	Chen, Shuai; Shanghai Jiao Tong University Affiliated Sixth People's Hospital, Department of Orthopedics; Shanghai Engineering Research Center for Orthopaedic Material Innovation and Tissue Regeneration,<br>Qian, Yun; Shanghai Jiao Tong University Affiliated Sixth People's Hospital, Department of Orthopedics; Shanghai Engineering Research Center for Orthopaedic Material Innovation and Tissue Regeneration<br>Sun, Ziyang; Shanghai Jiao Tong University Affiliated Sixth People's Hospital, Department of Orthopedics; Shanghai Engineering Research Center for Orthopaedic Material Innovation and Tissue Regeneration<br>Liu, Weixuan; Shanghai Jiao Tong University Affiliated Sixth People's Hospital, Department of Orthopedics; Shanghai Engineering Research Center for Orthopaedic Material Innovation and Tissue Regeneration<br>Sun, Guixin; Shanghai East Hospital, Tongji University School of Medicine, Department of Orthopaedics<br>Liu, Junjian; Shanghai Tenth People's Hospital,<br>Wang, Jian; Pudong New Area People's Hospital, Department of Orthopedics<br>Wang, Wei; Shanghai Jiao Tong University Affiliated Sixth People's Hospital, Department of Orthopedics; Shanghai Engineering Research Center for Orthopaedic Material Innovation and Tissue Regeneration<br>Zheng, Yuanyi; Shanghai Jiao Tong University Affiliated Sixth People's Hospital, Department of Ultrasound in Medicine<br>Fan, Cunyi; Shanghai Jiao Tong University Affiliated Sixth People's Hospital, Department of Orthopedics; Shanghai Engineering Research Center for Orthopaedic Material Innovation and Tissue Regeneration
**Primary Subject Heading**:	Sports and exercise medicine
Secondary Subject Heading:	Rehabilitation medicine
Keywords:	REHABILITATION MEDICINE, SPORTS MEDICINE, Hand & wrist < ORTHOPAEDIC & TRAUMA SURGERY, Orthopaedic sports trauma < ORTHOPAEDIC & TRAUMA SURGERY

SCHOLARONE™
Manuscripts

TITLE PAGE

Title

Effectiveness of therapeutic ultrasound for the treatment of carpal tunnel syndrome (the USTINCTS trial): study protocol for a three-arm, prospective, multicenter, randomised controlled trial

Running Title

study protocol of USTINCTS trial for carpal tunnel syndrome

Keywords

Carpal tunnel syndrome, randomised controlled trial, ultrasound therapy, night splint, Boston Carpal Tunnel Questionnaire

Word count

3958 words

Authors and information

Shuai Chen, MD [a, b, 1], Yun Qian, MD [a, b, 1], Ziyang Sun, MD [a, b, 1], Weixuan Liu, MD [a, b], Guixin Sun, MD, PhD [c], JunJian Liu, MD, PhD [d], Jian Wang, MD, PhD [e], Wei Wang, MD [a, b], Yuanyi Zheng, MD, PhD [f, *], Cunyi Fan, MD, PhD [a, b, *]

a Department of Orthopedics, Shanghai Jiao Tong University Affiliated Sixth People's Hospital, Shanghai, 200233, P. R. China

b Shanghai Engineering Research Center for Orthopaedic Material Innovation and Tissue Regeneration, Shanghai, 201306, P. R. China

c Department of Orthopaedics, Shanghai East Hospital, Tongji University School

For peer review only - http://bmjopen.bmj.com/site/about/guidelines.xhtml

of Medicine, Shanghai, 200120, P. R. China

d Department of Orthopedics, Shanghai Tenth People's Hospital, School of Medicine, Tongji University, Shanghai, 200092, P. R. China

e Department of Orthopaedics, Pudong New Area People's Hospital, Shanghai, 201299, P. R. China

f Department of Ultrasound in Medicine, Shanghai Jiao Tong University Affiliated Sixth People’s Hospital, Shanghai, 200233, P. R. China

1 CS, QY and SZY contribute equally to this work as co-first authors.

* C. Fan is corresponding author and Y. Zheng is co-corresponding author of this article.

Corresponding author (Cunyi Fan): ORCID: 0000-0002-7854-5233; E-mail: cyfan@sjtu.edu.cn; Affiliation: Department of Orthopedics, Shanghai Jiao Tong University Affiliated Sixth People’s Hospital, Shanghai, 200233, P. R. China; Address: No. 600 Yishan Road, Shanghai, P. R. China

Co-corresponding author (Yuanyi Zheng): E-mail: zhengyuanyi@163.com; Affiliation: Department of Ultrasound in Medicine, Shanghai Jiao Tong University Affiliated Sixth People’s Hospital, Shanghai, 200233, P. R. China; Address: No. 600 Yishan Road, Shanghai, P. R. China

Author Contributions

CS, QY and SZY are the primary investigators.

CS, QY, SZY, LWX, ZYY, FCY participated in the development of the study design.

CS, QY, SZY, LWX, SGX, LJJ, WJ, WW, ZYY, and FCY participated in the study conduct.

For peer review only

For peer review only - http://bmjopen.bmj.com/site/about/guidelines.xhtml

CS, QY, SZY and LWX drafted the manuscript under FCY's supervision.

FCY and QY contributed to applying for and gaining funding.

All authors contributed to the content and critical revision and approved the final draft of the manuscript.

Conflict of interests

The authors, their immediate families, and any research foundation with which they are affiliated have not received any financial payments or other benefits from any commercial entity related to the subject of this article.

The authors declare no competing financial interests.

Funding

This study will be supported by National Natural Science Foundation of China (8217090787, 82002290); Shanghai Engineering Technology Research Center and Professional Technology Service Platform project of 2020 "Science and Technology Innovation Action Plan" of Shanghai (20DZ2254100); Municipal Hospital Clinical Skills and Innovation Capacity of Three-year Action Plan Program of Shanghai Shenkang Hospital Development Center (SHDC2020CR2039B, SHDC2020CR6019-002); Biomedical Technology Support Special Project of Shanghai "Science and Technology Innovation Action Plan" (20S31900300, 21S31902300); Shanghai Sailing Program (No. 20YF1436000); Young Elite Scientist Sponsorship Program by Cast (No. YESS20200153); Clinical Research Center (CRC) of Shanghai University of Medicine and Health Sciences (20MC2020001).

Acknowledgments

For peer review only

For peer review only - http://bmjopen.bmj.com/site/about/guidelines.xhtml

We will appreciate the support from Base for Interdisciplinary Innovative Talent Training, Shanghai Jiao Tong University and Youth Science and Technology Innovation Studio of Shanghai Jiao Tong University School of Medicine.

ETHICS

The study has been approved by all 4 Medical Ethics Committees, those are, Ethics Committee of Shanghai Sixth People's Hospital (the leading clinical center, approval No. 2021-152), Ethics Committee of Shanghai East Hospital (EC.D(BG).016.03.1-2021-095), Ethics Committee of Shanghai Tenth People's Hospital (SHSY-IEC-4.1/21-194/01), and Ethics Committee of Pudong New Area People's Hospital (IRBY2021-006). The research registry number is ChiCTR2100050701 at http://www.chictr.org.cn. Data will be analyzed anonymously; all patients will approve the results of this study by written consent. The written consent approval will be documented in the patients' files. All clinical investigations will be conducted in accordance with the guidelines of the Declaration of Helsinki.

For peer review only

For peer review only - http://bmjopen.bmj.com/site/about/guidelines.xhtml

ABSTRACT

Introduction

There has no consensus on optimal management of carpal tunnel syndrome (CTS), the most common compression neuropathy. Conservative therapy is generally accepted as first-line intervention. Therapeutic ultrasound has been widely reported to be treatment beneficial in nerve regeneration and conduction, and further accelerate compression recovery. The purpose of this study is to investigate the effectiveness of therapeutic ultrasound for CTS treatment.

Methods and analysis

This study protocol entails a three-arm, prospective, multicenter, randomised controlled trial. 162 eligible adult participants diagnosed with mild to moderate CTS by using criteria developed from a consensus survey by the UK Primary Care Rheumatology Society will be assigned to either (1) therapeutic ultrasound, (2) night splint or (3) therapeutic ultrasound + night splint (combined) group. Primary outcome will be difference in Symptom Severity Scale of Boston Carpal Tunnel Questionnaire (BCTQ-SSS) at 6-week between night splint and therapeutic ultrasound + night splint groups. Secondary outcomes include Functional Status Scale of BCTQ, sleep questionnaire for interrupted sleep, EuroQol-5D for general health, Hospital Anxiety and Depression Scale for mental status, Work Limitations Questionnaire-25 for functional limitations at work, Global Rating of Change for treatment success and recurrence rate, physical examination, electrophysiological and ultrasound parameters. Intention-to-treat analyses will be used.

Ethics and dissemination

Ethics Committees of all clinical centers have approved this study. The leading center is Shanghai Sixth People's Hospital, whose approval number is 2021-152. New

For peer review only - http://bmjopen.bmj.com/site/about/guidelines.xhtml

versions with appropriate amendments will be submitted to the committee for further approval. Final results will be published in peer-reviewed journals and presented at local, national and international conferences.

Trial registration number

ChiCTR2100050701.

For peer review only

For peer review only - http://bmjopen.bmj.com/site/about/guidelines.xhtml

123 STRENGTHS AND LIMITATIONS OF THIS STUDY

124 - Therapeutic ultrasound as independent or adjunct therapy in treating carpal tunnel
125 syndrome (CTS).

126 - The first randomised controlled trial (RCT) to compared the efficacy between
127 therapeutic ultrasound and night splint in CTS treatment.

128 - Multicenter RCT with blinded outcome assessor and statistician.

129 - Use of several patient-reported outcome measures as well as objective
130 parameters.

131 - Participants and treating surgeons not blinded.

132

For peer review only

For peer review only - http://bmjopen.bmj.com/site/about/guidelines.xhtml

INTRODUCTION

Carpal tunnel syndrome (CTS), the most common compression neuropathy, results from median nerve entrapment in the carpal tunnel, accounting for about 90% of all such disorders.[1,2] The clinically confirmed CTS prevalence was 9.6% in the general population of China,[3] with a yearly incidence rate of 2.76‰, and women is more susceptible than men.[2,4] CTS has significant impact on daily life and ability to work,[5] and causes great burden on social economy, with an annual associated costs estimated at $13 billion.[6] Classically, CTS causes discomfort, paraesthesia and numbness in the median nerve distribution; and nocturnal symptoms are often clinically significant causing sleep disturbance.[7] Patients can be diagnosed by clinical history and physical examination; while electrophysiological methods will be additional for insufficient diagnosis by clinical findings and severe cases that need surgical management.[8]

In general, the severity of CTS can be classified into mild, moderate and severe.[9] Non-surgical interventions are suggested to be the first choice to treat mild and moderate CTS.[10] To date, though the treatment method is vast; however, no successful and universally accepted regimen has been established. A consensus of multidisciplinary treatment guideline from the European HANDGUIDE Study suggests that “education” should be included as the first-line management approach, which has the advantages of low cost, high efficacy and non-invasiveness.[7,11] In addition, “night splint” and “corticosteroid injection” are also recommended in guidelines of American Academy of Orthopaedic Surgeons (AAOS) and American Physical Therapy Association (APTA).[12,13] One recent RCT published in Lancet compares both two methods, and finds that corticosteroid injection has superior clinical effectiveness at 6 weeks than night splint, but no differences at 6 months;

For peer review only - http://bmjopen.bmj.com/site/about/guidelines.xhtml

while corticosteroid injection may bring adverse events like thinning, lightening or darkening of the skin at the injection site, hot flushes and even more pain.[9] Systematic reviews have also shown that the effects of other conservative treatments like acupuncture,[14] exercise and mobilization interventions,[15] laser,[16] extracorporeal shockwave therapy[17] and platelet-rich plasma injection[18] still remain controversial or provide little to no benefit.

Therapeutic ultrasound is widely used for imaging purposes and regarded as an adjunct to physiotherapy. In the intensity range of 0.5-2.0 W/cm^2, therapeutic ultrasound may have the potential to induce a variety of biophysical effects in tissues.[19] Therapeutic ultrasound experiments on stimulation of nerve conduction and regeneration,[20,21] and discoveries of its anti-inflammatory effects[22] all support that therapeutic ultrasound may promote recovery of nerve compression. An RCT published in BMJ showed more pronounced subjective symptoms and electroneurographic variables for therapeutic ultrasound than sham control in patients with mild to moderate CTS.[23] However, to our best of knowledge, no study has compared the efficacy between night splint and therapeutic ultrasound in CTS treatment yet. Additionally, some studies have also reported the efficacy of US to be used as part of a multi-intervention approach, but with low grade of study design and data[24-29]. Therefore, the role of therapeutic ultrasound in CTS treatment still needs to be further explored by high-quality study.

Therefore, the purpose of the current three-arm, prospective, randomized, multicenter trial is to examine the effectiveness of therapeutic ultrasound in treatment for CTS, that is, night splint + therapeutic ultrasound (combined) versus night splint versus therapeutic ultrasound, on clinical and functional outcomes, including Boston Carpal Tunnel Questionnaire (BCTQ) in patients diagnosed with CTS.

For peer review only - http://bmjopen.bmj.com/site/about/guidelines.xhtml

METHODS

Study design

This study is a three-arm, prospective, multicenter, randomized controlled trial that will recruit participants from 4 municipal tertiary hospitals with a diagnosis of mild to moderate CTS. The multi-centres are Shanghai Sixth People's Hospital, Shanghai Tenth People's Hospital, Shanghai East Hospital, and Pudong New Area People′s Hospital of Shanghai, respectively. This manuscript is written according to the SPIRIT guidelines.[30]

Participant and public involvement

There was no participant involvement in this study. Participants were not invited to make comments or suggestions on the protocol, were not consulted about the selection of patient-relevant evaluation outcomes, or were invited to participate in writing or editing the manuscript to improve readability or accuracy. The final findings will be disseminated to the public through mass media. Our published papers and conference presentations also acknowledge all participants as a whole.

Participant recruitment

Figure 1 shows the participant flow chart throughout the study. Participants will be recruited over a 5-month period from outpatient clinics of 4 principals in each sub-centre. In addition, recruitment can also be through other doctors and healthcare professionals. Interested participants can contact the research assistant, who will provide more information about the study purpose and protocol, and conduct an initial eligibility screening by phone.

Medical evaluation and enrolment procedure

Eligible participants will be invited to participate in a physical examination to confirm CTS diagnosis and assess eligibility to participate in the program.

For peer review only - http://bmjopen.bmj.com/site/about/guidelines.xhtml

Inclusion criteria

- Age ≥18 years old;
- Clinical diagnosis on the basis of clinical symptoms, history and physical examination, using criteria developed from a consensus survey by the UK Primary Care Rheumatology Society;[31]
- Mild to moderate CTS,[9] with symptoms longer than 6 weeks duration; participants with bilateral CTS will be designated their study hand based on the most severe symptoms;
  - Mild: intermittent paraesthesia in the distribution of the median nerve
  - Moderate: constant paraesthesia, and reversible numbness or pain of idiopathic nature
  - Severe: constant pain, numbness or sensory loss in the wrist and hand (specifically palm, index, or middle finger, or thumb), or thenar muscle atrophy
- Able to read and write in simplified Chinese (Mainland), understand and complete the questionnaire, and should provide informed consent.

Exclusion criteria

- CTS secondary to wrist deformity, trauma, mass, pregnancy, hypothyroidism, or inflammatory arthropathy;
- Treatment by night splint, therapeutic ultrasound or injection within the past 6 months, or previous carpal tunnel surgery;
- Previous surgery on the affected wrist (or study wrist if bilateral CTS);
- Unable to tolerate the study interventions;
- Trauma to the affected upper limb requiring operation or immobilization within the past 12 months;

For peer review only - http://bmjopen.bmj.com/site/about/guidelines.xhtml

- Current illness including, poorly controlled diabetes mellitus or thyroid disease, vibration-induced neuropathy, osteoarthritis or inflammatory joint disease, suspected complex neurological and musculoskeletal conditions;
- Known drugs or alcohol abuse;
- Allergy to any of the splint materials;
- Contraindications to therapeutic ultrasound, including dermatological conditions, abnormal sensation in the affected arm, indwelling electrical pumps/pacemakers, epilepsy, pregnancy or breastfeeding.

Following the medical evaluation, a research assistant will meet with the eligible participants and obtain their written informed consent. Demographic variables such as age, sex, body mass index, affected wrist (whether bilateral), dominant arm, lifestyle (smoking and alcohol use), and medical history of all participants will be collected prior to treatment (baseline). Participants will also be asked relevant questions about symptoms duration and previous treatments (rehabilitation exercises, injections or others). Others like occupation, employment characteristics (full-time or part-time work, manual or non-manual labor), employment status (whether on sickness absence), and professional activity characteristics (repetitive movements for >4 hours/day; wrist flexion and extension for >2 hours/day and use of computer keyboard/ mouse [how many hours/day]) will be also collected.

Randomization and blinding

Participants will be randomized in three intervention groups (either therapeutic ultrasound or night splint or therapeutic ultrasound + night splint arm) in a ratio of 1:1:1, using a computer-generated randomized sequence with varying unknown block sizes (either 3 or 6) for all study centres, without stratification. A research assistant who are not involved in clinical care and participant evaluations will prepare

For peer review only - http://bmjopen.bmj.com/site/about/guidelines.xhtml

sequentially numbered, opaque, sealed envelopes based on a random list, and ensure that the allocation data will not be accessed or influenced by anyone. When appropriate, the assistant will open envelopes and ensure coordination of therapeutic interventions.

The outcome assessor and statistician will be blinded to group allocation and not involved in treatment procedures.

Intervention

At the beginning, all participants will participate in an about 30-minute group educational presentation by a research assistant on the same day as the baseline assessment. This presentation will cover the pathophysiology, treatment options, posture and activity modification principles of CTS. The above information will also be provided to participants in the form of education booklets, to encourage them to review at home. Habits changes include limited wrist movement and a reduction in strenuous work activities, and the use of ergonomically friendly work tools helps reduce median nerve pressure.[32,33]

Participants in the [therapeutic ultrasound group] will receive pulsed therapeutic ultrasound (model 1:4, Shanghai, China) for 6 weeks at a frequency of 1 MHz and intensity of 1.0 W/cm^2 for 15 minutes per session, in daily 5 times a week for the first 2 weeks and twice a week for another 4 weeks, to the area over the carpal tunnel, referred to a published trial [BMJ. 1998;316:731-735].

Participants allocated to the [night splint group] will receive a splint to wear at night for 6 weeks, referred to a published trial [Lancet. 2018;392:1423-1433]. The splint holds the wrist in a neutral position or slightly extended 20° from the neutral position to avoid wrist movement, which can increase pressure on the carpal tunnel.[34] The choose of each splint will be based on the size of individual participant's hands

For peer review only - http://bmjopen.bmj.com/site/about/guidelines.xhtml

and arms. Participants will be shown how to fit and remove the wrist splint according to a standardized trial protocol. Oral guidance from the clinician on how and when to use splints will encourage and reinforce compliance, which will be also supported by written information, detailed care, and splint fitting and use. Participants will be instructed to perform gentle range-of-motion exercises when removing the splint to prevent stiffness.

Participants randomized to the [therapeutic ultrasound + night splint group] will receive both therapeutic ultrasound for 6 weeks as in the [therapeutic ultrasound group] and night splint for 6 weeks as in the [night splint group].

For participants with bilateral CTS, the non-study hand will be treated according to normal clinical protocols in use at the research site.

We discouraged additional treatments to that assigned (that is, not per protocol) during the intervention period, but we allowed the use of simple analgesics as needed. Participants reported all not per protocol treatments, such as drugs, in a diary.

Data management

Data will be collected during the participants' visits to the hospital at baseline, 6 weeks, 3 months, 6 months, and one-year after random assignment (**Table 1**). Reminder emails and phone calls from the research assistants will be programmed to maximize participant compliance in subsequent completion. A registered participant will withdraw from the study if (1) the participant withdraws his/her consent, and (2) exclusion criteria is found after registration. The cause and date of suspension will be recorded. Consent to use data that has been collected before the participant's withdrawal will be included in the consent form.

Primary outcome measure

For peer review only - http://bmjopen.bmj.com/site/about/guidelines.xhtml

The primary outcome measure will be the difference in Symptom Severity Scale of the Boston Carpal Tunnel Questionnaire (BCTQ-SSS) at 6-week. The BCTQ is a disease-specific questionnaire referring to a typical 24-hour period in the past two weeks,[35] and has been shown to be highly reproducible, internally consistent, valid and responsive to clinical change in CTS.[36] It consists of two different subscales: Symptom Severity Scale (11 items, about symptom severity) and Functional Status Scale (8 items, about the degrees of difficulty on daily activities), both rated on a five-point scale, with final scores for each subscale result in mean scores between 1 and 5. The overall score is calculated as the mean of all 19 items. Higher scores represent more severe symptoms and functional impairment. We use a validated Chinese version[37] of the BCTQ in this study.

Secondary outcome

Secondary outcome measures will be the differences in Functional Status Scale of the Boston Carpal Tunnel Questionnaire (BCTQ-FSS), sleep questionnaire for interrupted sleep[38], EuroQol-5D (EQ-5D)[39] for life quality and health status, Hospital Anxiety and Depression Scale (HADS)[40] for anxiety and depression status, Work Limitations Questionnaire (WLQ)-25[41] for functional limitations at work, Global Rating of Change (GROC) for treatment success and recurrence rate, physical function examination as well as various electrophysiology and ultrasound parameters.

■ Interrupted sleep

The sleep questionnaire, which will be used to assess sleep quality, consists of four questions asking participants how many times they have experienced it in the last month.[38] Each question has six response categories and are coded in 0-5 order: not at all, 1-3, 4-7, 8-14, 15-21 and 22-31 days. All questions have equal weights and add up. Higher scores are associated with more disrupted sleep.

For peer review only - http://bmjopen.bmj.com/site/about/guidelines.xhtml

■ Life quality and health status

EQ-5D has been widely validated and used to measure generic health-related quality of life (HRQol) due to its simplicity.[39] It consists of a five-dimensional description system (mobility, self-care, usual activities, pain/discomfort and anxiety/depression) and a VAS that scales from 0 to 1, where 1 represents perfect health. All dimensions are divided into three levels (no problem, some problem and extreme problem). We used a validated Chinese version[42,43] of EQ-5D, which has been recommended by Guidelines for Pharmacoeconomic Evaluations 2011 for a measure for HRQol and health utility.[44]

■ Anxiety and depression status

HADS will be used to identify and quantify two of the most common psychological disorders - anxiety and depression.[40] There is evidence of increased anxiety and depression in LET patients.[45] The HADS is a 14-item scale independent of physical symptoms and consists of two 7-item subscales measuring depression and anxiety, respectively. A 4-point scale (0 for absence of symptoms, and 3 for maximum symptomatology) is used. Each subscale has an overall score ranging from 0 to 21, with a higher score indicating a higher degree of impairment. HADS has two cut offs for categorization: 0-7, "non-case"; 8-10, "possible or doubtful case"; 11-21, "probable or definite case".[46]

■ Functional limitations at work

To gather information that is complementary to the pain and disability scales, functional limitations at work will be measured using WLQ-25. It contains 25 items, arranged under four sub-scales, covering four dimensions of job demands, namely time demands, physical demands, mental/interpersonal demands, and output demands.[41] A five-level ordinal response scale ranging from 0 (all of the time) to 4

For peer review only - http://bmjopen.bmj.com/site/about/guidelines.xhtml

(none of the time) with an additional sixth option (does not apply to my job) is used. The overall score ranges from 0 to 100, with an increase of 13 points (out of 100) for clinically important differences.[47]

■ Treatment success and recurrence rate

Participants' treatment impressions of changes in their condition (ranging from “completely recovered”, “much improved”, “somewhat improved”, “same”, “worse” to “much worse”) will be recorded on a six-level Likert scale. The success rate will be calculated by dichotomizing the response. Participants who report " completely recovered " or " much improved " in their overall condition since the study beginning will be considered successful, while other responses will be considered failures.[48,49] Recurrence will be defined primarily as when a participant rates a success at 6 weeks and a failure at 3 months, 6 months or one-year on GROC.[48,49]

■ Physical function examination

The physical examinations will include measurement of 2-point discrimination (performed on the radial and ulnar aspects of each digit), grip strength with a dynamometer (CAMRY, City of Industry, CA, USA), and pinch strength with the pinch gauge (three trials for each hand). The affected side will be measured first and then the unaffected side. The measurement readings will be not revealed to the subjects until the completion of the test. The mean of three consecutive trials, separated by a 20s pause, will be calculated. Results will be presented as a ratio of values of the symptomatic side/ asymptomatic side×100.[50]

The 2-point discrimination test starts at a distance of 4 mm and increases continuously by 2 mm as necessary. Grip strength and 3-point pinching force (3 tests per hand) as measured with baseline dynamometer and pinch gauge (Chattanooga Group, Hixson, Tennessee, USA) will be recorded.

For peer review only - http://bmjopen.bmj.com/site/about/guidelines.xhtml

■ Electrophysiological Study

Median nerve distal motor latency (DML), compound muscle action potential (CMAP), sensory nerve conduction velocity (SNCV) and sensory nerve action potential (SNAP) amplitudes will be recorded.[48,49] DML and CMAP will be measured by placing surface electrodes on the abductor pollicis brevis muscle, and stimulation applied 8 cm proximal to the active recording electrode. SNAP and SNCV will be obtained using ring electrodes placed on the proximal and distal interphalangeal joints of the index finger. Sensory conduction will be studied by antidromically stimulation at 14 cm proximal to the active electrode. Motor study will be performed by supramaximal stimulation while the amplitude will be measured an average of 10 times for the sensory study. All measurements will be made 3 times, and the values obtained will be averaged for analysis.

■ Ultrasound parameters

The cross-sectional area (CSA) will be measured using an electronic caliper at the scaphoid-pisiform level.[51,52] The measurements will be made 3 times, and the values obtained will be averaged for analysis.

Adverse events

All adverse events, defined as any negative or unwanted reactions to intervention, will be recorded through the symptoms reported by the patients, and observations by a researcher at every visit. Therapeutic ultrasound may cause mild local swelling, spot-like bleeding, ecchymosis, enhanced local pain response, and local hyperesthesia or decrease. Night splint may cause skin allergy, wrist stiffness, et al. The participants will be instructed to do gentle range-of-motion exercises when removing the splint to prevent stiffness and reinforced adherence by verbal instruction.

For peer review only - http://bmjopen.bmj.com/site/about/guidelines.xhtml

Sample size calculation

Sample size and power calculation are based on the primary outcome of BCTQ-SSS score at 6-week. All sample size calculations assume two-sided analysis with a power of 90% (1-β=0.90) at a significant level of α=0.05. Based on a published RCT trial ([Lancet. 2018;392:1423-1433], the authors compared corticosteroid injection and night splint for CTS, and participants in the night splint group had a standard deviation [SD] of 0.76 points for BCTQ-SSS at their 6-week followup, ["6-week" was the primary endpoint in this study]),[9] a SD of 0.76-point on BCTQ-SSS score will be used. To detect a minimum clinically significant difference of 0.8-point[53] (superiority margin) between therapeutic ultrasound + night splint and night splint groups (assuming a true difference of 1.19-point[9,54]), a total of 48 participants in each group is required. Allowing for an up to 10% drop out rate, we aim to enroll at least 54 participants in each group to complete the study.

Analysis plan

Baseline characteristics of the three treatment groups will be summarized using appropriate descriptive statistics. Both the primary and secondary analyses will be blind analyses of treatment assignments and will be performed using the intention-to-treat (ITT)[55] method, with all randomized participants retaining their original assigned group. Multiple imputation by chained equations will be used to address missing data caused by lost access and non-response if these missing data are judged to be random.

The primary comparisons for BCTQ-SSS scores will be made using linear regression. In secondary analyses, repeated measures mixed model[56] will also be used to examine the associations between treatments and repeated outcome measures, with terms of treatment, time, trial center and corresponding baseline values as covariates (age, gender, body mass index, et al.). Bonferroni method will be used to adjust for

For peer review only - http://bmjopen.bmj.com/site/about/guidelines.xhtml

multiplicity.[57,58] Linear regression will be used for numerical outcomes, and logistic/ordinal regression for any categorical outcomes.

Quality assurance/monitoring/management

In order to standardize the procedures of staff training and learning, such as participants recruitment, outcome measures, data import, security, management and analysis, a Manual of Operations and Procedures and a case report form will be developed as per protocol, which also include the monitoring plans to assure participant protection and data integrity, thus facilitating consistency in protocol implementation and data collection. The investigators, physicians, research assistants, outcome assessors and statisticians are different people and should be trained in good clinical practice. Trained project managers will visit each centre for monitoring to ensure data quality and compliance with the trial protocol.

All data obtained will be stored electronically and strictly in a database with secure and restricted access. Encryption will be used for data transmission, with removal for any information that can identify individuals. Data will only be de-identified for analysis at the completion of this study.

Study duration

Recruitment of the trial will begin in the November of 2021 and one-year follow-up for all participants is anticipated to be completed by June 2023. See **Table 1** for time points and recruitment progress.

Ethics and dissemination

This study has been approved by the Ethics Committee of Shanghai Sixth People's Hospital (lead Clinical Center, approval No. 2021-152), Ethics Committee of Shanghai East Hospital (EC.D(BG).016.03.1-2021-095), Ethics Committee of Shanghai Tenth People's Hospital (SHSY-IEC-4.1/21-194/01), and Ethics Committee

For peer review only - http://bmjopen.bmj.com/site/about/guidelines.xhtml

of Pudong New Area People's Hospital (IRBY2021-006). The potential risks of this clinical trial are considered minimal and are addressed in the protocol and consent form. A written consent (**Supplemental file 1**) will be obtained by clinical practitioners from each participant. The trial was registered on www.chictr.org website (registration number ChiCTR2100050701). Data will be published in peer-reviewed journals and presented at conferences, both nationally and internationally.

For peer review only

For peer review only - http://bmjopen.bmj.com/site/about/guidelines.xhtml

DISCUSSION

CTS is a highly prevalent compression neuropathy, which results in significant paraesthesia and numbness in the median nerve distribution, especially nocturnal symptoms causing sleep disturbance, causing great socioeconomic burden. Up till now, there is still no consensus on the optimal management, and nonoperative treatment is generally accepted as the first-line intervention for mild and moderate CTS. Multiple methods have been studied and reviewed in the recent decades, however, the exact efficacy still remains controversial.

In an RCT published in BMJ for mild to moderate CTS, active therapeutic ultrasound (1 MHz, 1.0 W/cm^2) was applied to the area over the carpal tunnel in the experimental group, and indistinguishable sham US treatment was applied in the control group.[23] At 6 months' follow up, satisfactory improvement or complete remission of symptoms was observed in 74% receiving active treatment, which is significantly higher than those receiving sham treatment (20%). As for electroneurography, DML and SNCV improved significantly with active treatment while remained unchanged with sham treatment. Hand grip and finger pinch strength in physical examination also improved significantly with active treatment. All results suggested satisfying effects from therapeutic ultrasound for CTS.

Therapeutic ultrasound can also be used as part of a multi-intervention approach. Some studies have compared night splint alone to night splint combined with therapeutic ultrasound in treatment of CTS, while the effects were different, and the grades of study design and data were low. Dincer U et al.[26] found that the improvements in the combined group were statistically significantly better ($p=0.043$) than those in night splint alone group in BCTQ-SSS, as well as BCTQ-FSS ($p<0.001$), and VAS for pain ($p<0.001$). Similar results were also reported by Baysal

For peer review only - http://bmjopen.bmj.com/site/about/guidelines.xhtml

O et al.[59]; while Jothi KP et al.[27], Sim SE et al.[28] and Armagan O et al.[29] found therapeutic ultrasound may add no benefit to splinting in CTS.

In this study, to the best of our knowledge, it is the first to compared the efficacy between therapeutic ultrasound [therapeutic ultrasound group] and night splint [night splint group] in CTS treatment. What's more, the additional effects of therapeutic ultrasound [therapeutic ultrasound + night splint group] in a multi-intervention approach will be compared with night splint alone [night splint group]. In clinic, therapeutic ultrasound is less invasive, less expensive, safer and more portable than other nonoperative therapy like drug injections for compression neuropathy and, if proved to be effective, could be offered to selected patients as part of non-operative therapy.

There are some on-going clinical trials on CTS treatment recent years,[60-63] and our prospective randomized study proposes to complement and add to this relevant and much needed scientific effort.

For peer review only - http://bmjopen.bmj.com/site/about/guidelines.xhtml

REFERENCES

1. Seiler JG, 3rd, Daruwalla JH, Payne SH, et al. Normal Palmar Anatomy and Variations That Impact Median Nerve Decompression. J Am Acad Orthop Surg. 2017;25(9):e194-e203.
2. Kleopa KA. In the Clinic. Carpal Tunnel Syndrome. Ann Intern Med. 2015;163-5:Itc1.
3. Feng B, Chen K, Zhu X, et al. Prevalence and risk factors of self-reported wrist and hand symptoms and clinically confirmed carpal tunnel syndrome among office workers in China: a cross-sectional study. BMC Public Health. 2021;21(1):57.
4. Latinovic R, Gulliford MC, Hughes RA. Incidence of common compressive neuropathies in primary care. J Neurol Neurosurg Psychiatry. 2006;77(2):263-5.
5. Jerosch-Herold C, Houghton J, Blake J, et al. Association of psychological distress, quality of life and costs with carpal tunnel syndrome severity: a cross-sectional analysis of the PALMS cohort. BMJ Open. 2017;7(11):e017732.
6. Werner RA, Franzblau A, Gell N. Randomized controlled trial of nocturnal splinting for active workers with symptoms of carpal tunnel syndrome. Arch Phys Med Rehabil. 2005;86(1):1-7.
7. Padua L, Coraci D, Erra C, et al. Carpal tunnel syndrome: clinical features, diagnosis, and management. Lancet Neurol. 2016;15(12):1273-84.
8. Graham B. The value added by electrodiagnostic testing in the diagnosis of carpal tunnel syndrome. J Bone Joint Surg Am. 2008;90(12):2587-93.
9. Chesterton LS, Blagojevic-Bucknall M, Burton C, et al. The clinical and cost-effectiveness of corticosteroid injection versus night splints for carpal tunnel

For peer review only - http://bmjopen.bmj.com/site/about/guidelines.xhtml

syndrome (INSTINCTS trial): an open-label, parallel group, randomised controlled trial. Lancet. 2018;392(10156):1423-33.

10. Jiménez Del Barrio S, Estébanez de Miguel E, Bueno Gracia E, et al. Effects of diacutaneous fibrolysis in patients with mild to moderate symptomatic carpal tunnel syndrome: a randomized controlled trial. Clin Rehabil. 2018;32(12):1645-55.

11. Huisstede BM, Fridén J, Coert JH, et al. Carpal tunnel syndrome: hand surgeons, hand therapists, and physical medicine and rehabilitation physicians agree on a multidisciplinary treatment guideline—results from the European HANDGUIDE Study. Arch Phys Med Rehabil. 2014;95(12):2253-63.

12. Carpal Tunnel Syndrome: A Summary of Clinical Practice Guideline Recommendations-Using the Evidence to Guide Physical Therapist Practice. J Orthop Sports Phys Ther. 2019;49(5):359-60.

13. Graham B, Peljovich AE, Afra R, et al. The American Academy of Orthopaedic Surgeons Evidence-Based Clinical Practice Guideline on: Management of Carpal Tunnel Syndrome. J Bone Joint Surg Am. 2016;98(20):1750-4.

14. Choi GH, Wieland LS, Lee H, et al. Acupuncture and related interventions for the treatment of symptoms associated with carpal tunnel syndrome. Cochrane Database Syst Rev. 2018;12(12):Cd011215.

15. Page MJ, O'Connor D, Pitt V, et al. Exercise and mobilisation interventions for carpal tunnel syndrome. Cochrane Database Syst Rev. 2012(6):Cd009899.

16. Franke TP, Koes BW, Geelen SJ, et al. Do Patients With Carpal Tunnel Syndrome Benefit From Low-Level Laser Therapy? A Systematic Review of Randomized Controlled Trials. Arch Phys Med Rehabil. 2018;99(8):1650-9.e15.

For peer review only

For peer review only - http://bmjopen.bmj.com/site/about/guidelines.xhtml

17. Kim JC, Jung SH, Lee SU, et al. Effect of extracorporeal shockwave therapy on carpal tunnel syndrome: A systematic review and meta-analysis of randomized controlled trials. Medicine (Baltimore). 2019;98(33):e16870.

18. Catapano M, Catapano J, Borschel G, et al. Effectiveness of Platelet-Rich Plasma Injections for Nonsurgical Management of Carpal Tunnel Syndrome: A Systematic Review and Meta-analysis of Randomized Controlled Trials. Arch Phys Med Rehabil. 2020;101(5):897-906.

19. O'Brien WD, Jr. Ultrasound-biophysics mechanisms. Prog Biophys Mol Biol. 2007;93-1(3):212-55.

20. Schuhfried O, Vukanovic D, Kollmann C, et al. Effects of Pulsed Ultrasound Therapy on Sensory Nerve Conduction Parameters and the Pain Threshold Perceptions in Humans. Pm r. 2017;9(8):781-6.

21. Ilham SJ, Chen L, Guo T, et al. In vitro single-unit recordings reveal increased peripheral nerve conduction velocity by focused pulsed ultrasound. Biomed Phys Eng Express. 2018;4(4).

22. de Oliveira Perrucini PD, Poli-Frederico RC, de Almeida Pires-Oliveira DA, et al. Anti-Inflammatory and Healing Effects of Pulsed Ultrasound Therapy on Fibroblasts. Am J Phys Med Rehabil 2020;99(1):19-25.

23. Ebenbichler GR, Resch KL, Nicolakis P, et al. Ultrasound treatment for treating the carpal tunnel syndrome: randomised "sham" controlled trial. BMJ. 1998;316(7133):731-5.

24. Page MJ, O'Connor D, Pitt V, et al. Therapeutic ultrasound for carpal tunnel syndrome. Cochrane Database Syst Rev. 2013;2013(3):Cd009601.

25. Huisstede BM, Hoogvliet P, Franke TP, et al. Carpal Tunnel Syndrome: Effectiveness of Physical Therapy and Electrophysical Modalities. An Updated

For peer review only - http://bmjopen.bmj.com/site/about/guidelines.xhtml

Systematic Review of Randomized Controlled Trials. Arch Phys Med Rehabil. 2018;99(8):1623-34.e23.

26. Dincer U, Cakar E, Kiralp MZ, et al. The effectiveness of conservative treatments of carpal tunnel syndrome: splinting, ultrasound, and low-level laser therapies. Photomed Laser Surg. 2009;27(1):119-25.

27. Jothi KP, Bland JDP. Ultrasound therapy adds no benefit to splinting in carpal tunnel syndrome. Muscle Nerve. 2019;60(5):538-43.

28. Sim SE, Gunasagaran J, Goh KJ, et al. Short-term clinical outcome of orthosis alone vs combination of orthosis, nerve, and tendon gliding exercises and ultrasound therapy for treatment of carpal tunnel syndrome. J Hand Ther. 2019;32(4):411-6.

29. Armagan O, Bakilan F, Ozgen M, et al. Effects of placebo-controlled continuous and pulsed ultrasound treatments on carpal tunnel syndrome: a randomized trial. Clinics (Sao Paulo). 2014;69(8):524-8.

30. Chan AW, Tetzlaff JM, Gøtzsche PC, et al. SPIRIT 2013 explanation and elaboration: guidance for protocols of clinical trials. BMJ. 2013;346:e7586.

31. Burton C, Chesterton LS, Davenport G. Diagnosing and managing carpal tunnel syndrome in primary care. Br J Gen Pract. 2014;64(622):262-3.

32. O'Connor D, Page MJ, Marshall SC, et al. Ergonomic positioning or equipment for treating carpal tunnel syndrome. Cochrane Database Syst Rev. 2012;1(1):Cd009600.

33. Buchan S, Amirfeyz R. Cochrane corner: ergonomic positioning or equipment for treating carpal tunnel syndrome. J Hand Surg Eur Vol. 2013;38(5):580-1.

For peer review only

34. Weiss ND, Gordon L, Bloom T, et al. Position of the wrist associated with the lowest carpal-tunnel pressure: implications for splint design. J Bone Joint Surg Am. 1995;77(11):1695-9.

35. Levine DW, Simmons BP, Koris MJ, et al. A self-administered questionnaire for the assessment of severity of symptoms and functional status in carpal tunnel syndrome. J Bone Joint Surg Am. 1993;75(11):1585-92.

36. Leite JC, Jerosch-Herold C, Song F. A systematic review of the psychometric properties of the Boston Carpal Tunnel Questionnaire. BMC Musculoskelet Disord. 2006;7:78.

37. Lue YJ, Lu YM, Lin GT, et al. Validation of the Chinese version of the Boston Carpal Tunnel Questionnaire. J Occup Rehabil. 2014;24(1):139-45.

38. Jenkins CD, Stanton BA, Niemcryk SJ, et al. A scale for the estimation of sleep problems in clinical research. J Clin Epidemiol. 1988;41(4):313-21.

39. EuroQol--a new facility for the measurement of health-related quality of life. Health Policy. 1990;16(3):199-208.

40. Zigmond AS, Snaith RP. The hospital anxiety and depression scale. Acta Psychiatr Scand. 1983;67(6):361-70.

41. Lerner D, Amick BC 3rd, Rogers WH, et al. The Work Limitations Questionnaire. Med Care. 2001;39(1):72-85.

42. Wu C, Gong Y, Wu J, et al. Chinese Version of the EQ-5D Preference Weights: Applicability in a Chinese General Population. PLoS One. 2016;11(10):e0164334.

43. Luo N, Liu G, Li M, et al. Estimating an EQ-5D-5L Value Set for China. Value Health. 2017;20(4):662-9.

For peer review only

For peer review only - http://bmjopen.bmj.com/site/about/guidelines.xhtml

44. Sun S, Chen J, Johannesson M, et al. Population health status in China: EQ-5D results, by age, sex and socio-economic status, from the National Health Services Survey 2008. Qual Life Res. 2011;20(3):309-20.

45. Alizadehkhaiyat O, Fisher AC, Kemp GJ, et al. Pain, functional disability, and psychologic status in tennis elbow. Clin J Pain. 2007;23(6):482-9.

46. Pallant JF, Bailey CM. Assessment of the structure of the Hospital Anxiety and Depression Scale in musculoskeletal patients. Health Qual Life Outcomes. 2005;3:82.

47. Roy JS, MacDermid JC, Amick BC 3rd, et al. Validity and responsiveness of presenteeism scales in chronic work-related upper-extremity disorders. Phys Ther. 2011;91(2):254-66.

48. Wang JC, Liao KK, Lin KP, et al. Efficacy of Combined Ultrasound-Guided Steroid Injection and Splinting in Patients With Carpal Tunnel Syndrome: A Randomized Controlled Trial. Arch Phys Med Rehabil 2017;98(5):947-56.

49. Hsu PC, Liao KK, Lin KP, et al. Comparison of Corticosteroid Injection Dosages in Mild to Moderate Idiopathic Carpal Tunnel Syndrome: A Randomized Controlled Trial. Arch Phys Med Rehabil. 2020;101(11):1857-64.

50. Smidt N, van der Windt DA, Assendelft WJ, et al. Interobserver reproducibility of the assessment of severity of complaints, grip strength, and pressure pain threshold in patients with lateral epicondylitis. Arch Phys Med Rehabil. 2002;83(8):1145-50.

51. Wu YT, Ke MJ, Ho TY, et al. Randomized double-blinded clinical trial of 5% dextrose versus triamcinolone injection for carpal tunnel syndrome patients. Ann Neurol. 2018;84(4):601-610.

For peer review only

For peer review only - http://bmjopen.bmj.com/site/about/guidelines.xhtml

52. Wu YT, Ho TY, Chou YC, et al. Six-month Efficacy of Perineural Dextrose for Carpal Tunnel Syndrome: A Prospective, Randomized, Double-Blind, Controlled Trial. Mayo Clin Proc. 2017;92(8):1179-1189.

53. Chesterton LS, Dziedzic KS, van der Windt DA, et al. The clinical and cost effectiveness of steroid injection compared with night splints for carpal tunnel syndrome: the INSTINCTS randomised clinical trial study protocol. BMC Musculoskelet Disord. 2016;17(1):415.

54. Boonhong J, Thienkul W. Effectiveness of Phonophoresis Treatment in Carpal Tunnel Syndrome: A Randomized Double-blind, Controlled Trial. PM R. 2020;12(1):8-15.

55. Sedgwick P. Intention to treat analysis versus per protocol analysis of trial data. BMJ. 2015;350:h681.

56. Detry MA, Ma Y. Analyzing Repeated Measurements Using Mixed Models. JAMA. 2016;315(4):407-8.

57. Thybo KH, Jakobsen JC, Hägi-Pedersen D, et al. PANSAID-PAracetamol and NSAID in combination: detailed statistical analysis plan for a randomised, blinded, parallel, four-group multicentre clinical trial. Trials. 2017;18(1):465.

58. Thybo KH, Hägi-Pedersen D, Dahl JB, et al. Effect of Combination of Paracetamol (Acetaminophen) and Ibuprofen vs Either Alone on Patient-Controlled Morphine Consumption in the First 24 Hours After Total Hip Arthroplasty: The PANSAID Randomized Clinical Trial. JAMA. 2019;321(6):562-571.

59. Baysal O, Altay Z, Ozcan C, et al. Comparison of three conservative treatment protocols in carpal tunnel syndrome. Int J Clin Pract. 2006;60(7):820-8.

For peer review only

For peer review only - http://bmjopen.bmj.com/site/about/guidelines.xhtml

60. Atroshi I, Tadjerbashi K, McCabe SJ, et al. Treatment of carpal tunnel syndrome with wrist splinting: study protocol for a randomized placebo-controlled trial. Trials. 2019;20(1):531.

61. Chen CC, Wu YT, Su YC, et al. Efficacy of laser acupuncture for carpal tunnel syndrome: A study protocol for a prospective double-blind randomized controlled trial. Medicine (Baltimore). 2019;98(30):e16516.

62. Dimitrova A, Murchison C, Oken B. Local effects of acupuncture on the median and ulnar nerves in patients with carpal tunnel syndrome: a pilot mechanistic study protocol. Trials. 2019;20(1):8.

63. Mason W, Ryan D, Khan A, et al. Injection versus Decompression for Carpal Tunnel Syndrome-Pilot trial (INDICATE-P)-protocol for a randomised feasibility study. Pilot Feasibility Stud. 2017;3:20.

For peer review only - http://bmjopen.bmj.com/site/about/guidelines.xhtml

Figure Legends

Figure 1 Participant flow chart

For peer review only

For peer review only - http://bmjopen.bmj.com/site/about/guidelines.xhtml

Table 1 Study evaluation procedures and timeline

**Study procedure**	**Medical evaluation**	**Enrolment visit**	**6 weeks**	**3 months**	**6 months**	**One year**
Determine eligibility	√	√				
Obtain signed consent		√				
Obtain medical and demographic data		√				
Give instructions for Pain medication diary		√				
Outcome measures						
Boston Carpal Tunnel Questionnaire		√	√	√	√	√
Interrupted sleep questionnaire		√	√	√	√	√
EuroQol-5D		√	√	√	√	√
Hospital Anxiety and Depression Scale		√	√	√	√	√
Work Limitations Questionnaire-25		√	√	√	√	√
Treatment success rate			√	√	√	√
Treatment recurrence rate				√	√	√
Physical examination		√	√	√	√	√
Electrophysiological and ultrasound parameters		√	√	√	√	√

For peer review only

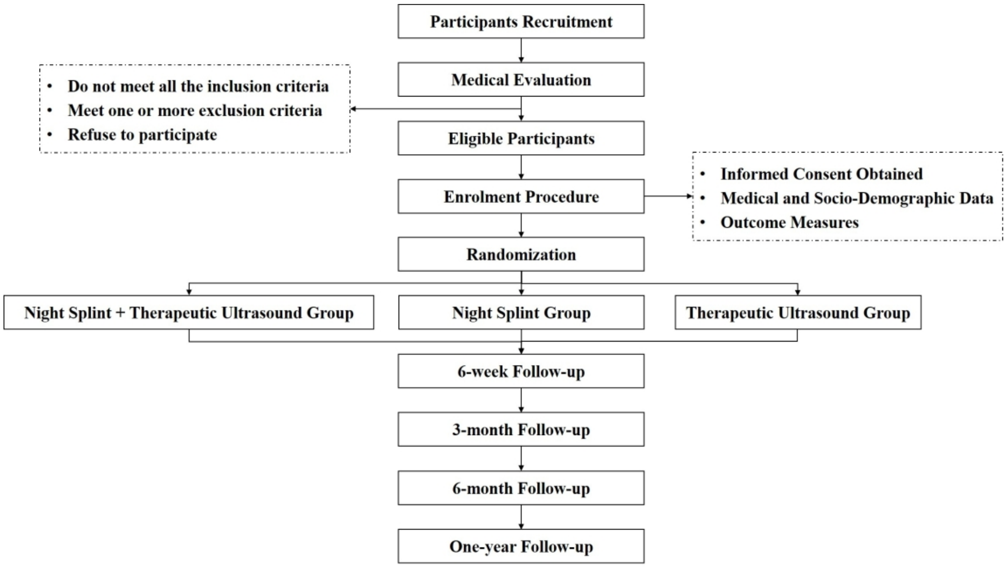

Figure 1 Participant flow chart

377x212mm (150 x 150 DPI)

INFORMED CONSENT FORM

(English Version)

Participant Information Page

**Study Title**	:	Effectiveness of ultrasound therapy for the treatment of carpal tunnel syndrome
**Principal Investigator**	:	Cunyi Fan
**Sponsor**	:	Shanghai Sixth People's Hospital

For peer review only

Dear participant:

You have been diagnosed with carpal tunnel syndrome, and will be invited to participate in the study named "Effectiveness of ultrasound therapy for the treatment of carpal tunnel syndrome". The study is conducted by the researchers themselves. Please read this informed consent carefully and make the decision whether to participate in this study or not. Participation in this study is entirely your choice. As a participant, you must give your written consent prior to joining the clinical study. When your doctor or researcher discusses informed consent with you, you can ask him or her to explain to you what you don't understand. We encourage you to discuss this thoroughly with your family and friends before making any decision to participate in this study. You have the right to refuse to participate in the study or withdraw from the study at any time without being penalized or losing your rights. If you are participating in another study, please inform your study doctor or investigator. The background, purpose, process and other important information of this study are as follows:

1. BACKGROUND

Carpal tunnel syndrome (CTS), the most common compression neuropathy, results from median nerve entrapment in the carpal tunnel, accounting for about 90% of all such disorders. The clinically confirmed CTS prevalence was 9.6% in the general population of China, with a yearly incidence rate of 2.76‰, and women is more susceptible than men. CTS has significant impact on daily life and ability to work, and causes great burden on social economy, with an annual associated cost estimated at $13 billion. Classically, CTS causes discomfort, paraesthesia and numbness in the median nerve distribution; and nocturnal symptoms are often clinically significant causing sleep disturbance. Patients can

For peer review only - http://bmjopen.bmj.com/site/about/guidelines.xhtml

be diagnosed by clinical history and physical examination; while electrophysiological methods will be additional for insufficient diagnosis by clinical findings and severe cases that need surgical management.

In general, the severity of CTS can be classified into mild, moderate and severe. Non-surgical interventions are suggested to be the first choice to treat mild and moderate CTS. To date, though the treatment method is vast; however, no successful and universally accepted regimen has been established. A consensus of multidisciplinary treatment guideline from the European HANDGUIDE Study suggests that "education" should be included as the first-line management approach, which has the advantages of low cost, high efficacy and non-invasiveness. In addition, "night splint (NS)" and "corticosteroid injection (CI)" are also recommended in guidelines of American Academy of Orthopaedic Surgeons (AAOS) and American Physical Therapy Association (APTA). One recent RCT published in Lancet compares both two methods, and finds that CI has superior clinical effectiveness at 6 weeks than NS, but no differences at 6 months; while CI may bring adverse events like thinning, lightening or darkening of the skin at the injection site, hot flushes and even more pain. Systematic reviews have also shown that the effects of other conservative treatments like acupuncture, exercise and mobilization interventions, laser, extracorporeal shockwave therapy and platelet-rich plasma injection still remain controversial or provide little to no benefit.

Ultrasound (US) is widely used for imaging purposes and regarded as an adjunct to physiotherapy. In the intensity range of 0.5-2.0 W/cm^2, US may have the potential to induce a variety of biophysical effects in tissues. US experiments on stimulation of nerve conduction and regeneration, and discoveries of its anti-inflammatory effects all support that US may promote recovery of nerve compression. An RCT published in BMJ showed more pronounced subjective symptoms and electroneurographic variables for US treatment than sham control in patients with mild to moderate CTS. However, to our best of knowledge, no study has compared the efficacy between NS and US in CTS treatment yet. Additionally, some studies have also reported the efficacy of US to be used as part of a multi-intervention approach, but with low grade of study design and data. Therefore, the role of US in CTS treatment still needs to be further explored by high-quality study.

2. STUDY PURPOSE

The purpose of the current three-arm, prospective, randomized, multicenter trial is to investigate the effectiveness of US in treatment for CTS, that is, NS+US (combined) versus NS versus US, on clinical and functional outcomes, including Boston Carpal Tunnel

For peer review only - http://bmjopen.bmj.com/site/about/guidelines.xhtml

Questionnaire (BCTQ).

3. STUDY PROCESS

(1) How many people will participate in the study?

About 162 people will participate in the study at 4 municipal tertiary hospitals: Shanghai Sixth People's Hospital (leader unit), Shanghai East Hospital (participating unit), Shanghai Tenth People’s Hospital (participating unit) and Pudong New Area People′s Hospital of Shanghai (participating unit).

(2) What are the study procedures?

Before you are enrolled in the study, your medical history will be asked, and you will be screened for carpal tunnel syndrome by using criteria developed from a consensus survey by the UK Primary Care Rheumatology Society.

After determining that you are eligible to participate in the study based on inclusion and exclusion criteria, you will be collected and randomly assigned to treatment:

A. Characteristic features collection

You will be asked for your age, sex, body mass index, affected wrist (whether bilateral), dominant arm, lifestyle (smoking and alcohol use), and medical history. As well as relevant questions about symptoms duration and previous treatments (rehabilitation exercises, injections or others). Others like occupation, employment characteristics (full-time or part-time work, manual or non-manual labor), employment status (whether on sickness absence), professional activity characteristics, and sports activities will be also collected.

B. Clinical features collection

You will complete the following questionnaires, including Boston Carpal Tunnel Questionnaire (BCTQ) for wrist function and symptom, sleep questionnaire for interrupted sleep, EuroQol-5D (EQ-5D) for life quality and health status, Hospital Anxiety and Depression Scale (HADS) for anxiety and depression status, Work Limitations Questionnaire (WLQ)-25 for functional limitations at work, Global Rating of Change (GROC) for treatment success and recurrence rate, as well as physical function examination and various electrophysiology and ultrasound parameters.

C. Treatment by group

At the beginning, all participants will participate in an about 30-minute group educational presentation by a research assistant on the same day as the baseline assessment. This presentation will cover the pathophysiology, treatment options, posture and activity

For peer review only - http://bmjopen.bmj.com/site/about/guidelines.xhtml

modification principles of CTS. The above information will also be provided to participants in the form of education booklets, to encourage them to review at home. Habits changes include limited wrist movement and a reduction in strenuous work activities, and the use of ergonomically friendly work tools helps reduce median nerve pressure.

You will be randomly assigned to one of three groups, [US group] vs. [NS group] vs. [US+NS group]:

(a) If you are assigned in the [US group], you will receive pulsed therapeutic ultrasound (model 1:4, Shanghai, China) for 6 weeks at a frequency of 1 MHz and intensity of 1.0 W/cm^2 for 15 minutes per session, in daily 5 times a week for the first 2 weeks and twice a week for another 4 weeks, to the area over the carpal tunnel, referred to a published trial [BMJ. 1998;316:731-735].

(b) If you are allocated to the [NS group], you will receive a splint to wear at night for 6 weeks, referred to a published trial [Lancet. 2018;392:1423-1433]. The splint holds the wrist in a neutral position or slightly extended 20° from the neutral position to avoid wrist movement, which can increase pressure on the carpal tunnel. The choose of each splint will be based on the size of each of your hands and arms. You will be shown how to fit and remove the wrist splint according to a standardized trial protocol. Oral guidance from the clinician on how and when to use splints will encourage and reinforce compliance, which will be also supported by written information, detailed care, and splint fitting and use. You will be instructed to perform gentle range-of-motion exercises when removing the splint to prevent stiffness.

(c) If you are randomized to the [US+NS group], you will receive both US for 6 weeks as in the [US group] and NS for 6 weeks as in the [NS group].

We discourage additional treatments to that assigned (that is, not per protocol) during the intervention period, but we allowed the use of simple analgesics as needed. You will report all not per protocol treatments, such as drugs, in a diary.

D. Follow-up features collection

Follow-up data will be collected during your visits to the hospital at 6 weeks, 3 and 6 months, and one year after random assignment.

(3) How long will the study last?

This study will continue for 1 year from the time you receive treatment, and we will collect follow-up information from you at 6 weeks, 3 months, 6 months, and one year at your regular outpatient review.

You may drop out of the study at any time without losing any benefits to which you are entitled. However, if you decide to withdraw during the study, you are encouraged to

For peer review only - http://bmjopen.bmj.com/site/about/guidelines.xhtml

talk to your doctor first. If you experience a serious adverse event, or if your study doctor feels it is not in your best interest to continue in the study, he or she may decide to withdraw you from the study. The sponsor or regulatory agency may also terminate during the study period. However, your withdrawal will not affect your normal medical treatment and rights.

If you withdraw from the study for any reason, you may be asked about your participation in the study. You may also be asked for a medical examination and follow-up questionnaire if your doctor deems it necessary.

(4) Information and biological specimens collected during the study

Biological specimens are not involved in this study, and the information collected is basic characteristics features, preoperative and follow-up clinical features (see the study procedures for details).

All data obtained will be kept strict and stored electronically on a database with secured and restricted access. An encryption will be used for data transfer, with removal for any information able to identify individuals. Data will be only deidentified for analysis at the completion of this study.

4. RISKS AND BENEFITS

(1) What are the risks of participating in this study?

The risks you may incur by participating in this study are as follows. You should discuss these risks with your study doctor or, if you prefer, with your regular care provider.

US treatment may cause mild local swelling, spot-like bleeding, ecchymosis, enhanced local pain response, and local hyperesthesia or decrease. The occurrence of these reactions depends on the dose of treatment, the extent of the lesion, and the individual patient, and usually does not require special treatment. Severe adverse reactions can be treated locally, or prolong the interval of treatment, reduce the intensity of treatment. If the treatment does not improve or abnormal conditions occur, the treatment should be stopped and immediately go to the hospital.

NS treatment may cause skin allergy, wrist stiffness, et al. You will be instructed to do gentle range-of-motion exercises when removing the splint to prevent stiffness and reinforced adherence by verbal instruction.

If you experience any discomfort, new changes, or any unexpected conditions during the study period, whether or not related to the study, you should inform your doctor in a timely manner, and he/she will judge and administer appropriate medical treatment.

During the study period, you need to visit the hospital on time and do some

For peer review only - http://bmjopen.bmj.com/site/about/guidelines.xhtml

examinations, which will take up some of your time and may cause trouble or inconvenience to you.

(2) What are the benefits of participating in the study?

If you agree to participate in this study, you may receive direct medical benefits, such as accelerated relief of symptoms of CTS. You can also have a deeper understanding of diseases and so on. In addition, we hope that the information gained from your participation in this study will benefit you or other patients with similar conditions in the future.

5. ALTERNATIVE TREATMENT OPTIONS

In addition to participating in this study, you may receive the other treatments provided by your doctor: corticosteroid injection, acupuncture, exercise and mobilization interventions, laser, extracorporeal shockwave therapy and platelet-rich plasma injection, and surgery, etc.

Please discuss these and other possible options with your doctor.

In general, the severity of CTS can be classified into mild, moderate and severe. Non-surgical interventions are suggested to be the first choice to treat mild and moderate CTS. To date, though the treatment method is vast; however, no successful and universally accepted regimen has been established. A consensus of multidisciplinary treatment guideline from the European HANDGUIDE Study suggests that “education” should be included as the first-line management approach, which has the advantages of low cost, high efficacy and non-invasiveness. In addition, “night splint (NS)” and “corticosteroid injection (CI)” are also recommended in guidelines of American Academy of Orthopaedic Surgeons (AAOS) and American Physical Therapy Association (APTA). One recent RCT published in Lancet compares both two methods, and finds that CI has superior clinical effectiveness at 6 weeks than NS, but no differences at 6 months; while CI may bring adverse events like thinning, lightening or darkening of the skin at the injection site, hot flushes and even more pain. Systematic reviews have also shown that the effects of other conservative treatments like acupuncture, exercise and mobilization interventions, laser, extracorporeal shockwave therapy and platelet-rich plasma injection still remain controversial or provide little to no benefit.

6. USE OF RESEACH RESULTS AND CONFIDENTIALITY OF PERSONAL INFORMATION

For peer review only - http://bmjopen.bmj.com/site/about/guidelines.xhtml

Results conducted through this program may be published in medical journals with the understanding and assistance of you and other participants, but we will keep your study records confidential as required by law.

The personal information of study participants will be kept strictly confidential, and your personal information will not be disclosed unless required by relevant laws.

If necessary, government administrative departments, hospital ethics committees and other relevant researchers can access your data according to regulations.

7. RESEARCH EXPENSES AND RELATED COPENSATION

(1) Cost of drugs/instruments used in the study and related examinations

There are no potential additional costs for this study. Routine outpatient fees include registration, examination for CTS, oral non-steroidal anti-inflammatory drugs, etc. The expenses related to US and NS will be borne by our research group and funding. In addition, you will be solely responsible for the expenses incurred by you for any treatment other than this study, as well as for the routine treatment and examination required for any concurrent disease.

(2) Compensation for participation in the study

There are no additional compensation costs for this study.

(3) Compensation/compensation after damage

For participants who suffer damage related to this study, the sponsor Shanghai Sixth People's Hospital will bear the treatment cost and corresponding economic compensation in accordance with Chinese laws and regulations.

8. RIGHTS OF PARTICIPANTS AND RELEVANT MATTERS NEEDING ATTENTION

(1) Your rights

Your participation in the study is voluntary throughout the entire process.

If you decide not to participate in this study, it will not affect other treatments you should receive.

If you decide to participate, you will be asked to sign this written informed consent. You have the right to withdraw from the trial at any stage without discrimination or unfair treatment, and your medical treatment and rights will not be affected.

For peer review only - http://bmjopen.bmj.com/site/about/guidelines.xhtml

(2) Matters needing attention

As a subject, you are required to provide true information about your medical history and current medical condition;

Inform the study doctor of any discomfort observed during the study;

Do not take any restricted drugs, food, etc. as advised by your doctor;

Tell the study doctor if you have recently participated in or are currently participating in other studies.

During the intervention, we discouraged additional therapy (i.e., not according to the grouping protocol), but we permitted the use of analgesics when needed (only acetaminophen and NSAIDs).

For medications taken, the name, dose, frequency and duration will be recorded at all follow-up visits.

9. RELEVANT CONTACT INFORMATION

If there is any significant new information during the study that may affect your willingness to continue to participate, your doctor will inform you promptly. If you are interested in your own study data, or you would like to know the findings after this study, you may ask any questions about this study at any time and receive answers accordingly, Please contact doctor Shuai Chen at ***********.

For peer review only - http://bmjopen.bmj.com/site/about/guidelines.xhtml

Participant Signature Page

Informed Consent Statement:

I have been informed of the purpose, background, process, risks and benefits of this study. I have plenty of time and opportunity to ask questions, and I am satisfied with the answers.

I am also told who to contact when I have questions, want to report difficulties, concerns, suggestions for research, or want further information, or to help with research.

I have read this informed consent and agree to participate in this study.

I understand that I may choose not to participate in the study or withdraw from the study at any time during the study without any reason.

I already know that if I get worse, or if I have a serious adverse event, or if my study doctor decides it's not in my best interest to continue, he or she will decide to withdraw me from the study. The funder or regulatory agency may terminate during the study without my consent. If this happens, the doctor will inform me and the study doctor will discuss other options with me.

I will be provided with a copy of the informed consent which contains my signature and that of the investigator.

Participant Signature: _______________
Date: _______________
(NOTE: If participant has no capacity/limited capacity, legal representative signature and date will be required)

Legal Representative's Signature: _______________
Date: _______________

Investigator Signature: _______________
Date: _______________

For peer review only - http://bmjopen.bmj.com/site/about/guidelines.xhtml

Reporting checklist for protocol of a clinical trial.

Based on the SPIRIT guidelines.

Instructions to authors

Complete this checklist by entering the page numbers from your manuscript where readers will find each of the items listed below.

Your article may not currently address all the items on the checklist. Please modify your text to include the missing information. If you are certain that an item does not apply, please write "n/a" and provide a short explanation.

Upload your completed checklist as an extra file when you submit to a journal.

In your methods section, say that you used the SPIRITreporting guidelines, and cite them as:

Chan A-W, Tetzlaff JM, Gøtzsche PC, Altman DG, Mann H, Berlin J, Dickersin K, Hróbjartsson A, Schulz KF, Parulekar WR, Krleža-Jerić K, Laupacis A, Moher D. SPIRIT 2013 Explanation and Elaboration: Guidance for protocols of clinical trials. BMJ. 2013;346:e7586

		Reporting Item	Page Number
**Administrative information**			
Title	#1	Descriptive title identifying the study design, population, interventions, and, if applicable, trial acronym	1
Trial registration	#2a	Trial identifier and registry name. If not yet registered,	4/6

For peer review only

		name of intended registry	
Trial registration: data set	#2b	All items from the World Health Organization Trial Registration Data Set	4/6
Protocol version	#3	Date and version identifier	5
Funding	#4	Sources and types of financial, material, and other support	3
Roles and responsibilities: contributorship	#5a	Names, affiliations, and roles of protocol contributors	2
Roles and responsibilities: sponsor contact information	#5b	Name and contact information for the trial sponsor	2
Roles and responsibilities: sponsor and funder	#5c	Role of study sponsor and funders, if any, in study design; collection, management, analysis, and interpretation of data; writing of the report; and the decision to submit the report for publication, including whether they will have ultimate authority over any of these activities	2
Roles and responsibilities: committees	#5d	Composition, roles, and responsibilities of the coordinating centre, steering committee, endpoint adjudication committee, data management team, and other individuals or groups overseeing the trial, if applicable (see Item 21a for data monitoring committee)	2

Introduction

For peer review only

Background and rationale	#6a	Description of research question and justification for undertaking the trial, including summary of relevant studies (published and unpublished) examining benefits and harms for each intervention	8-9
Background and rationale: choice of comparators	#6b	Explanation for choice of comparators	8-9
Objectives	#7	Specific objectives or hypotheses	10
Trial design	#8	Description of trial design including type of trial (eg, parallel group, crossover, factorial, single group), allocation ratio, and framework (eg, superiority, equivalence, non-inferiority, exploratory)	10
**Methods: Participants, interventions, and outcomes**			
Study setting	#9	Description of study settings (eg, community clinic, academic hospital) and list of countries where data will be collected. Reference to where list of study sites can be obtained	10
Eligibility criteria	#10	Inclusion and exclusion criteria for participants. If applicable, eligibility criteria for study centres and individuals who will perform the interventions (eg,	11-12

For peer review only

		surgeons, psychotherapists)	
Interventions: description	#11a	Interventions for each group with sufficient detail to allow replication, including how and when they will be administered	13-14
Interventions: modifications	#11b	Criteria for discontinuing or modifying allocated interventions for a given trial participant (eg, drug dose change in response to harms, participant request, or improving / worsening disease)	13-14
Interventions: adherance	#11c	Strategies to improve adherence to intervention protocols, and any procedures for monitoring adherence (eg, drug tablet return; laboratory tests)	13-14
Interventions: concomitant care	#11d	Relevant concomitant care and interventions that are permitted or prohibited during the trial	13-14
Outcomes	#12	Primary, secondary, and other outcomes, including the specific measurement variable (eg, systolic blood pressure), analysis metric (eg, change from baseline, final value, time to event), method of aggregation (eg, median, proportion), and time point for each outcome. Explanation of the clinical relevance of chosen efficacy and harm outcomes is strongly recommended	14-19
Participant timeline	#13	Time schedule of enrolment, interventions (including any run-ins and washouts), assessments, and visits for participants. A schematic diagram is highly recommended (see Figure)	20-21

For peer review only

Sample size	#14	Estimated number of participants needed to achieve study objectives and how it was determined, including clinical and statistical assumptions supporting any sample size calculations	19
Recruitment	#15	Strategies for achieving adequate participant enrolment to reach target sample size	10-11
**Methods: Assignment of interventions (for controlled trials)**			
Allocation: sequence generation	#16a	Method of generating the allocation sequence (eg, computer-generated random numbers), and list of any factors for stratification. To reduce predictability of a random sequence, details of any planned restriction (eg, blocking) should be provided in a separate document that is unavailable to those who enrol participants or assign interventions	12-13
Allocation concealment mechanism	#16b	Mechanism of implementing the allocation sequence (eg, central telephone; sequentially numbered, opaque, sealed envelopes), describing any steps to conceal the sequence until interventions are assigned	12-13
Allocation: implementation	#16c	Who will generate the allocation sequence, who will enrol participants, and who will assign participants to interventions	12-13

For peer review only

Blinding (masking)	#17a	Who will be blinded after assignment to interventions (eg, trial participants, care providers, outcome assessors, data analysts), and how	12-13
Blinding (masking): emergency unblinding	#17b	If blinded, circumstances under which unblinding is permissible, and procedure for revealing a participant's allocated intervention during the trial	12-13
**Methods: Data collection, management, and analysis**			
Data collection plan	#18a	Plans for assessment and collection of outcome, baseline, and other trial data, including any related processes to promote data quality (eg, duplicate measurements, training of assessors) and a description of study instruments (eg, questionnaires, laboratory tests) along with their reliability and validity, if known. Reference to where data collection forms can be found, if not in the protocol	14, 19-21
Data collection plan: retention	#18b	Plans to promote participant retention and complete follow-up, including list of any outcome data to be collected for participants who discontinue or deviate from intervention protocols	14, 19-21
Data management	#19	Plans for data entry, coding, security, and storage, including any related processes to promote data quality (eg, double data entry; range checks for data values).	14, 19-21

For peer review only

		Reference to where details of data management procedures can be found, if not in the protocol	
Statistics: outcomes	#20a	Statistical methods for analysing primary and secondary outcomes. Reference to where other details of the statistical analysis plan can be found, if not in the protocol	19-20
Statistics: additional analyses	#20b	Methods for any additional analyses (eg, subgroup and adjusted analyses)	19-20
Statistics: analysis population and missing data	#20c	Definition of analysis population relating to protocol non-adherence (eg, as randomised analysis), and any statistical methods to handle missing data (eg, multiple imputation)	19-20
**Methods: Monitoring**			
Data monitoring: formal committee	#21a	Composition of data monitoring committee (DMC); summary of its role and reporting structure; statement of whether it is independent from the sponsor and competing interests; and reference to where further details about its charter can be found, if not in the protocol. Alternatively, an explanation of why a DMC is not needed	14, 19-21
Data monitoring: interim analysis	#21b	Description of any interim analyses and stopping guidelines, including who will have access to these interim results and make the final decision to terminate the trial	14, 19-21
Harms	#22	Plans for collecting, assessing, reporting, and managing solicited and spontaneously reported adverse events and other unintended effects of trial interventions or trial	19

For peer review only

		conduct	
Auditing	#23	Frequency and procedures for auditing trial conduct, if any, and whether the process will be independent from investigators and the sponsor	19
**Ethics and dissemination**			
Research ethics approval	#24	Plans for seeking research ethics committee / institutional review board (REC / IRB) approval	21
Protocol amendments	#25	Plans for communicating important protocol modifications (eg, changes to eligibility criteria, outcomes, analyses) to relevant parties (eg, investigators, REC / IRBs, trial participants, trial registries, journals, regulators)	21
Consent or assent	#26a	Who will obtain informed consent or assent from potential trial participants or authorised surrogates, and how (see Item 32)	21
Consent or assent: ancillary studies	#26b	Additional consent provisions for collection and use of participant data and biological specimens in ancillary studies, if applicable	21
Confidentiality	#27	How personal information about potential and enrolled participants will be collected, shared, and maintained in order to protect confidentiality before, during, and after the trial	21
Declaration of	#28	Financial and other competing interests for principal	21

For peer review only

interests		investigators for the overall trial and each study site	
Data access	#29	Statement of who will have access to the final trial dataset, and disclosure of contractual agreements that limit such access for investigators	14, 19-21
Ancillary and post trial care	#30	Provisions, if any, for ancillary and post-trial care, and for compensation to those who suffer harm from trial participation	19-21
Dissemination policy: trial results	#31a	Plans for investigators and sponsor to communicate trial results to participants, healthcare professionals, the public, and other relevant groups (eg, via publication, reporting in results databases, or other data sharing arrangements), including any publication restrictions	19-21
Dissemination policy: authorship	#31b	Authorship eligibility guidelines and any intended use of professional writers	19-21
Dissemination policy: reproducible research	#31c	Plans, if any, for granting public access to the full protocol, participant-level dataset, and statistical code	19-21
**Appendices**			
Informed consent materials	#32	Model consent form and other related documentation given to participants and authorised surrogates	21
Biological specimens	#33	Plans for collection, laboratory evaluation, and storage of biological specimens for genetic or molecular analysis in the current trial and for future use in ancillary studies, if applicable	/

For peer review only

None The SPIRIT Explanation and Elaboration paper is distributed under the terms of the Creative Commons Attribution License CC-BY-NC. This checklist can be completed online using https://www.goodreports.org/, a tool made by the EQUATOR Network in collaboration with Penelope.ai

For peer review only
